# Supplementary material for: Localized cardiac small molecule trajectories and persistent chemical sequelae in experimental Chagas disease
Source: Nat Commun. 2023 Oct 25;14:6769. doi: 10.1038/s41467-023-42247-w (PMC10600178; doi:10.1038/s41467-023-42247-w)
Supplement: Supplementary file 1 — Supplementary Information [file 41467_2023_42247_MOESM1_ESM.pdf]

Supplementary Information for: “Localized cardiac small molecule trajectories and persistent chemical sequelae in experimental Chagas disease”

Zongyuan Liu[1,2], Rebecca Ulrich vonBargen[2,3], April L. Kendricks[4], Kate Wheeler[5], Ana Carolina Leão[6], Krithivasan Sankaranarayanan[2,7], Danya A. Dean[1,2], Shelley S. Kane[1,2], Ekram Hossain[1,2], Jeroen Pollet[6], Maria Elena Bottazzi[6,8], Peter J. Hotez[6,8], Kathryn M. Jones[6,8] *, Laura-Isobel McCall[1,2,7,9] *

[1]Department of Chemistry and Biochemistry, University of Oklahoma, Norman, OK, United States of America

[2]Laboratories of Molecular Anthropology and Microbiome Research, University of Oklahoma, Norman, OK, United States of America

[3]Department of Biomedical Engineering, University of Oklahoma, Norman, OK, United States of America

[4]Southern Star Medical Research Institute, Houston, TX, United States of America

[5]Department of Biology, University of Oklahoma, Norman, OK, United States of America

[6]Department of Pediatrics, Baylor College of Medicine, Houston, TX, United States of America

[7]Department of Microbiology and Plant Biology, University of Oklahoma, Norman, OK, United States of America

[8]Department of Molecular Virology and Microbiology, Baylor College of Medicine, Houston, TX, United States of America

[9]Present affiliation: Department of Chemistry and Biochemistry, San Diego State University, San Diego, CA, United States of America

* Correspondence to: Kathryn Jones (kathrynj@bcm.edu) or Laura-Isobel McCall (Lmccall@sdsu.edu)

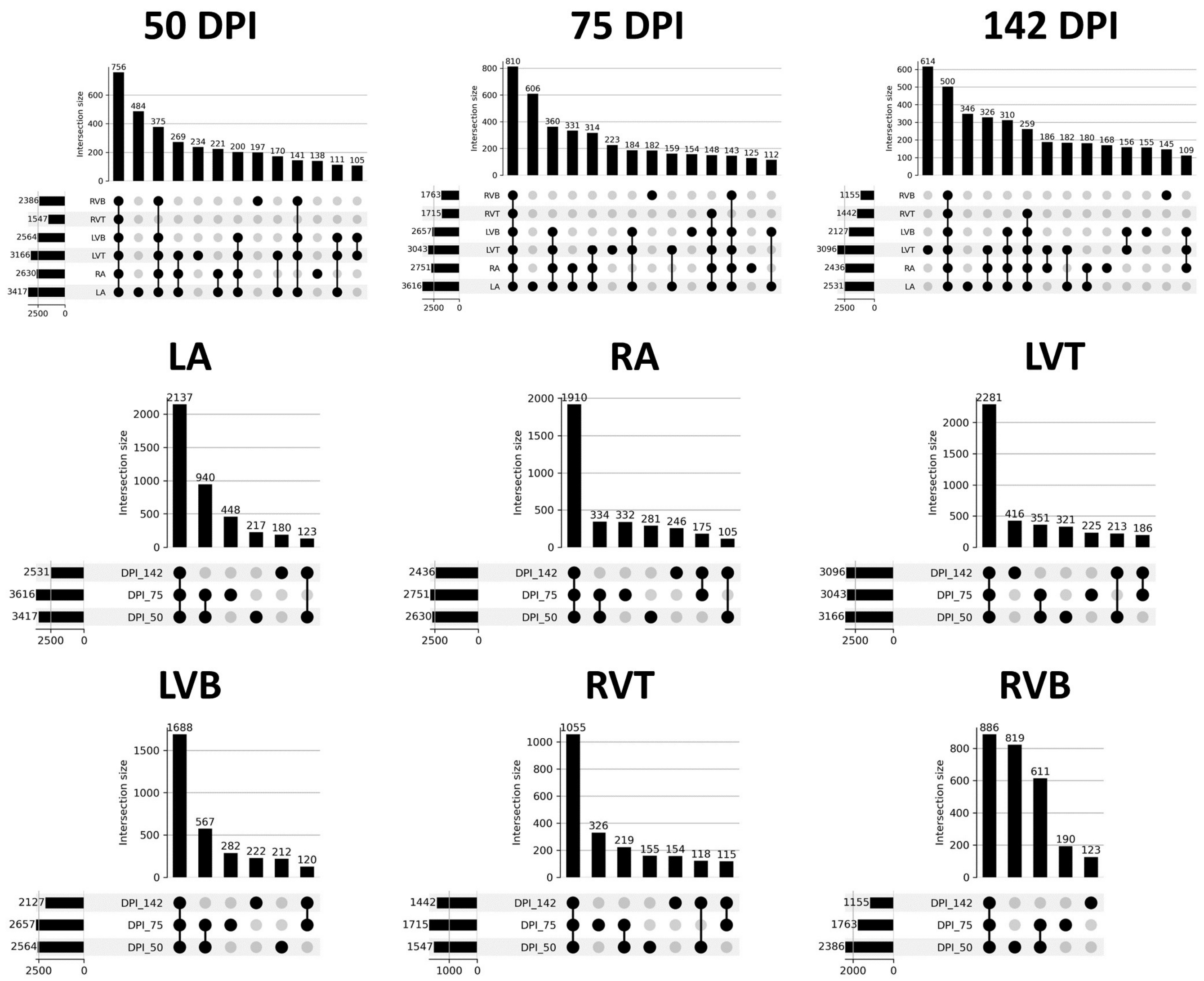

Supplementary Figure 1. UpSet plot of overlap between detected small molecules across 6 heart sections and timepoints. RA, right atrium. LA, left atrium. RVT, right ventricle top. RVB, right ventricle bottom. LVT, left ventricle top. LVB, left ventricle bottom. DPI, days post-infection. N=15 mice per group and per position. Source data are provided as a Source Data file.

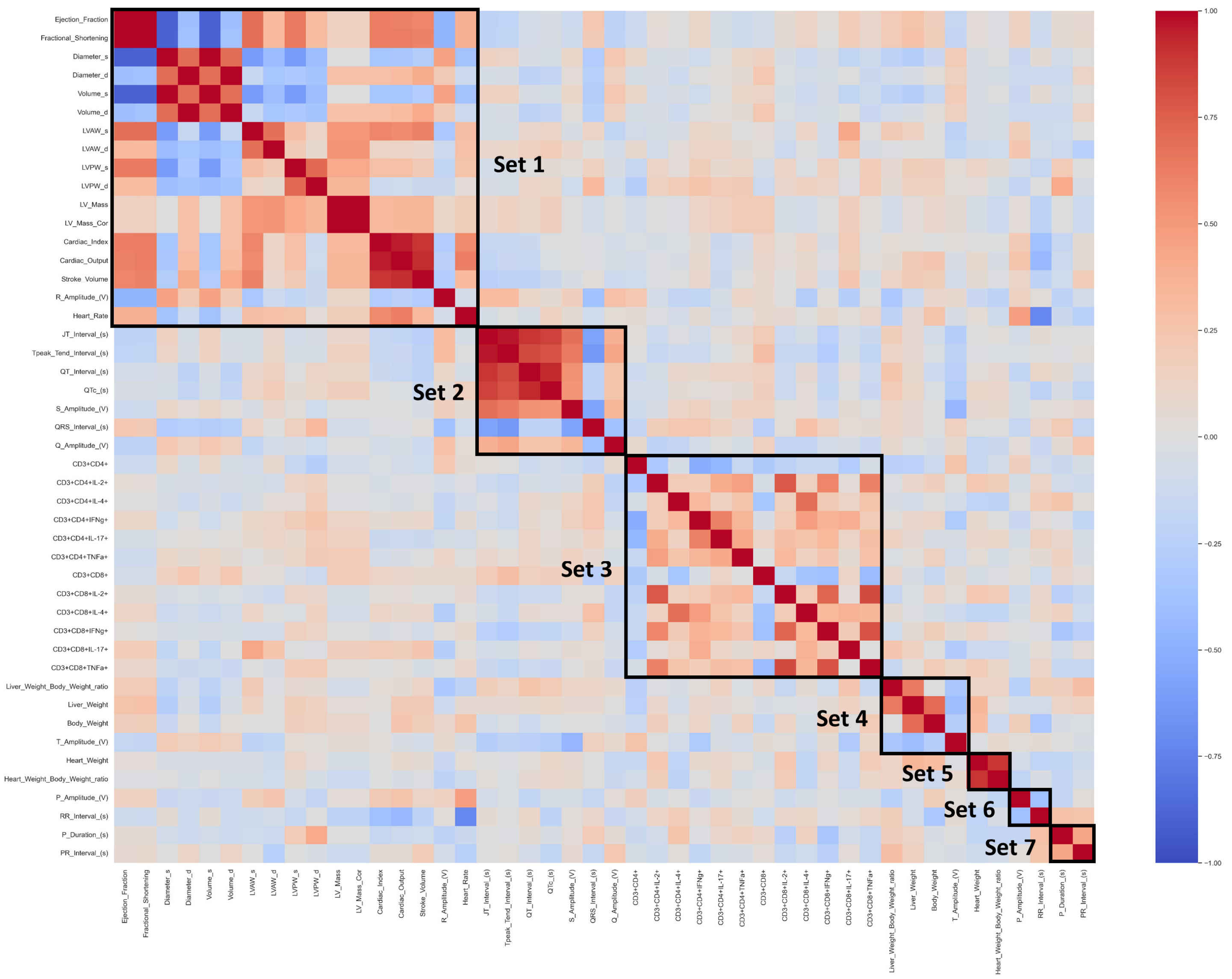

Supplementary Figure 2. Correlation between disease parameters. Source data are provided as a Source Data file.

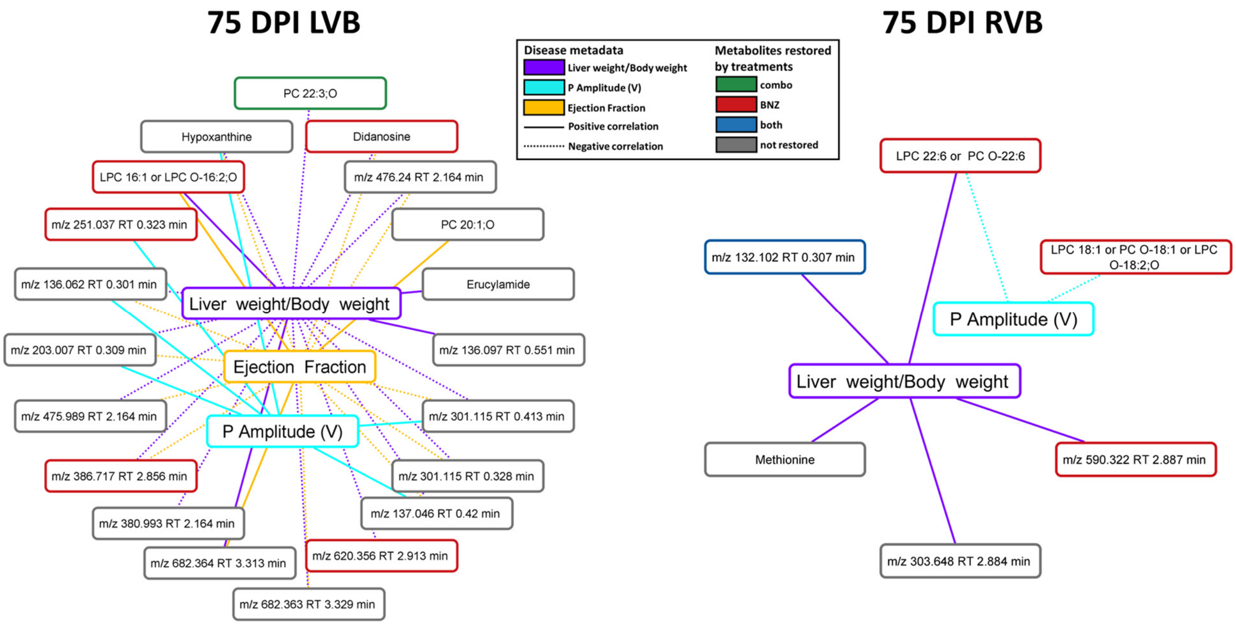

Supplementary Figure 3. Correlation between disease metadata and small molecule peak area at 75 DPI. Solid lines indicate a positive correlation coefficient between disease metadata and small molecules; dotted lines indicate a negative correlation coefficient between disease metadata and small molecules. Correlation line colors relate to the specific correlated metadata category. Small molecule box colors indicate feature restoration status: green for features restored by combo treatment, red for features restored by BNZ treatment, blue for features restored by both treatments, and grey for features not restored by any treatment. Note the greater number of not-restored features correlated to the metadata in LVB. RVB, right ventricle bottom. LVB, left ventricle bottom. DPI, days post-infection. N=15 mice per group and per position. Source data are provided as a Source Data file.

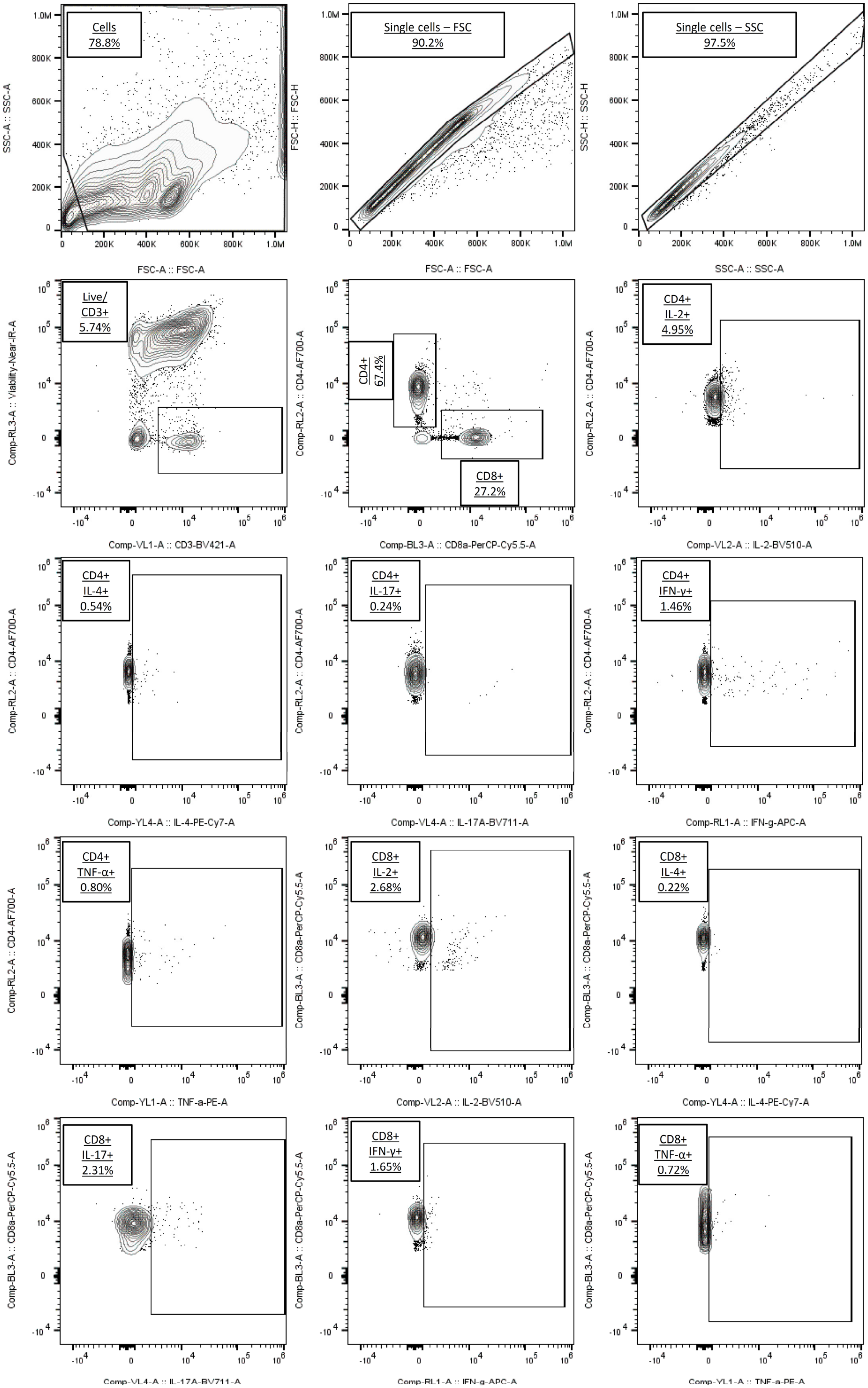
Cells
78.8%
SSC-A :: SSC-A
FSC-A :: FSC-A
Single cells – FSC
90.2%
FSC-H :: FSC-H
FSC-A :: FSC-A
Single cells – SSC
97.5%
SSC-H :: SSC-H
SSC-A :: SSC-A
Live/
CD3+
5.74%
Comp-RL3-A :: Viability-Near-IR-A
Comp-VL1-A :: CD3-BV421-A
CD4+
67.4%
CD8+
27.2%
Comp-RL2-A :: CD4-AF700-A
Comp-BL3-A :: CD8a-PerCP-Cy5.5-A
CD4+
IL-2+
4.95%
Comp-VL2-A :: IL-2-BV510-A
CD4+
IL-4+
0.54%
Comp-YL4-A :: IL-4-PE-Cy7-A
CD4+
IL-17+
0.24%
Comp-VL4-A :: IL-17A-BV711-A
CD4+
IFN-γ+
1.46%
Comp-RL1-A :: IFN-g-APC-A
CD4+
TNF-α+
0.80%
Comp-YL1-A :: TNF-a-PE-A
CD8+
IL-2+
2.68%
Comp-VL2-A :: IL-2-BV510-A
CD8+
IL-4+
0.22%
Comp-YL4-A :: IL-4-PE-Cy7-A
CD8+
IL-17+
2.31%
Comp-VL4-A :: IL-17A-BV711-A
CD8+
IFN-γ+
1.65%
Comp-RL1-A :: IFN-g-APC-A
CD8+
TNF-α+
0.72%
Comp-YL1-A :: TNF-a-PE-A

Supplementary Figure 4. Representative flow plots of gating strategy. Splenocytes restimulated in vitro as described were acquired on a LSR Fortessa Cell Analyzer (BD Biosciences), collecting a minimum of 25,000 total events in a live gate for analysis. Analysis gates were set on live CD3+CD4+ or live CD3+CD8+ cells. Gates to enumerate cytokine-producing cells were set using the FMO strategy as described. All data was analyzed using FlowJo 10.8.1 software as described.

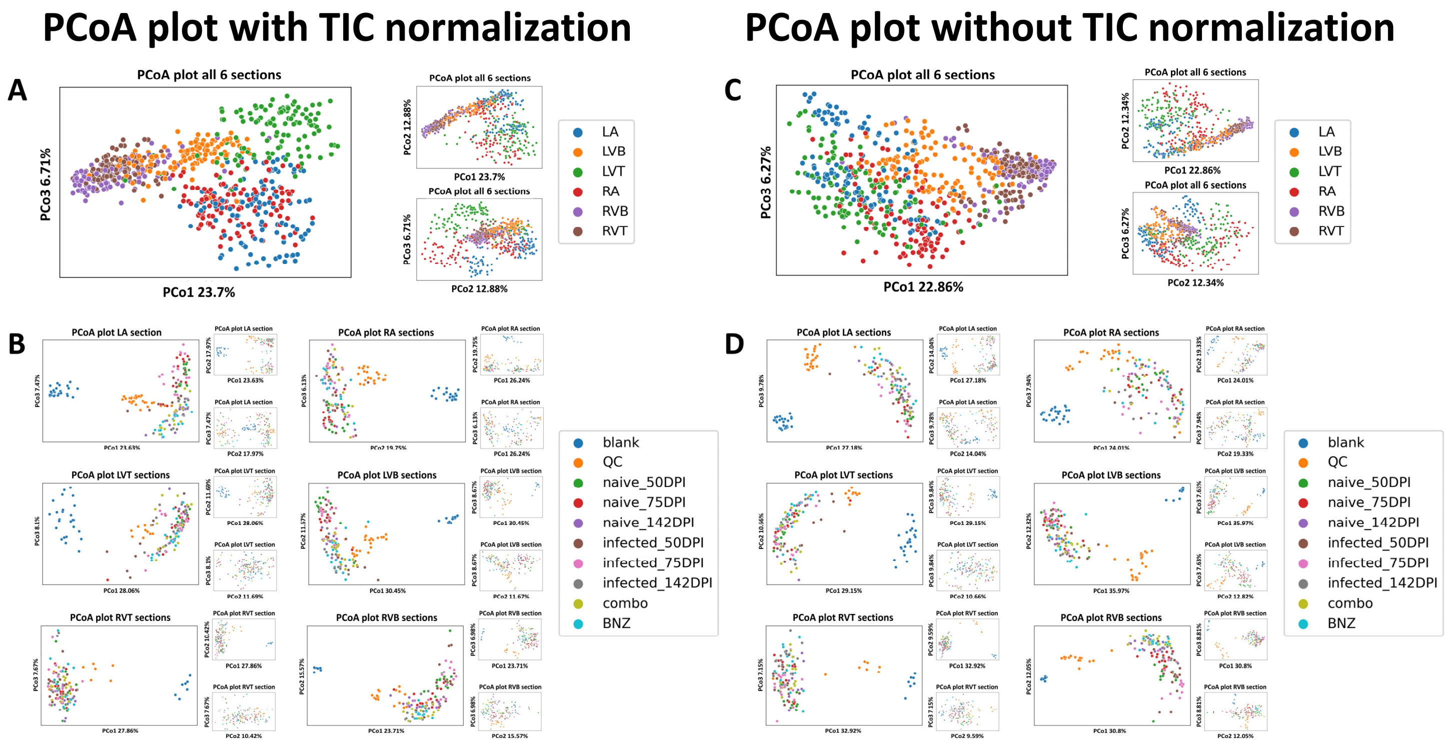

Supplementary Figure 5. Method evaluation principal coordinate analysis plots (PCoAs). (A), (B) TIC-normalized data, as implemented throughout this manuscript. (C), (D) Data prior to TIC normalization. (A), (C) Data from all six sampling sites. (B), (D) Data subset by sampling site, including blanks and QCs. RA, right atrium. LA, left atrium. RVT, right ventricle top. RVB, right ventricle bottom. LVT, left ventricle top. LVB, left ventricle bottom. DPI, days post-infection. BNZ, benznidazole. TIC, total ion current. N=15 mice per group and per position. Source data are provided as a Source Data file.

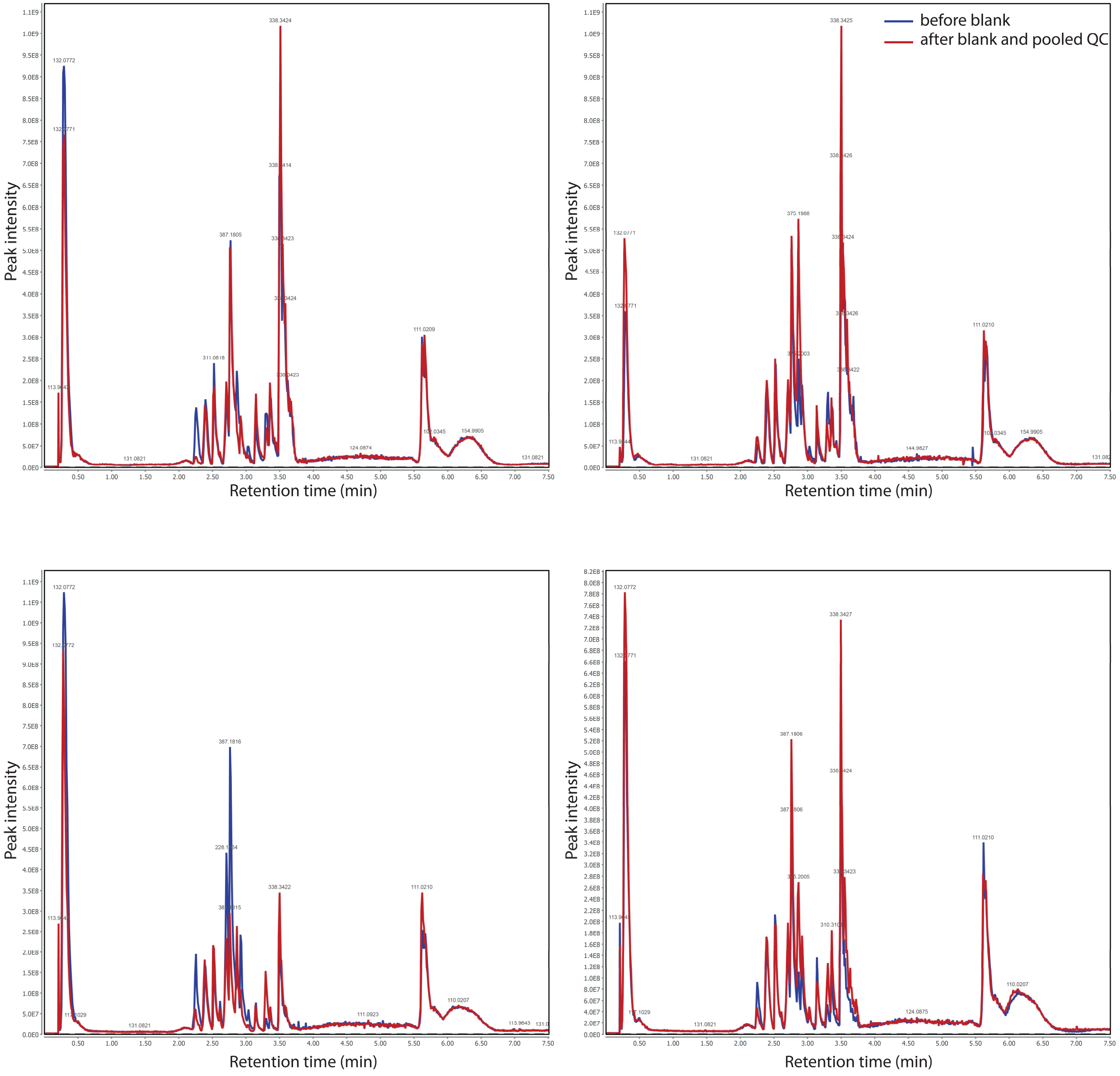

Supplementary Figure 6. No visible impact of blank injection followed by pooled quality control (QC) on retention time in the subsequent sample. Representative total ion chromatograms from four different pairs of samples from the right ventricle top, with the first sample immediately prior to a blank and the second sample immediately subsequent to a blank and a pooled QC. Note that different peak intensities and some differences in peak presence/absence are expected, since these are different biological samples, from different experimental groups (since run order was randomized).

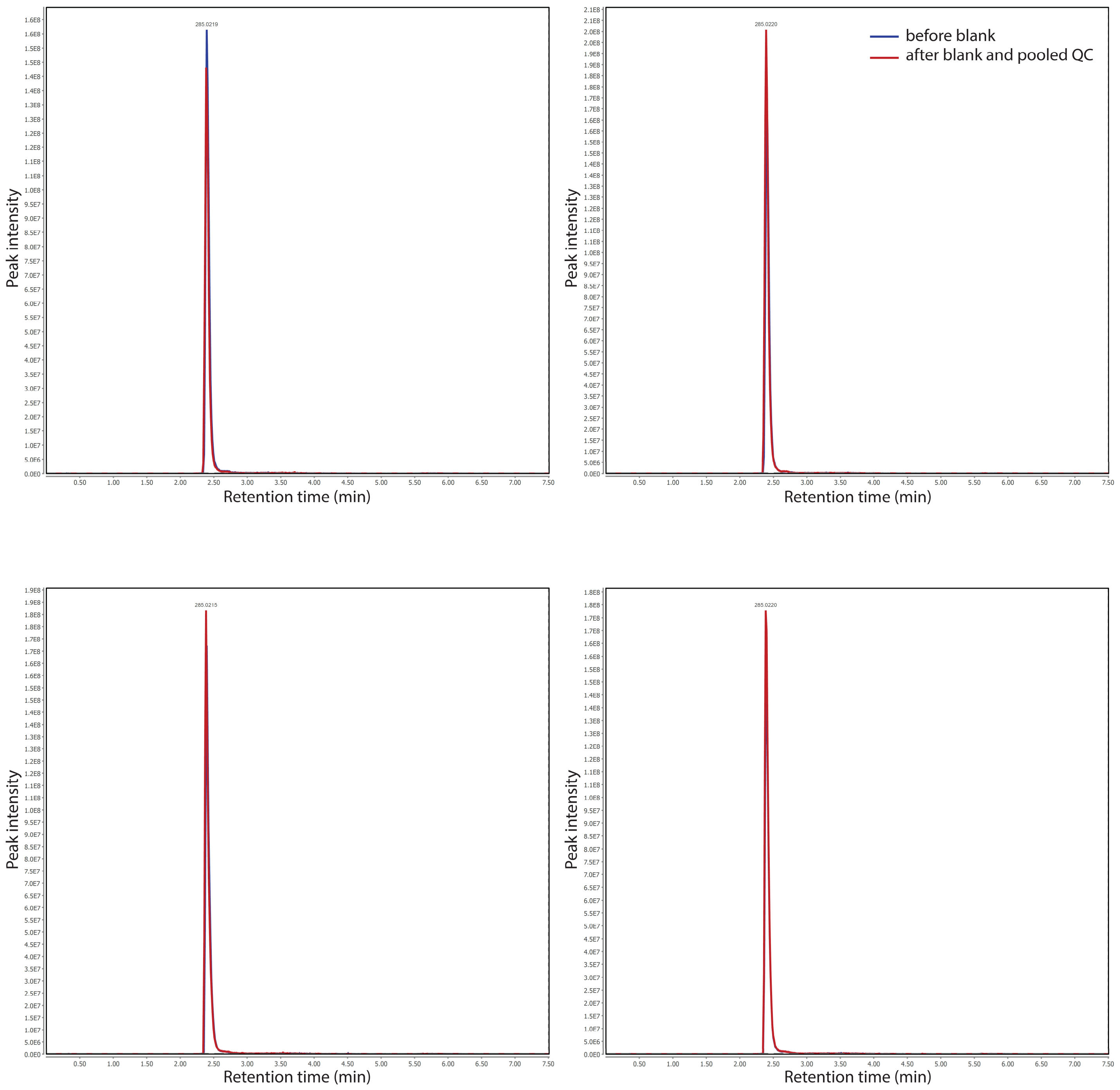

Supplementary Figure 7. No major impact of blank injection followed by pooled quality control (QC) on peak area and retention time in the subsequent sample (internal standard). Representative extracted ion chromatogram for the sulfachloropyridazine internal standard, from four different pairs of samples from the right ventricle top, with the first sample immediately prior to a blank and the second sample immediately subsequent to a blank and a pooled QC.

Supplementary Table 1. Differential metabolic modules by RNA-seq. LFC, log fold change. BvsU, benznidazole-treated vs uninfected. VvsU, vehicle vs uninfected. LRT, Likelihood ratio test. N=5/group. Likelihood-ratio test to determine if "condition" has an effect on Pathway Module counts. Pairwise Wald tests to determine which condition "levels" contribute to the difference. All tests are two-sided. P-value correction: Benjamini-Hochberg False Discovery Rate (BH FDR). Source data are provided as a Source Data file.

BNZ vs uninfected						
**Module**	**Pval_LRT**	**Padj_LRT**	**BaseMean**	**LFC_BvsU**	**Padj_BvsU**	**Pathway Name**
M00151	2.54E-05	0.000174	343048.7	-0.2841	0.049671	Cytochrome bc1 complex respiratory unit
M00152	7.67E-05	0.00043	420893.2	-0.2705	0.04761	Cytochrome bc1 complex
M00130	0.007058	0.017281	14230.19	0.168485	0.024977	Inositol phosphate metabolism, PI=> PIP2 => Ins(1,4,5)P3 => Ins(1,3,4,5)P4
M00093	0.000884	0.002989	5576.449	0.232356	0.019257	Phosphatidylethanolamine (PE) biosynthesis, PA => PS => PE
M00071	6.69E-08	1.10E-06	3097.112	0.251196	0.020803	Glycosphingolipid biosynthesis, neolacto-series, LacCer => nLc4Cer
M00057	1.12E-06	1.40E-05	1848.078	0.259915	0.024977	Glycosaminoglycan biosynthesis, linkage tetrasaccharide
M00100	0.000581	0.002132	1503.144	0.286532	0.036989	Sphingosine degradation
M00074	0.003001	0.009002	5879.333	0.288238	0.014359	N-glycan biosynthesis, high-mannose type
M00058	4.17E-05	0.000261	3084.596	0.290719	0.036333	Glycosaminoglycan biosynthesis, chondroitin sulfate backbone
M00073	0.002019	0.006615	6354.071	0.300851	0.011562	N-glycan precursor trimming
M00066	1.81E-05	0.000141	1818.825	0.304505	0.016102	Lactosylceramide biosynthesis
M00078	0.000131	0.000698	6998.677	0.312085	0.016102	Heparan sulfate degradation
M00415	0.009901	0.021519	6192.173	0.320747	0.02387	Fatty acid elongation in endoplasmic reticulum
M00008	0.003168	0.009371	2665.847	0.341653	0.036989	Entner-Doudoroff pathway, glucose-6P => glyceraldehyde-3P + pyruvate
M00079	7.19E-06	6.96E-05	7297.447	0.347735	0.016857	Keratan sulfate degradation
M00006	0.003598	0.010357	4061.24	0.385459	0.038108	Pentose phosphate pathway, oxidative phase, glucose 6P => ribulose 5P
M00176	0.005224	0.013736	1066.067	0.418453	0.017014	Assimilatory sulfate reduction, sulfate => H2S
M00616	0.005224	0.013736	1066.067	0.418453	0.017014	Sulfate-sulfur assimilation
M00914	0.007284	0.017432	436.6947	0.486915	0.036333	Coenzyme A biosynthesis, archaea, 2-oxoisovalerate => 4-phosphopantoate => CoA
M00958	6.31E-10	1.92E-08	7673.606	0.520856	1.87E-05	Adenine ribonucleotide degradation, AMP => Urate

M00068	2.13E-05	0.000156	308.8995	0.538228	0.015835	Glycosphingolipid biosynthesis, globo-series, LacCer => Gb4Cer
M00959	1.20E-10	6.39E-09	7354.254	0.557602	2.01E-05	Guanine ribonucleotide degradation, GMP => Urate
M00038	1.05E-05	9.33E-05	428.7265	0.592959	0.014359	Tryptophan metabolism, tryptophan => kynurenine => 2-aminomuconate
M00844	1.10E-08	1.95E-07	632.1606	0.672359	0.014879	Arginine biosynthesis, ornithine => arginine
M00845	1.10E-08	1.95E-07	632.1606	0.672359	0.014879	Arginine biosynthesis, glutamate => acetylcitrulline => arginine
M00546	5.11E-12	3.63E-10	4059.72	0.699109	2.03E-06	Purine degradation, xanthine => urea
M00107	1.81E-12	3.63E-10	105.602	0.758362	0.001222	Steroid hormone biosynthesis, cholesterol => pregnenolone => progesterone
M00029	4.63E-10	1.64E-08	695.0693	0.779278	0.009111	Urea cycle
M00110	4.39E-12	3.63E-10	101.3123	0.782244	0.001222	C19/C18-Steroid hormone biosynthesis, pregnenolone => androstenedione => estrone
M00045	1.39E-05	0.000119	262.393	0.981127	0.009413	Histidine degradation, histidine => N-formiminoglutamate => glutamate

vehicle vs uninfected

**Module**	**Pval_LRT**	**Padj_LRT**	**BaseMean**	**LFC_VvsU**	**Padj_VvsU**	**Pathway Name**
M00017	0.003424	0.009992	4426.346	-0.78813	0.002325	Methionine biosynthesis, aspartate => homoserine => methionine
M00142	4.10E-05	0.000261	360577.2	-0.7596	4.16E-05	NADH:ubiquinone oxidoreductase, mitochondria
M00086	4.42E-07	6.28E-06	15716.83	-0.72498	1.32E-06	beta-Oxidation, acyl-CoA synthesis
M00565	1.07E-06	1.40E-05	1136.362	-0.6512	4.02E-06	Trehalose biosynthesis, D-glucose 1P => trehalose
M00154	0.000105	0.000572	2070665	-0.64991	9.75E-05	Cytochrome c oxidase
M00027	1.69E-05	0.000138	2157.459	-0.58382	2.15E-05	GABA (gamma-Aminobutyrate) shunt
M00151	2.54E-05	0.000174	343048.7	-0.58118	3.62E-05	Cytochrome bc1 complex respiratory unit
M00122	3.82E-06	4.28E-05	784.6708	-0.56072	1.30E-05	Cobalamin biosynthesis, cobyrinate a,c-diamide => cobalamin
M00307	4.70E-05	0.000286	50772.69	-0.55619	5.25E-05	Pyruvate oxidation, pyruvate => acetyl-CoA
M00532	5.23E-06	5.57E-05	16042.09	-0.53991	1.52E-05	Photorespiration
M00036	2.75E-05	0.000183	66517.41	-0.53984	3.09E-05	Leucine degradation, leucine => acetoacetate + acetyl-CoA
M00357	0.005189	0.013736	6108.046	-0.50562	0.003622	Methanogenesis, acetate => methane
M00152	7.67E-05	0.00043	420893.2	-0.50271	0.000109	Cytochrome bc1 complex
M00373	0.000204	0.00101	73180.97	-0.48503	0.000196	Ethylmalonyl pathway

M00375	0.000284	0.001288	65625.56	-0.47804	0.000252	Hydroxypropionate-hydroxybutylate cycle
M00374	0.000234	0.001108	108133.3	-0.4725	0.000215	Dicarboxylate-hydroxybutyrate cycle
M00013	1.86E-05	0.000141	29269.6	-0.47027	3.03E-05	Malonate semialdehyde pathway, propanoyl-CoA => acetyl-CoA
M00957	0.000239	0.001109	77288.69	-0.47001	0.000215	Lysine degradation, bacteria, L-lysine => glutarate => succinate/acetyl-CoA
M00620	5.59E-06	5.67E-05	7714.984	-0.46995	9.85E-06	Incomplete reductive citrate cycle, acetyl-CoA => oxoglutarate
M00149	0.000827	0.00284	35765.74	-0.46531	0.000564	Succinate dehydrogenase, prokaryotes
M00849	0.000308	0.001341	66838.57	-0.46322	0.000257	C5 isoprenoid biosynthesis, mevalonate pathway, archaea
M00956	0.006822	0.017053	976.6508	-0.46218	0.00444	Lysine degradation, bacteria, L-lysine => succinate
M00095	0.000302	0.001339	67295.5	-0.45944	0.000257	C5 isoprenoid biosynthesis, mevalonate pathway
M00087	0.000404	0.001688	179117	-0.45251	0.000334	beta-Oxidation
M00085	0.000722	0.002523	93253.46	-0.45121	0.00051	Fatty acid elongation in mitochondria
M00376	0.000629	0.002233	47084.39	-0.44966	0.000452	3-Hydroxypropionate bi-cycle
M00088	0.000454	0.001825	72125.04	-0.44239	0.000361	Ketone body biosynthesis, acetyl-CoA => acetoacetate/3-hydroxybutyrate/acetone
M00032	0.00045	0.001825	62833.11	-0.43733	0.000361	Lysine degradation, lysine => saccharopine => acetoacetyl-CoA
M00613	0.000484	0.00184	51732.22	-0.43693	0.00037	Anoxygenic photosynthesis in green nonsulfur bacteria
M00155	0.041833	0.070161	355227.8	-0.42837	0.024487	Cytochrome c oxidase, prokaryotes
M00011	0.002882	0.008898	134490.6	-0.42263	0.002	Citrate cycle, second carbon oxidation, 2-oxoglutarate => oxaloacetate
M00855	0.007278	0.017432	51144.04	-0.4157	0.0048	Glycogen degradation, glycogen => glucose-6P
M00878	0.000477	0.00184	10512.96	-0.39524	0.00037	Phenylacetate degradation, phenylaxetate => acetyl-CoA/succinyl-CoA
M00741	8.27E-06	7.66E-05	8528.39	-0.39186	1.56E-05	Propanoyl-CoA metabolism, propanoyl-CoA => succinyl-CoA
M00158	0.002672	0.008369	404747.4	-0.38986	0.002088	F-type ATPase, eukaryotes
M00148	0.002967	0.009002	55343.39	-0.37889	0.002088	Succinate dehydrogenase (ubiquinone)
M00009	0.004753	0.013097	214482.4	-0.37657	0.0032	Citrate cycle (TCA cycle, Krebs cycle)
M00168	0.003797	0.010783	21085.14	-0.37366	0.002788	CAM (Crassulacean acid metabolism), dark
M00171	0.017188	0.032983	56061.23	-0.34656	0.012581	C4-dicarboxylic acid cycle, NAD - malic enzyme type

M00173	0.006345	0.01609	84582.24	-0.33112	0.004499	Reductive citrate cycle (Arnon-Buchanan cycle)
M00614	0.006345	0.01609	84582.24	-0.33112	0.004499	Anoxygenic photosynthesis in green sulfur bacteria
M00170	0.039848	0.067362	33350.04	-0.32269	0.032146	C4-dicarboxylic acid cycle, phosphoenolpyruvate carboxykinase type
M00012	0.012425	0.026204	49105.71	-0.32132	0.007706	Glyoxylate cycle
M00010	0.011894	0.025335	79991.85	-0.32099	0.007706	Citrate cycle, first carbon oxidation, oxaloacetate => 2-oxoglutarate
M00157	0.015044	0.029947	193456.8	-0.32052	0.009566	F-type ATPase, prokaryotes and chloroplasts
M00115	0.013229	0.027625	852.1475	-0.30661	0.009306	NAD biosynthesis, aspartate => quinolinate => NAD
M00344	0.058319	0.090671	440.5014	-0.26699	0.03779	Formaldehyde assimilation, xylulose monophosphate pathway
M00862	0.000329	0.001401	8487.699	-0.25889	0.000475	beta-Oxidation, peroxisome, tri/dihydroxycholestanoyl-CoA => choloyl/chenodeoxycholoyl-CoA
M00169	0.000152	0.000771	4647.983	-0.25566	0.000452	CAM (Crassulacean acid metabolism), light
M00172	0.000152	0.000771	4647.983	-0.25566	0.000452	C4-dicarboxylic acid cycle, NADP - malic enzyme type
M00861	0.00054	0.002019	16760.82	-0.25109	0.00051	beta-Oxidation, peroxisome, VLCFA
M00113	0.007844	0.017796	8278.844	-0.23412	0.005009	Jasmonic acid biosynthesis
M00128	0.042233	0.070279	8925.9	-0.22763	0.026779	Ubiquinone biosynthesis, eukaryotes, 4-hydroxybenzoate + polyprenyl-PP => ubiquinol
M00911	0.016821	0.032714	2823.479	-0.20048	0.010134	Riboflavin biosynthesis, fungi, GTP => riboflavin/FMN/FAD
M00872	0.014632	0.029402	6821.147	-0.19471	0.024552	O-glycan biosynthesis, mannose type (core M3)
M00089	0.000626	0.002233	27332.49	-0.18615	0.021773	Triacylglycerol biosynthesis
M00880	0.007634	0.017675	1713.007	-0.18568	0.018072	Molybdenum cofactor biosynthesis, GTP => molybdenum cofactor
M00874	0.070889	0.10559	20704.09	-0.17085	0.044104	Fatty acid biosynthesis in mitochondria, fungi
M00104	0.020877	0.039006	10716.84	-0.14938	0.026657	Bile acid biosynthesis, cholesterol => cholate/chenodeoxycholate
M00926	0.044461	0.072848	7594.657	-0.14096	0.032105	Heme biosynthesis, bacteria, glutamyl-tRNA => coproporphyrin III => heme
M00130	0.007058	0.017281	14230.19	0.151828	0.033525	Inositol phosphate metabolism, PI=> PIP2 => Ins(1,4,5)P3 => Ins(1,3,4,5)P4
M00055	0.016894	0.032714	4017.397	0.194025	0.012589	N-glycan precursor biosynthesis

M00167	0.007522	0.017606	60411.55	0.253727	0.026931	Reductive pentose phosphate cycle, glyceraldehyde-3P => ribulose-5P
M00892	0.005603	0.014555	29193.78	0.268445	0.005122	UDP-N-acetyl-D-glucosamine biosynthesis, eukaryotes, glucose => UDP-GlcNAc
M00093	0.000884	0.002989	5576.449	0.271454	0.002088	Phosphatidylethanolamine (PE) biosynthesis, PA => PS => PE
M00098	0.020337	0.038676	16846.69	0.271752	0.012378	Acylglycerol degradation
M00909	0.003849	0.010787	14901.34	0.279449	0.004668	UDP-N-acetyl-D-glucosamine biosynthesis, prokaryotes, glucose => UDP-GlcNAc
M00554	0.061861	0.094794	797.8422	0.301364	0.034836	Nucleotide sugar biosynthesis, galactose => UDP-galactose
M00176	0.005224	0.013736	1066.067	0.316188	0.041579	Assimilatory sulfate reduction, sulfate => H2S
M00616	0.005224	0.013736	1066.067	0.316188	0.041579	Sulfate-sulfur assimilation
M00099	0.007408	0.017532	7733.662	0.316587	0.007225	Sphingosine biosynthesis
M00076	0.007854	0.017796	3800.295	0.317158	0.007181	Dermatan sulfate degradation
M00101	0.029578	0.050808	3086.969	0.327782	0.024743	Cholesterol biosynthesis, squalene 2,3-epoxide => cholesterol
M00004	0.024584	0.043275	22759.38	0.33168	0.015904	Pentose phosphate pathway (Pentose phosphate cycle)
M00133	0.004796	0.013097	822.2516	0.333359	0.003321	Polyamine biosynthesis, arginine => agmatine => putrescine => spermidine
M00134	0.006885	0.017053	3354.433	0.371101	0.005442	Polyamine biosynthesis, arginine => ornithine => putrescine
M00917	0.01105	0.023773	2470.922	0.372832	0.008401	Phytosterol biosynthesis, squalene 2,3-epoxide => campesterol/sitosterol
M00094	0.002154	0.00695	5484.512	0.395231	0.00181	Ceramide biosynthesis
M00124	0.023939	0.042491	530.0559	0.410197	0.019188	Pyridoxal-P biosynthesis, erythrose-4P => pyridoxal-P
M00078	0.000131	0.000698	6998.677	0.410717	0.000252	Heparan sulfate degradation
M00160	3.62E-06	4.28E-05	30976.7	0.436716	1.56E-05	V-type ATPase, eukaryotes
M00008	0.003168	0.009371	2665.847	0.439566	0.004668	Entner-Doudoroff pathway, glucose-6P => glyceraldehyde-3P + pyruvate
M00100	0.000581	0.002132	1503.144	0.44016	0.000608	Sphingosine degradation
M00366	0.016347	0.032239	735.0158	0.441401	0.009566	C10-C20 isoprenoid biosynthesis, plants
M00077	0.001581	0.005262	2704.23	0.445693	0.001341	Chondroitin sulfate degradation
M00072	7.28E-05	0.000419	12138.59	0.471691	7.25E-05	N-glycosylation by oligosaccharyltransferase

M00014	0.002307	0.007334	19370.84	0.475614	0.001651	Glucuronate pathway (uronate pathway)
M00066	1.81E-05	0.000141	1818.825	0.478066	3.09E-05	Lactosylceramide biosynthesis
M00006	0.003598	0.010357	4061.24	0.497236	0.004976	Pentose phosphate pathway, oxidative phase, glucose 6P => ribulose 5P
M00103	0.038968	0.066401	4.905227	0.500321	0.027063	Cholecalciferol biosynthesis
M00129	2.62E-07	3.99E-06	18186.38	0.510115	1.92E-06	Ascorbate biosynthesis, animals, glucose-1P => ascorbate
M00057	1.12E-06	1.40E-05	1848.078	0.518723	2.09E-06	Glycosaminoglycan biosynthesis, linkage tetrasaccharide
M00914	0.007284	0.017432	436.6947	0.52366	0.014243	Coenzyme A biosynthesis, archaea, 2-oxoisovalerate => 4-phosphopantoate => CoA
M00071	6.69E-08	1.10E-06	3097.112	0.526583	1.36E-07	Glycosphingolipid biosynthesis, neolacto-series, LacCer => nLc4Cer
M00058	4.17E-05	0.000261	3084.596	0.532032	4.85E-05	Glycosaminoglycan biosynthesis, chondroitin sulfate backbone
M00015	0.000482	0.00184	1020.656	0.547406	0.000424	Proline biosynthesis, glutamate => proline
M00079	7.19E-06	6.96E-05	7297.447	0.588285	1.35E-05	Keratan sulfate degradation
M00042	2.43E-05	0.000173	494.2671	0.593166	0.000285	Catecholamine biosynthesis, tyrosine => dopamine => noradrenaline => adrenaline
M00020	0.022158	0.040339	621.2222	0.59684	0.014322	Serine biosynthesis, glycerate-3P => serine
M00580	0.000229	0.001108	505.7957	0.634739	0.000215	Pentose phosphate pathway, archaea, fructose 6P => ribose 5P
M00958	6.31E-10	1.92E-08	7673.606	0.654272	1.70E-08	Adenine ribonucleotide degradation, AMP => Urate
M00091	0.020701	0.039006	34.93345	0.733937	0.012412	Phosphatidylcholine (PC) biosynthesis, PE => PC
M00959	1.20E-10	6.39E-09	7354.254	0.74196	1.79E-09	Guanine ribonucleotide degradation, GMP => Urate
M00046	6.11E-09	1.44E-07	3493.121	0.782719	2.64E-08	Pyrimidine degradation, uracil => beta-alanine, thymine => 3-aminoisobutanoate
M00068	2.13E-05	0.000156	308.8995	0.859321	3.09E-05	Glycosphingolipid biosynthesis, globo-series, LacCer => Gb4Cer
M00546	5.11E-12	3.63E-10	4059.72	0.875456	2.05E-10	Purine degradation, xanthine => urea
M00038	1.05E-05	9.33E-05	428.7265	0.94483	1.56E-05	Tryptophan metabolism, tryptophan => kynurenine => 2-aminomuconate
M00039	4.96E-05	0.000294	210.8957	0.973986	6.62E-05	Monolignol biosynthesis, phenylalanine/tyrosine => monolignol
M00045	1.39E-05	0.000119	262.393	1.435492	1.56E-05	Histidine degradation, histidine => N-formiminoglutamate => glutamate

M00107	1.81E-12	3.63E-10	105.602	1.449917	3.15E-11	Steroid hormone biosynthesis, cholesterol => pregnenolone => progesterone
M00110	4.39E-12	3.63E-10	101.3123	1.46651	3.74E-11	C19/C18-Steroid hormone biosynthesis, pregnenolone => androstenedione => estrone
M00844	1.10E-08	1.95E-07	632.1606	1.475239	1.80E-08	Arginine biosynthesis, ornithine => arginine
M00845	1.10E-08	1.95E-07	632.1606	1.475239	1.80E-08	Arginine biosynthesis, glutamate => acetylcitrulline => arginine
M00037	4.99E-09	1.33E-07	243.8195	1.542745	2.64E-08	Melatonin biosynthesis, animals, tryptophan => serotonin => melatonin
M00936	6.77E-09	1.44E-07	236.5474	1.569541	3.24E-08	Melatonin biosynthesis, plants, tryptophan => serotonin => melatonin
M00029	4.63E-10	1.64E-08	695.0693	1.630909	1.23E-09	Urea cycle
M00968	1.65E-10	7.05E-09	216.344	1.656426	6.25E-09	Pentose bisphosphate pathway (nucleoside degradation), archaea, nucleoside/NMP => 3-PGA/glycerone phosphate

Supplementary Table 2. Disease parameters median values and interquartile range (supporting data in Table 1). IQR, interquartile range. N=15 mice per group. Source data are provided as a Source Data file.

Disease metadata	naive_median	naive_IQR	infected_median	infected_IQR	combo_median	combo_IQR	BNZ_median	BNZ_IQR
Body Weight	25.2	1.6	24.3	1.95	23.4	1	23.6	1.2
CD3+CD8+IFNg+	0.0046	0.00265	0.0013	0.005	0.003	0.01035	0.0005	0.0046
CD3+CD8+IL-2+	0.0132	0.02215	0.002	0.01505	0.0064	0.04385	0.0017	0.00535
CD3+CD8+TNFa+	0.0055	0.00675	0.0011	0.0073	0.0016	0.0278	0.0001	0.00725
Left Ventricular End Systolic Diameter (millimeters)	2.445012	0.388272	2.214906	0.36785	2.387004	0.449023	2.666671	0.581516
Heart Rate	446.4478	45.92878	405.7345	50.72885	414.4822	29.55705	404.8384	46.08246
Liver Weight/ Body Weight	0.053913	0.003541	0.058228	0.002806	0.0575	0.003424	0.056054	0.002942
Left Ventricular Anterior Wall, systole (mm)	1.531194	0.245886	1.701612	0.171231	1.64556	0.332623	1.424525	0.158953
P Amplitude (V)	0.1537	0.0713	0.106	0.03427	0.08632	0.0509	0.1015	0.041125
P Duration (s)	0.01358	0.00235	0.0143	0.00288	0.01369	0.002045	0.01446	0.001915
QT Interval (s)	0.01703	0.00083	0.01858	0.00288	0.01781	0.00214	0.01806	0.00217
QTc (s)	0.0451	0.002665	0.04886	0.00591	0.04526	0.006045	0.04721	0.00604
R Amplitude (V)	1.357	0.265	0.9077	0.2713	1.075	0.3218	1.267	0.376
RR Interval (s)	0.1392	0.0128	0.1503	0.01055	0.1467	0.0064	0.1464	0.0104
T Amplitude (V)	0.3877	0.13595	0.3667	0.1382	0.3427	0.1629	0.4178	0.11305
End Systolic Volume (microliters)	21.11943	8.729488	16.5908	6.67166	19.89002	8.856307	26.19963	14.92633

Supplementary Table 3. Proportion of small molecules for each treatment response behavior that were significantly perturbed at early chronic stage timepoints. RA, right atrium. LA, left atrium. RVT, right ventricle top. RVB, right ventricle bottom. LVT, left ventricle top. LVB, left ventricle bottom. DPI, days post-infection. BNZ, benznidazole. N=15 mice per group and per position. Source data are provided as a Source Data file.

Timepoint	Sections	Restored by any treatment	Restored by BNZ+Tc24 combination	Restored by BNZ-only treatment	Restored by both treatments	Not restored
50 DPI	LA	0.42	1	0.33	0.38	0.84
	RA	0.1	No small molecules restored by BNZ+Tc24 combination	0	0.11	No small molecules that failed to be restored
	LVT	0.6	0.71	0.5	0.43	0
	LVB	0.76	0.73	0.83	0.75	0.72
	RVT	0.48	0.5	0	0.5	0.31
	RVB	0.07	0	0.25	0	0.03
75 DPI	LA	0	0	0	0	0
	RA	0.1	No small molecules restored by BNZ+Tc24 combination	0.33	0.07	No small molecules that failed to be restored
	LVT	0.12	0	0.5	0.14	0
	LVB	0.28	0.07	0.83	0.25	0.62
	RVT	0.16	0.12	0	0.18	0.28
	RVB	0.14	0	0.38	0.14	0.09

Supplementary Table 4. Instrumental and LC-MS data processing methods. AGC, automatic gain control. IT, ion injection time. (N)CE, (normalized) collision energy.

**Thermo Scientific Vanquish UHPLC system parameters**		
Time (min)	Flow (mL/min)	%A (Water + 0.1% Formic Acid)
0.00	0.500	98
1.00	0.500	98
2.50	0.500	2
4.50	0.500	2
5.50	0.500	98
7.50	0.500	98
7.50	Stop Run	
**Divert valve parameters**		
Switch at	0.2 min	
**Q Exactive Plus (Thermo Scientific) mass spectrometer parameters**		
Source		
Sheath Gas Flow Rate	35	
Aux Gas Flow Rate	10	
Sweep Gas Flow Rate	0	
Spray Voltage	3.80 (+)	
Capillary Temperature	320ºC	
S-lens RF Level	50.0	
Aux Gas Heater Temperature	350.0ºC	
Full MS		
Resolution	70,000	
AGC Target	3.0E6	
Maximum IT	246 milliseconds	
Scan Range	100 to 1500 *m/z*	
dd-MS^2		
Resolution	17,500	
AGC Target	1.0E5	
Maximum IT	54 milliseconds	
Top N	5	
Isolation Window	1.0 *m/z*	
Fixed First Mass	---	
(N)CE/ Stepped	NCE: 20, 40, 60	
dd Settings		
Min. AGC Target	8.00E3	
Intensity Threshold	1.50E5	
Apex Trigger	---	
Charge Exclusion	---	
Peptide Match	preferred	
Exclude Isotope	On	
Dynamic Exclusion	10.0 seconds	
**MZmine version 2.53 parameters**		
		Polarity: Positive

MS^1	Noise Level	9.0E5
MS^2	Noise Level	1.0E3
Chromatogram Builder	Mass List	masses
	Minimum Time Span (min)	0.01
	Minimum Height	9.0E5
	m/z Tolerance (ppm)	10.0
Chromatogram Deconvolution	Algorithm	Local minimum search
	m/z Range for MS^2 Scan Pairing (Da)	0.01
	RT Range for MS^2 Scan Pairing (min)	0.2
	Chromatographic Threshold	20%
	Search Minimum in RT range	0.03
	Minimum Relative Height	26%
	Minimum Absolute Height	1.0E4
	Minimum ratio of peak top/edge	1
	Peak Duration range (min)	0.01-1.00
Deisotoping	*m/z* Tolerance (ppm)	10.0
	Retention Time Tolerance (min)	0.5
	Monotonic Shape	Checked
	Maximum Charge	3
	Representative Isotope	Lowest *m/z*
Alignment	*m/z* Tolerance (ppm)	10.0
	Weight for *m/z*	1
	Weight for Retention Time	1
	Retention Time Tolerance (min)	0.5
Row Filtering	Minimum Peaks in a Row	3
	Retention Time (min)	0.3-7
	Keeps Only Peaks with MS^2 Scans	Checked
	Reset Peak No. ID	Checked

Supplementary Table 5. Distance analysis between naïve and infected at 50, 75, and 142 days post infection (DPI). LA, left atrium. RA, right atrium. LVT, left ventricle top. LVB, left ventricle bottom. RVT, right ventricle top. RVB, right ventricle bottom. N=15 mice per group and per position. Source data are provided as a Source Data file.

Heart section	DPI	pseudo-F	p-value
LA	50	3.029144	0.014
	70	2.330901	0.023
	142	1.09537	0.321
RA	50	1.578709	0.12
	70	2.910362	0.013
	142	2.511789	0.039
LVT	50	3.652951	0.005
	70	1.460853	0.143
	142	1.187019	0.282
LVB	50	5.163818	0.003
	70	3.503573	0.002
	142	4.459738	0.001
RVT	50	2.060866	0.009
	70	4.613933	0.001
	142	3.914494	0.001
RVB	50	1.45523	0.139
	70	4.282155	0.001
	142	5.425939	0.001

Supplementary Table 6. Kruskal-Wallis with FDR-corrected post-hoc Dunn's test for distances between naïve and infected at 50, 75, and 142 days post infection (DPI). LA, left atrium. RA, right atrium. LVT, left ventricle top. LVB, left ventricle bottom. RVT, right ventricle top. RVB, right ventricle bottom. FDR, false discovery rate. Data represents every possible pairwise combinations between N=15 mice per group and per position. Source data are provided as a Source Data file.

Heart section	Pair 1	Pair 2	FDR-corrected P value
LA	naïve_VS_infected_50DPI	naïve_VS_infected_75DPI	3.327069e-01
	naïve_VS_infected_50DPI	naïve_VS_infected_142DPI	2.678613e-18
	naïve_VS_infected_75DPI	naïve_VS_infected_142DPI	6.183185e-22
RA	naïve_VS_infected_50DPI	naïve_VS_infected_75DPI	0.7474485
	naïve_VS_infected_50DPI	naïve_VS_infected_142DPI	0.7727821
	naïve_VS_infected_75DPI	naïve_VS_infected_142DPI	0.6268322
LVT	naïve_VS_infected_50DPI	naïve_VS_infected_75DPI	0.007282892
	naïve_VS_infected_50DPI	naïve_VS_infected_142DPI	0.092750387
	naïve_VS_infected_75DPI	naïve_VS_infected_142DPI	0.244127579
LVB	naïve_VS_infected_50DPI	naïve_VS_infected_75DPI	2.233079e-09
	naïve_VS_infected_50DPI	naïve_VS_infected_142DPI	9.915717e-17
	naïve_VS_infected_75DPI	naïve_VS_infected_142DPI	1.686405e-02
RVT	naïve_VS_infected_50DPI	naïve_VS_infected_75DPI	0.05464850
	naïve_VS_infected_50DPI	naïve_VS_infected_142DPI	0.40073528
	naïve_VS_infected_75DPI	naïve_VS_infected_142DPI	0.01009132
RVB	naïve_VS_infected_50DPI	naïve_VS_infected_75DPI	0.2803490
	naïve_VS_infected_50DPI	naïve_VS_infected_142DPI	0.1506789
	naïve_VS_infected_75DPI	naïve_VS_infected_142DPI	0.9722301

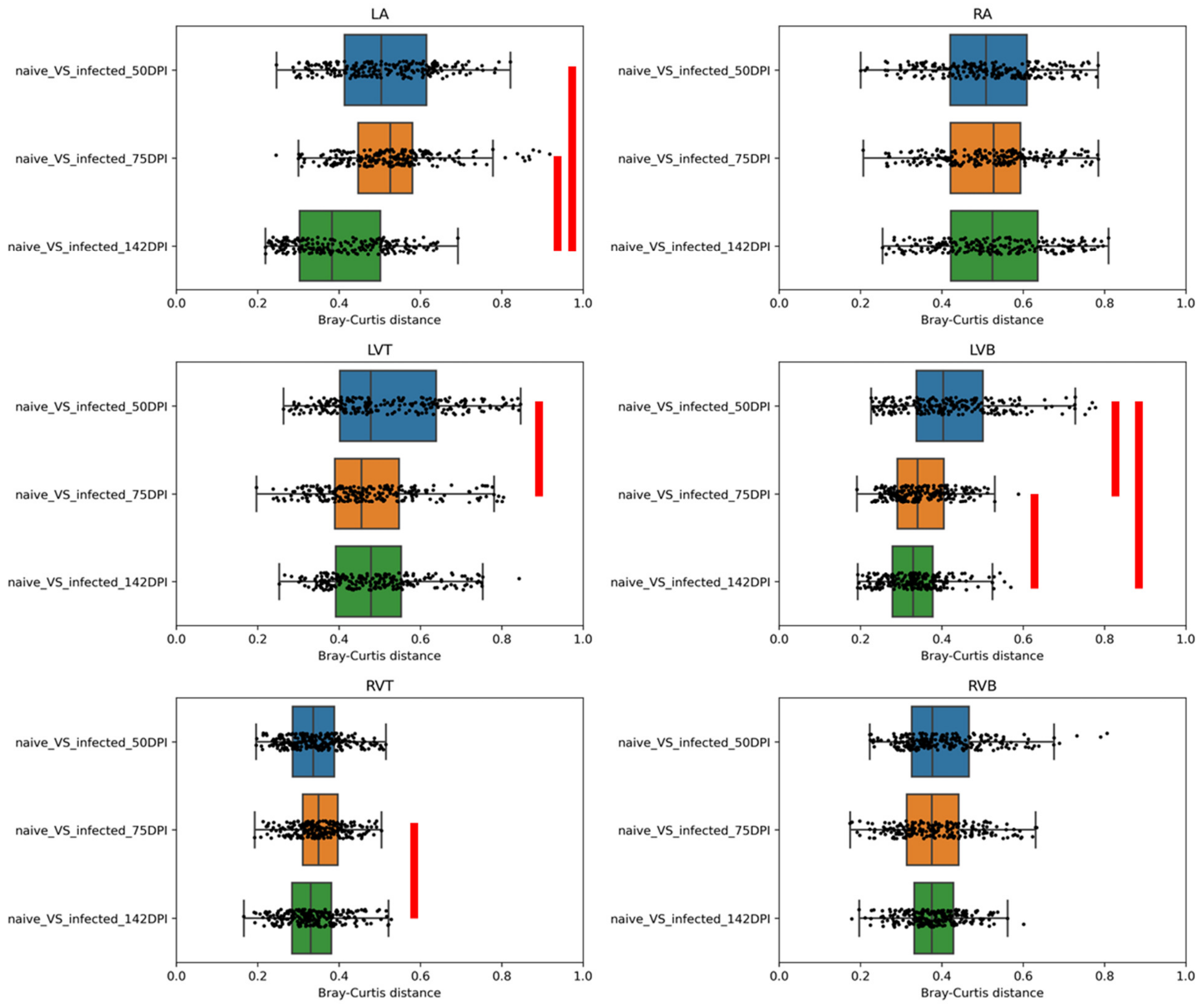

Supplementary Figure 8. Distance analysis boxplot between naïve and infected at 50, 75, and 142 days post infection (DPI). Each data point is the Bray-Curtis distance in terms of overall small molecule composition between every pair of samples. For example, in the boxplot marked naïve_vs_infected_50DPI, this would be the distance between every naïve sample and every infected sample, at 50 DPI, in all possible permutations. N=15 mice per group and per position. Boxplots represent median, upper and lower quartiles, with whiskers extending to show the rest of the distribution, except for points that are determined to be outliers by being beyond the interquartile range +/- 1.5 times the interquartile range. Red line, p-value <0.05 by Mann-Whitney U Test, two-sided, FDR-corrected. Exact p-values are in Supplementary Table 6. LA, left atrium. RA, right atrium. LVT, left ventricle top. LVB, left ventricle bottom. RVT, right ventricle top. RVB, right ventricle bottom. Source data are provided as a Source Data file.

Supplementary Table 7. Distance analysis between different treatment groups at 142 DPI. LA, left atrium. RA, right atrium. LVT, left ventricle top. LVB, left ventricle bottom. RVT, right ventricle top. RVB, right ventricle bottom. BNZ, benznidazole. N=15 mice per group and per position. Source data are provided as a Source Data file.

Heart section	Group 1	Group 2	pseudo-F	p-value
LA	naïve	BNZ	1.356518	0.219
	naïve	infected	1.095370	0.292
	naïve	combo	1.359726	0.228
	infected	BNZ	0.975107	0.379
	infected	combo	0.831580	0.459
	combo	BNZ	0.654794	0.647
RA	naïve	BNZ	1.247886	0.238
	naïve	infected	2.511789	0.041
	naïve	combo	1.288730	0.235
	infected	BNZ	0.929379	0.407
	infected	combo	1.144374	0.293
	combo	BNZ	0.346012	0.943
LVT	naïve	BNZ	1.484447	0.159
	naïve	infected	1.187019	0.292
	naïve	combo	1.461685	0.166
	infected	BNZ	1.051550	0.362
	infected	combo	1.214317	0.245
	combo	BNZ	2.061494	0.043
LVB	naïve	BNZ	2.715189	0.004
	naïve	infected	4.459738	0.002
	naïve	combo	3.321633	0.001
	infected	BNZ	1.295228	0.273
	infected	combo	1.753522	0.083
	combo	BNZ	1.869084	0.046
RVT	naïve	BNZ	3.087695	0.001
	naïve	infected	3.914494	0.001
	naïve	combo	2.366988	0.012
	infected	BNZ	2.117736	0.035
	infected	combo	2.610696	0.002
	combo	BNZ	1.013534	0.419
RVB	naïve	BNZ	4.910679	0.001
	naïve	infected	5.425939	0.001
	naïve	combo	3.276360	0.001
	infected	BNZ	2.552585	0.010
	infected	combo	4.760043	0.001
	combo	BNZ	2.368141	0.008

Supplementary Table 8. Kruskal-Wallis with FDR-corrected post-hoc Dunn's test for distances between different treatment groups at 142 DPI. LA, left atrium. RA, right atrium. LVT, left ventricle top. LVB, left ventricle bottom. RVT, right ventricle top. RVB, right ventricle bottom. BNZ, benznidazole. FDR, false discovery rate. Data represents every possible pairwise combinations between N=15 mice per group and per position. Source data are provided as a Source Data file.

Heart section	Pair 1	Pair 2	FDR-corrected p value
LA	naïve_VS_BNZ	naïve_VS_naïve	0.139782138
	naïve_VS_BNZ	naïve_VS_infected	0.160693030
	naïve_VS_naïve	naïve_VS_infected	0.776120010
	naïve_VS_BNZ	naïve_VS_combo	0.179862414
	naïve_VS_naïve	naïve_VS_combo	0.010418740
	naïve_VS_infected	naïve_VS_combo	0.005658156
RA	naïve_VS_BNZ	naïve_VS_naïve	0.1908288
	naïve_VS_BNZ	naïve_VS_infected	0.1936673
	naïve_VS_naïve	naïve_VS_infected	0.0262561
	naïve_VS_BNZ	naïve_VS_combo	0.4614456
	naïve_VS_naïve	naïve_VS_combo	0.1038900
	naïve_VS_infected	naïve_VS_combo	0.4266253
LVT	naïve_VS_BNZ	naïve_VS_naïve	0.88251583
	naïve_VS_BNZ	naïve_VS_infected	0.03130183
	naïve_VS_naïve	naïve_VS_infected	0.05686250
	naïve_VS_BNZ	naïve_VS_combo	0.01622882
	naïve_VS_naïve	naïve_VS_combo	0.03320798
	naïve_VS_infected	naïve_VS_combo	0.67229235
LVB	naïve_VS_BNZ	naïve_VS_naïve	9.456508e-10
	naïve_VS_BNZ	naïve_VS_infected	9.802618e-03
	naïve_VS_naïve	naïve_VS_infected	5.412709e-05
	naïve_VS_BNZ	naïve_VS_combo	8.212829e-03
	naïve_VS_naïve	naïve_VS_combo	5.778669e-05
	naïve_VS_infected	naïve_VS_combo	8.944717e-01
RVT	naïve_VS_BNZ	naïve_VS_naïve	1.523759e-04
	naïve_VS_BNZ	naïve_VS_infected	1.761728e-01
	naïve_VS_naïve	naïve_VS_infected	1.729419e-06
	naïve_VS_BNZ	naïve_VS_combo	1.527634e-01
	naïve_VS_naïve	naïve_VS_combo	6.861743e-03
	naïve_VS_infected	naïve_VS_combo	8.017866e-03
RVB	naïve_VS_BNZ	naïve_VS_naïve	8.521031e-01
	naïve_VS_BNZ	naïve_VS_infected	1.436145e-07
	naïve_VS_naïve	naïve_VS_infected	2.818214e-06
	naïve_VS_BNZ	naïve_VS_combo	7.745506e-01
	naïve_VS_naïve	naïve_VS_combo	8.225285e-01
	naïve_VS_infected	naïve_VS_combo	3.581962e-07

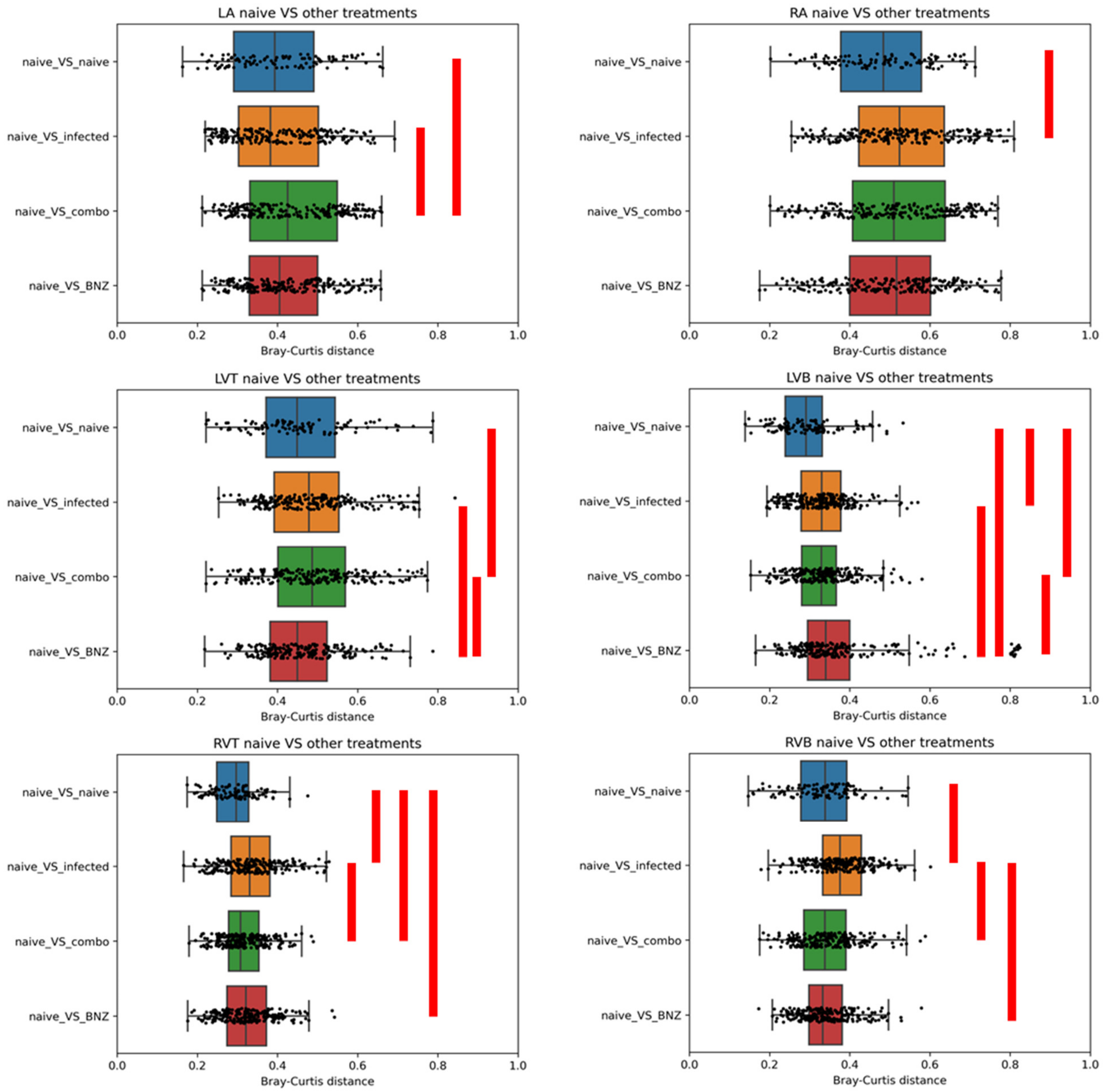

Supplementary Figure 9. Distance analysis boxplot between naïve groups and other experimental groups at 142 DPI. Each data point is the Bray-Curtis distance in terms of overall small molecule composition between every pair of samples in the listed groups. N=15 mice per group and per position. Red line, p-value <0.05 by Mann-Whitney U Test, two-sided, FDR-corrected. Boxplots represent median, upper and lower quartiles, with whiskers extending to show the rest of the distribution, except for points that are determined to be outliers by being beyond the interquartile range +/- 1.5 times the interquartile range. Exact p-values are in Supplementary Table 8. LA, left atrium. RA, right atrium. LVT, left ventricle top. LVB, left ventricle bottom. RVT, right ventricle top. RVB, right ventricle bottom. BNZ, benznidazole. Source data are provided as a Source Data file.

Supplementary Table 9. Kruskal-Wallis with FDR-corrected post-hoc Dunn's test of distances between infected no treatment group and other treatment groups at 142 DPI. LA, left atrium. RA, right atrium. LVT, left ventricle top. LVB, left ventricle bottom. RVT, right ventricle top. RVB, right ventricle bottom. BZN, benznidazole. FDR, false discovery rate. Data represents every possible pairwise combinations between N=15 mice per group and per position. Source data are provided as a Source Data file.

Heart section	Pair 1	Pair 2	FDR-corrected p value
LA	infected_VS_BNZ	infected_VS_infected	0.4411514
	infected_VS_BNZ	infected_VS_combo	0.1796762
	infected_VS_infected	infected_VS_combo	0.1328960
RA	infected_VS_BNZ	infected_VS_infected	0.7325454
	infected_VS_BNZ	infected_VS_combo	0.8818900
	infected_VS_infected	infected_VS_combo	0.9304309
LVT	infected_VS_BNZ	infected_VS_infected	0.11787664
	infected_VS_BNZ	infected_VS_combo	0.01237103
	infected_VS_infected	infected_VS_combo	0.59656541
LVB	infected_VS_BNZ	infected_VS_infected	0.01219156
	infected_VS_BNZ	infected_VS_combo	0.62243881
	infected_VS_infected	infected_VS_combo	0.01968717
RVT	infected_VS_BNZ	infected_VS_infected	0.3474791
	infected_VS_BNZ	infected_VS_combo	0.8204677
	infected_VS_infected	infected_VS_combo	0.5053970
RVB	infected_VS_BNZ	infected_VS_infected	1.123535e-01
	infected_VS_BNZ	infected_VS_combo	7.433810e-08
	infected_VS_infected	infected_VS_combo	6.365436e-03

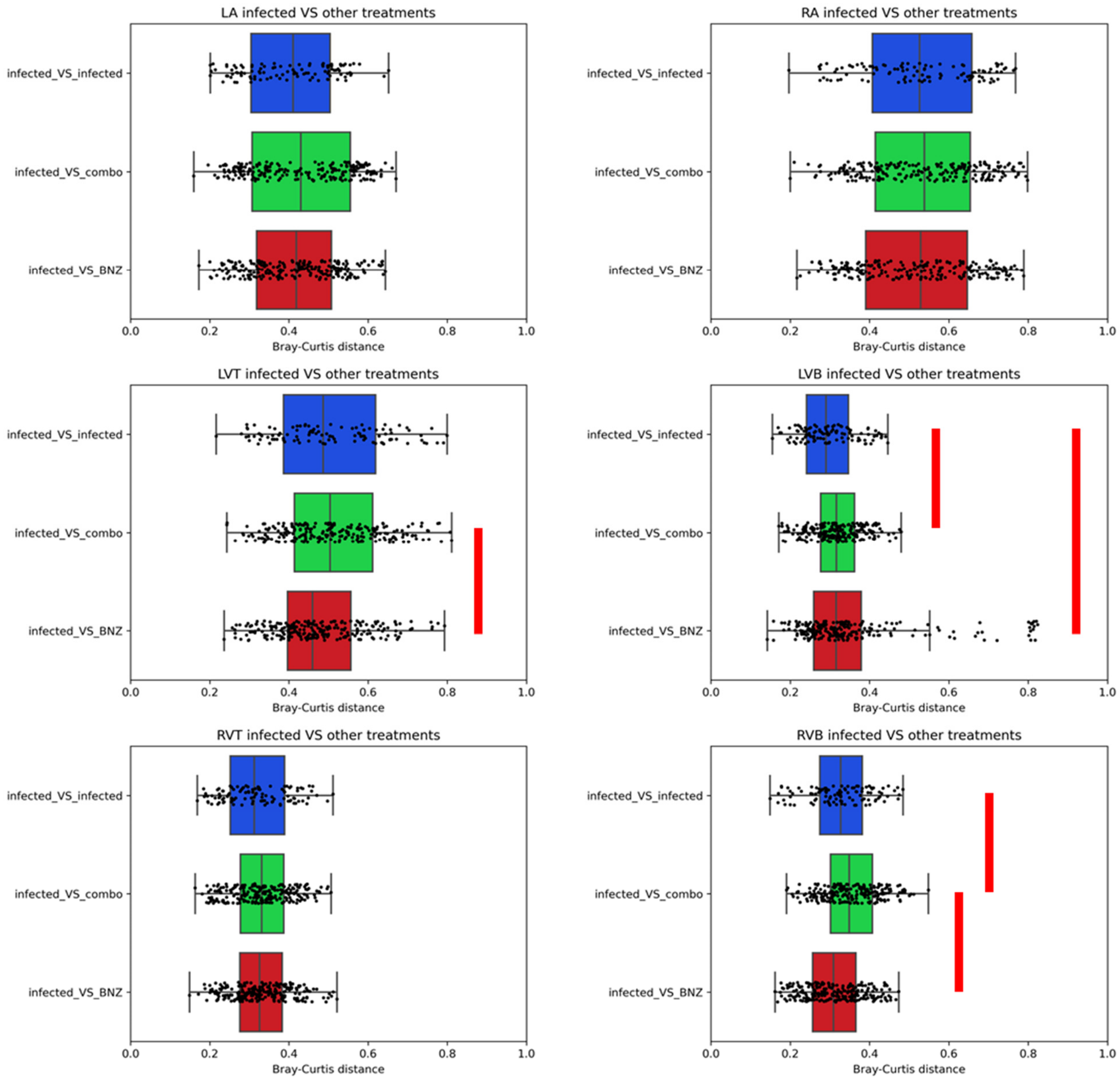

Supplementary Figure 10. Distance analysis boxplots between infected no treatment group and other treatment groups at 142 DPI. Each data point is the Bray-Curtis distance in terms of overall small molecule composition between every pair of samples. N=15 mice per group and per position. Red line, p-value <0.05 by Mann-Whitney U Test, two-sided, FDR-corrected. Boxplots represent median, upper and lower quartiles, with whiskers extending to show the rest of the distribution, except for points that are determined to be outliers by being beyond the interquartile range +/- 1.5 times the interquartile range. Exact p-values are in Supplementary Table 9. LA, left atrium. RA, right atrium. LVT, left ventricle top. LVB, left ventricle bottom. RVT, right ventricle top. RVB, right ventricle bottom. BNZ, benznidazole. Source data are provided as a Source Data file.

Supplementary Table 10. Distance analysis between different treatment groups at 142 DPI without high parasite burden post-BNZ treatment outlier mouse. LA, left atrium. RA, right atrium. LVT, left ventricle top. LVB, left ventricle bottom. RVT, right ventricle top. RVB, right ventricle bottom. BNZ, benznidazole. N=15 mice per group and per position. Source data are provided as a Source Data file.

Heart section	Group 1	Group 2	pseudo-F	p-value
LA	naïve	BNZ	1.325617	0.232
	naïve	infected	1.09537	0.31
	naïve	combo	1.359726	0.222
	infected	BNZ	0.894473	0.443
	infected	combo	0.83158	0.484
	combo	BNZ	0.769384	0.543
RA	naïve	BNZ	1.406805	0.186
	naïve	infected	2.511789	0.044
	naïve	combo	1.28873	0.214
	infected	BNZ	0.7668	0.522
	infected	combo	1.144374	0.279
	combo	BNZ	0.457876	0.852
LVT	naïve	BNZ	1.949067	0.054
	naïve	infected	1.187019	0.277
	naïve	combo	1.461685	0.173
	infected	BNZ	1.384779	0.182
	infected	combo	1.214317	0.265
	combo	BNZ	2.323148	0.026
LVB	naïve	BNZ	2.845178	0.001
	naïve	infected	4.459738	0.001
	naïve	combo	3.321633	0.001
	infected	BNZ	1.295049	0.279
	infected	combo	1.753522	0.078
	combo	BNZ	2.067337	0.031
RVT	naïve	BNZ	3.638358	0.002
	naïve	infected	3.914494	0.001
	naïve	combo	2.366988	0.008
	infected	BNZ	2.032209	0.046
	infected	combo	2.610696	0.002
	combo	BNZ	1.295884	0.224
RVB	naïve	BNZ	5.356061	0.001
	naïve	infected	5.425939	0.001
	naïve	combo	3.27636	0.004
	infected	BNZ	2.336337	0.018
	infected	combo	4.760043	0.001
	combo	BNZ	2.566525	0.004

Supplementary Table 11. Kruskal-Wallis with FDR-corrected post-hoc Dunn's test of distances between different treatment groups at 142 DPI without high parasite burden post-BNZ treatment outlier mouse. LA, left atrium. RA, right atrium. LVT, left ventricle top. LVB, left ventricle bottom. RVT, right ventricle top. RVB, right ventricle bottom. BNZ, benznidazole. FDR, false discovery rate. Data represents every possible pairwise combinations between N=15 mice per group and per position. Source data are provided as a Source Data file.

Heart section	Pair1	Pair2	FDR-corrected p value
LA	naïve_VS_BNZ	naïve_VS_naïve	0.295489565
	naïve_VS_BNZ	naïve_VS_infected	0.251628525
	naïve_VS_naïve	naïve_VS_infected	0.774607473
	naïve_VS_BNZ	naïve_VS_combo	0.095755447
	naïve_VS_naïve	naïve_VS_combo	0.010810319
	naïve_VS_infected	naïve_VS_combo	0.006009265
RA	naïve_VS_BNZ	naïve_VS_naïve	0.20069214
	naïve_VS_BNZ	naïve_VS_infected	0.20583003
	naïve_VS_naïve	naïve_VS_infected	0.02704533
	naïve_VS_BNZ	naïve_VS_combo	0.47119782
	naïve_VS_naïve	naïve_VS_combo	0.10686888
	naïve_VS_infected	naïve_VS_combo	0.42504515
LVT	naïve_VS_BNZ	naïve_VS_naïve	0.788488612
	naïve_VS_BNZ	naïve_VS_infected	0.011706694
	naïve_VS_naïve	naïve_VS_infected	0.057448034
	naïve_VS_BNZ	naïve_VS_combo	0.003267821
	naïve_VS_naïve	naïve_VS_combo	0.022433229
	naïve_VS_infected	naïve_VS_combo	0.672722645
LVB	naïve_VS_BNZ	naïve_VS_naïve	2.406726e-09
	naïve_VS_BNZ	naïve_VS_infected	1.397022e-02
	naïve_VS_naïve	naïve_VS_infected	5.705367e-05
	naïve_VS_BNZ	naïve_VS_combo	1.221490e-02
	naïve_VS_naïve	naïve_VS_combo	5.932090e-05
	naïve_VS_infected	naïve_VS_combo	9.001797e-01
RVT	naïve_VS_BNZ	naïve_VS_naïve	2.671548e-04
	naïve_VS_BNZ	naïve_VS_infected	1.818625e-01
	naïve_VS_naïve	naïve_VS_infected	1.764897e-06
	naïve_VS_BNZ	naïve_VS_combo	1.660460e-01
	naïve_VS_naïve	naïve_VS_combo	6.799045e-03
	naïve_VS_infected	naïve_VS_combo	8.235658e-03
RVB	naïve_VS_BNZ	naïve_VS_naïve	6.714309e-01
	naïve_VS_BNZ	naïve_VS_infected	1.777988e-06
	naïve_VS_naïve	naïve_VS_infected	2.842693e-06
	naïve_VS_BNZ	naïve_VS_combo	8.226742e-01
	naïve_VS_naïve	naïve_VS_combo	6.694273e-01
	naïve_VS_infected	naïve_VS_combo	6.575513e-07

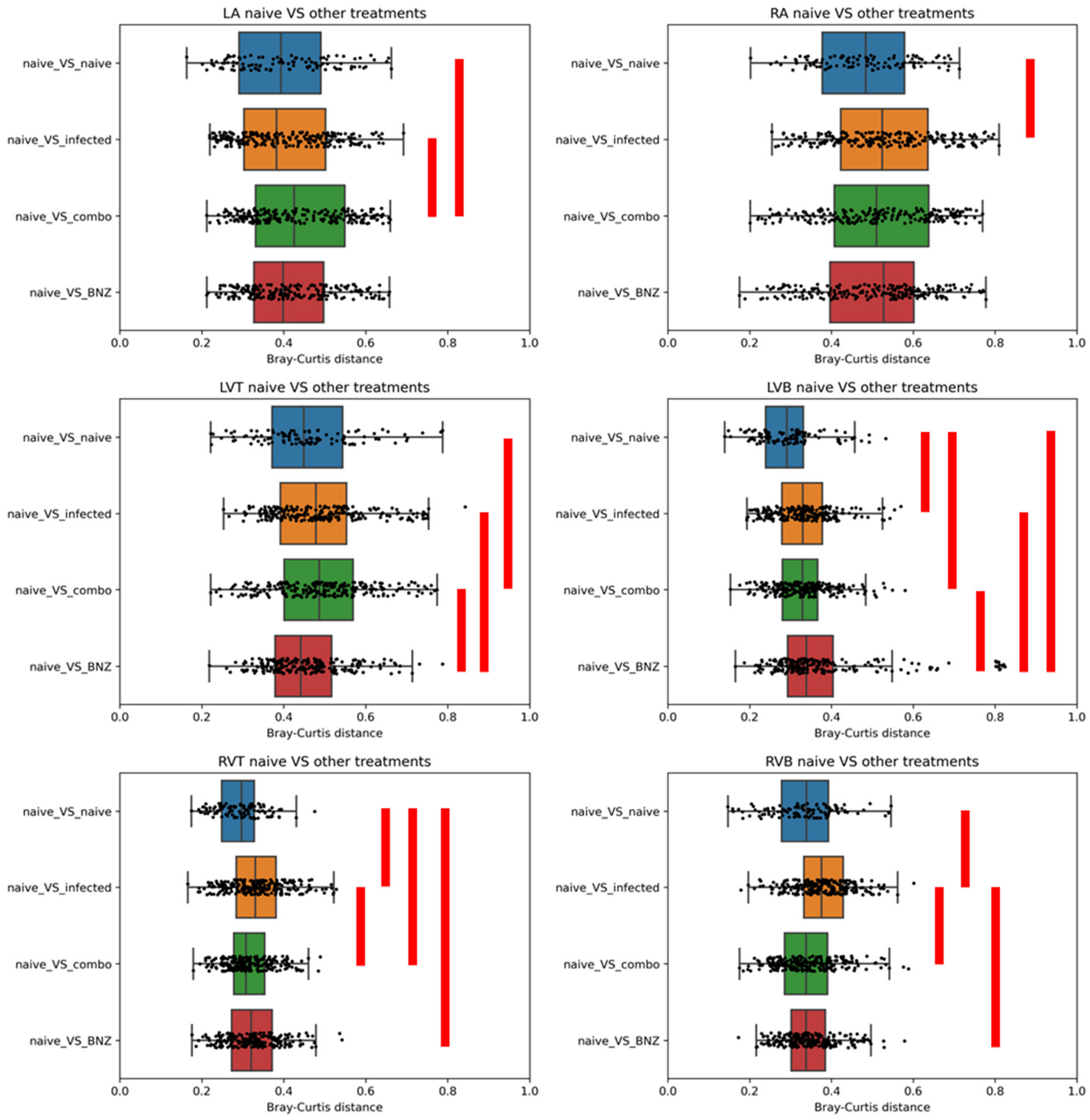

Supplementary Figure 11. Distance analysis boxplot between naïve groups and other experimental groups at 142DPI without high parasite burden post-BNZ treatment outlier mouse. Each data point is the Bray-Curtis distance in terms of overall small molecule composition between every pair of samples. N=15 mice per group and per position. Red line, p-value <0.05 by Mann-Whitney U Test, two-sided, FDR-corrected. Boxplots represent median, upper and lower quartiles, with whiskers extending to show the rest of the distribution, except for points that are determined to be outliers by being beyond the interquartile range +/- 1.5 times the interquartile range. Exact p-values are in Supplementary Table 11. LA, left atrium. RA, right atrium. LVT, left ventricle top. LVB, left ventricle bottom. RVT, right ventricle top. RVB, right ventricle bottom. BNZ, benznidazole. Source data are provided as a Source Data file.

Supplementary Table 12. Kruskal-Wallis with FDR-corrected post-hoc Dunn's test of distances between infected no treatment group and other treatment groups at 142 DPI without high parasite burden post-BNZ treatment outlier mouse. LA, left atrium. RA, right atrium. LVT, left ventricle top. LVB, left ventricle bottom. RVT, right ventricle top. RVB, right ventricle bottom. BNZ, benznidazole. FDR, false discovery rate. Data represents every possible pairwise combinations between N=15 mice per group and per position. Source data are provided as a Source Data file.

Heart section	Pair1	Pair2	FDR-corrected p value
LA	infected_VS_BNZ	infected_VS_infected	0.63264802
	infected_VS_BNZ	infected_VS_combo	0.09687013
	infected_VS_infected	infected_VS_combo	0.14182864
RA	infected_VS_BNZ	infected_VS_infected	0.7682509
	infected_VS_BNZ	infected_VS_combo	0.4544656
	infected_VS_infected	infected_VS_combo	0.6159846
LVT	infected_VS_BNZ	infected_VS_infected	0.060225950
	infected_VS_BNZ	infected_VS_combo	0.003792884
	infected_VS_infected	infected_VS_combo	0.587838689
LVB	infected_VS_BNZ	infected_VS_infected	0.02481829
	infected_VS_BNZ	infected_VS_combo	0.91364429
	infected_VS_infected	infected_VS_combo	0.03603772
RVT	infected_VS_BNZ	infected_VS_infected	0.5520473
	infected_VS_BNZ	infected_VS_combo	0.4810231
	infected_VS_infected	infected_VS_combo	0.4777285
RVB	infected_VS_BNZ	infected_VS_infected	4.311688e-02
	infected_VS_BNZ	infected_VS_combo	4.854801e-09
	infected_VS_infected	infected_VS_combo	6.522770e-03

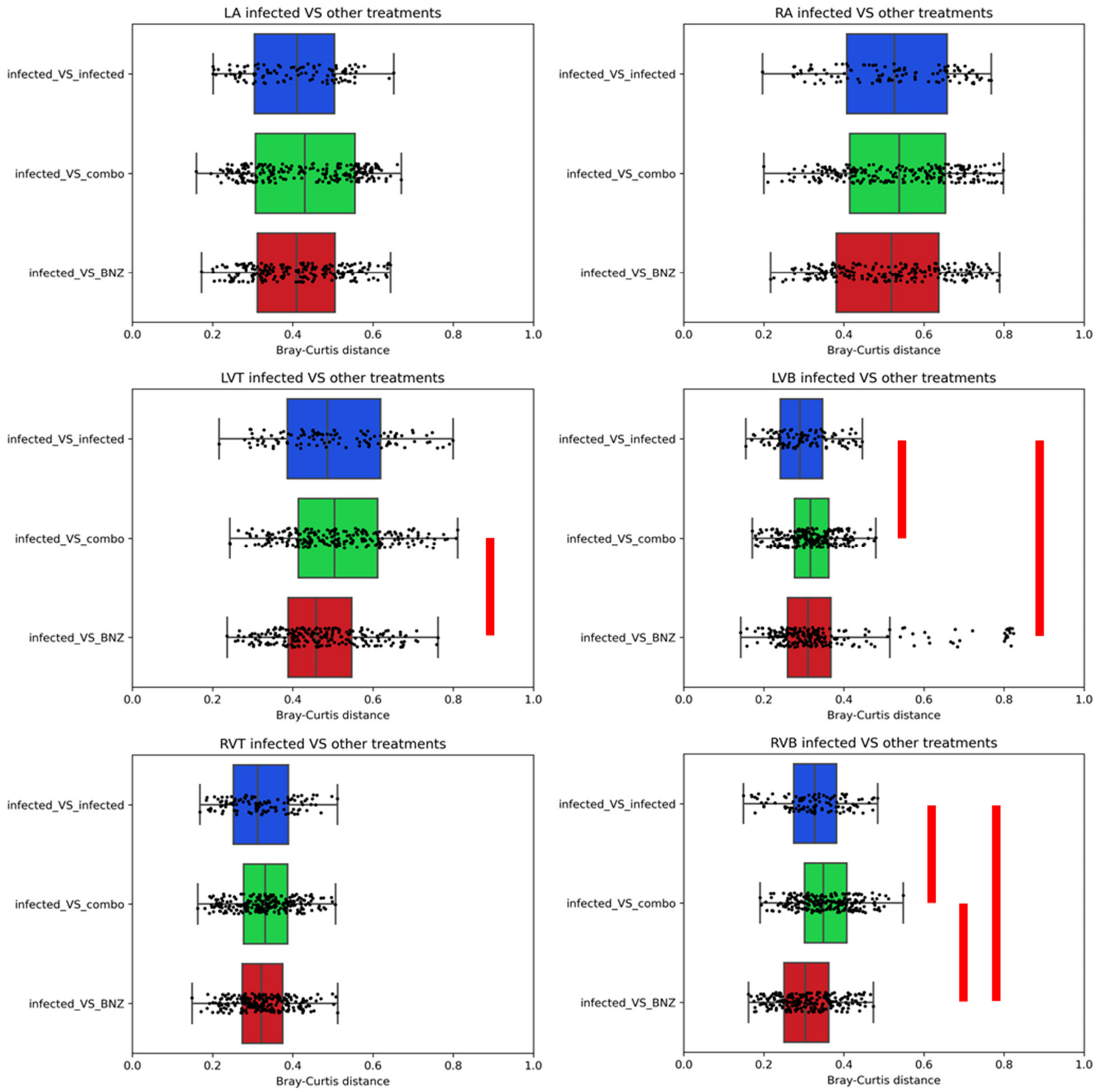

Supplementary Figure 12. Distance analysis boxplots between infected no treatment group and other treatment groups at 142DPI without high parasite burden post-BNZ treatment outlier mouse. Each data point is the Bray-Curtis distance in terms of overall small molecule composition between every pair of samples. N=15 mice per group and per position. Red line, p-value <0.05 by Mann-Whitney U Test, two-sided, FDR-corrected. Boxplots represent median, upper and lower quartiles, with whiskers extending to show the rest of the distribution, except for points that are determined to be outliers by being beyond the interquartile range +/- 1.5 times the interquartile range. Exact p-values are in Supplementary Table 12. LA, left atrium. RA, right atrium. LVT, left ventricle top. LVB, left ventricle bottom. RVT, right ventricle top. RVB, right ventricle bottom. BNZ, benznidazole. Source data are provided as a Source Data file.

Supplementary Note 1. Confirmation of incomplete metabolic restoration by BNZ in an independent *T. cruzi* infection system.

Methods – *in vivo* experimentation: 5-week-old male Swiss Webster mice (Charles River) were infected by intraperitoneal injection with 500,000 Sylvio X10/4 parasites or mock-injected (uninfected controls). Ten weeks post-infection, mice were treated for 14 days with 100 mg/kg benznidazole (BNZ) by intraperitoneal injection or left untreated. Mice were euthanized 4 weeks post-treatment (112 days post-infection). Hearts were collected and either placed in RNAlater (for RNA-seq) or snap-frozen in liquid nitrogen (for LC-MS).

Methods – RNA analysis: RNA was extracted using the Zymo ZRDuet DNA/RNA Miniprep Plus kit. 4 µg RNA was shipped in RNA stabilization tubes to GENEWIZ®, who performed cDNA library synthesis and high-throughput Illumina sequencing. Paired-end sequence data was quality filtered using AdapterRemoval (v2) to remove reads with uncalled bases (‘N’), residual adapter sequences, and trimmed to remove bases with low quality (q < 30) [1]. Transcript quantification was performed using Salmon (v1.1.0), with the ENSEMBL mouse transcriptome (GRCm38, build p6) as reference [2]. The resulting quantification data was imported into R (‘tximport') [3], followed by gene expression analysis using DESeq2 [4]. N=5/group.

Methods – LC-MS analysis: Small molecule extraction and data acquisition were as described in the main manuscript Methods, except that sample injection volume was 20 µL. Data was processed in MZmine as in **Supplementary Table 4**, except for the following parameters: MS1 noise level, 3E6; deconvolution method: Wavelets (ADAP), with signal to noise threshold: 10, signal to noise estimator: intensity window signal to noise, minimum feature height: 3E6, coefficient/area threshold: 110, peak duration range: 0.03-1.5 min, RT wavelet range: 0-0.1; deisotoping: representative isotope was most intense; filtering: retention time 0.2-7.2 min; gap-filling: intensity tolerance, 10%; *m/z* tolerance, 10 ppm; retention time tolerance: 0.5 min. Batch correction was performed using WaveICA 2.0 [5]. All parameters were set to default, except the following: alpha, 0; cutoff, 0.1; k, 20. Principal coordinate and PERMANOVA analysis was performed using QIIME2 [6]. N=5/group (BNZ, vehicle) and N=6/group (uninfected).

Methods – data availability: RNA-seq data has been deposited in SRA, bioproject accession PRJNA670449 [https://www.ncbi.nlm.nih.gov/bioproject/PRJNA670449/]. Small molecule analysis data has been deposited in MassIVE, accession number MSV000092090 [doi:10.25345/C5C824Q8C]. Source data are provided as a Source Data file.

See Supplementary Figures 13-16, below, for LC-MS and RNA-seq data.

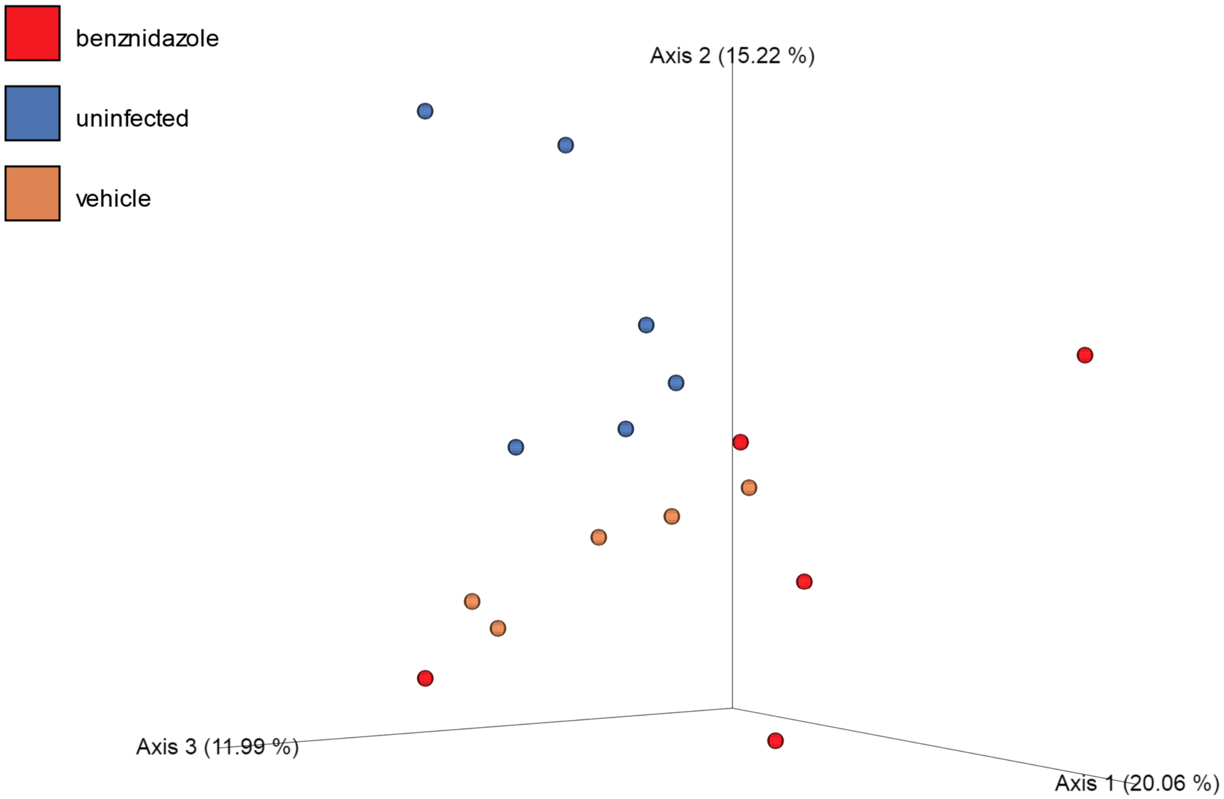

Supplementary Figure 13. Lack of small molecule restoration in BNZ-treated mice. Pairwise PERMANOVA BNZ to uninfected, q-value=0.036, pseudo-F=1.794449. Pairwise PERMANOVA BNZ to vehicle, q-value=0.799, pseudo-F=0.757399. N=5/group (BNZ, vehicle) and N=6/group (uninfected). Source data are provided as a Source Data file.

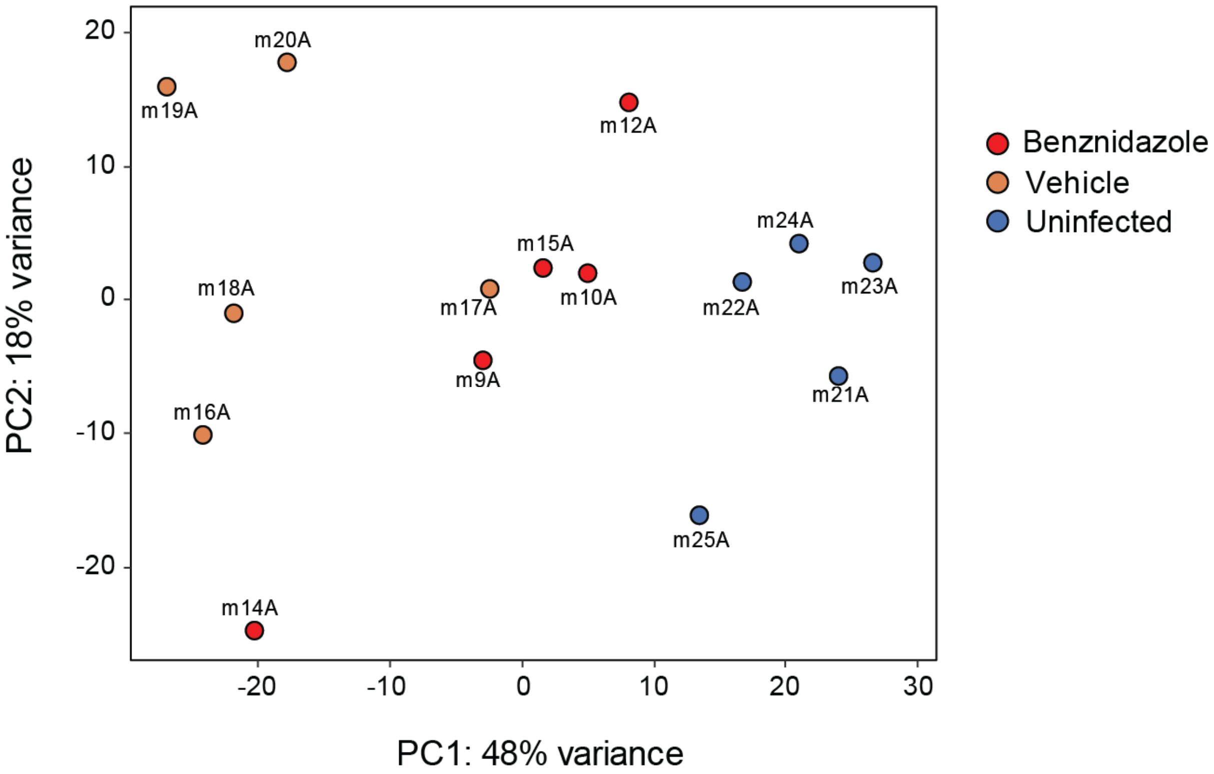

Supplementary Figure 14. Incomplete restoration of gene expression in BNZ-treated mice by PCA analysis. Principal component analysis of regularized logarithm transformed (‘rlog') gene counts with treatment-independent estimation of dispersion. N=5/group. PC, principal component. Source data are provided as a Source Data file.

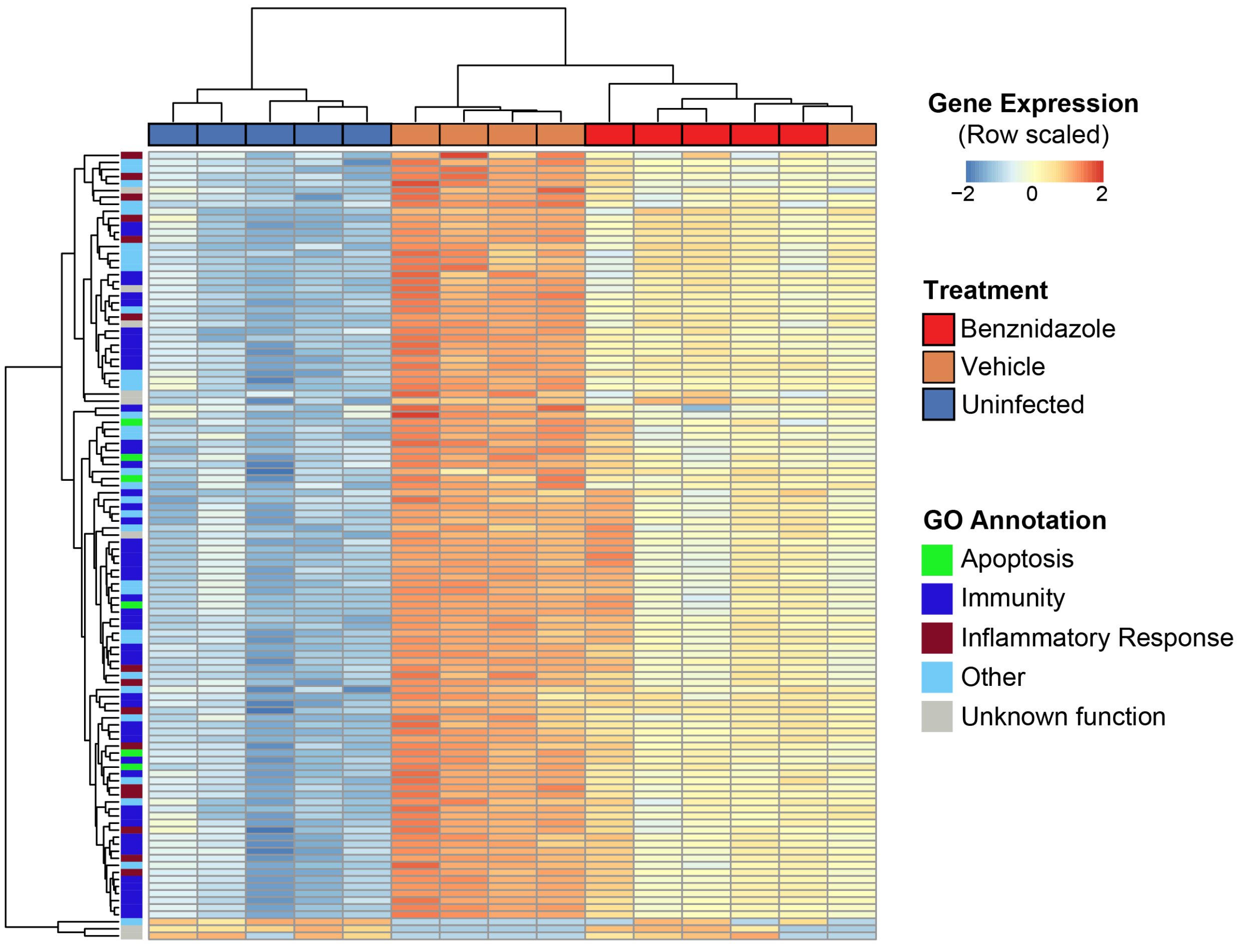

Supplementary Figure 15. Only partial restoration of transcripts in BNZ-treated mice. Likelihood ratio tests with treatment as the predictor variable identified a total of 112 differentially expressed genes (Log-fold change: ±2, FDR-adjusted P < 0.05). Of these, 51 genes are associated with immunity, and 15 genes are associated with inflammatory response processes. Heatmap generated from log2-transformed (pseudo-count of 1), centered and scaled (row mean = 0, row s.d. = 1), gene counts. Rows represent differentially expressed genes annotated by Gene Ontology (Biological process). Columns represent mouse samples annotated by treatment group. N=5/group. Source data are provided as a Source Data file.

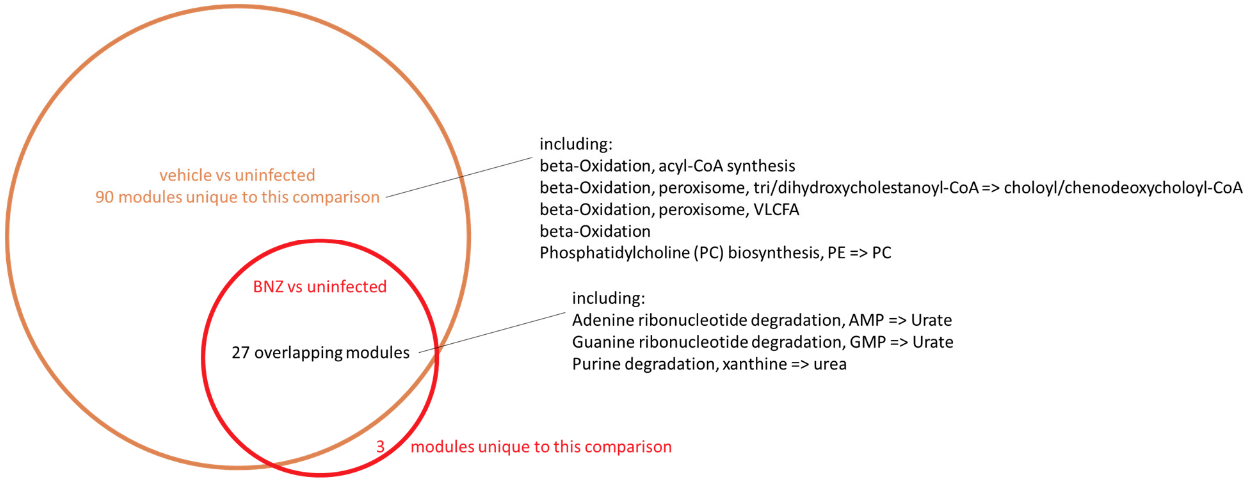

Supplementary Figure 16. Impact of BNZ treatment on infection-perturbed metabolic modules. Gene count data was aggregated at the metabolic module-level. Likelihood ratio tests with treatment as the predictor variable were used to identify differential metabolic modules (FDR-adjusted P < 0.05), followed by pairwise comparisons (vehicle-uninfected, and BNZ-uninfected) using Wald tests (FDR-adjusted P < 0.05). Strikingly, most of the differential modules between BNZ and uninfected were also differential between vehicle and uninfected, supporting the occurrence of infection-induced metabolic changes that fail to be restored by BNZ treatment. Overlapping modules between vehicle vs uninfected and BNZ vs uninfected include multiple modules related to purine metabolism, matching with our small molecule analysis data showing that it is harder to restore purine metabolism. On the other hand, beta-oxidation modules were only differential for the comparison between vehicle vs uninfected and not BNZ vs uninfected, concurring with our small molecule analysis showing easier restoration of lipid metabolites. PE, phosphatidylethanolamine. VLCFA, very long chain fatty acids. N=5/group. Source data is in Supplementary Table 1.

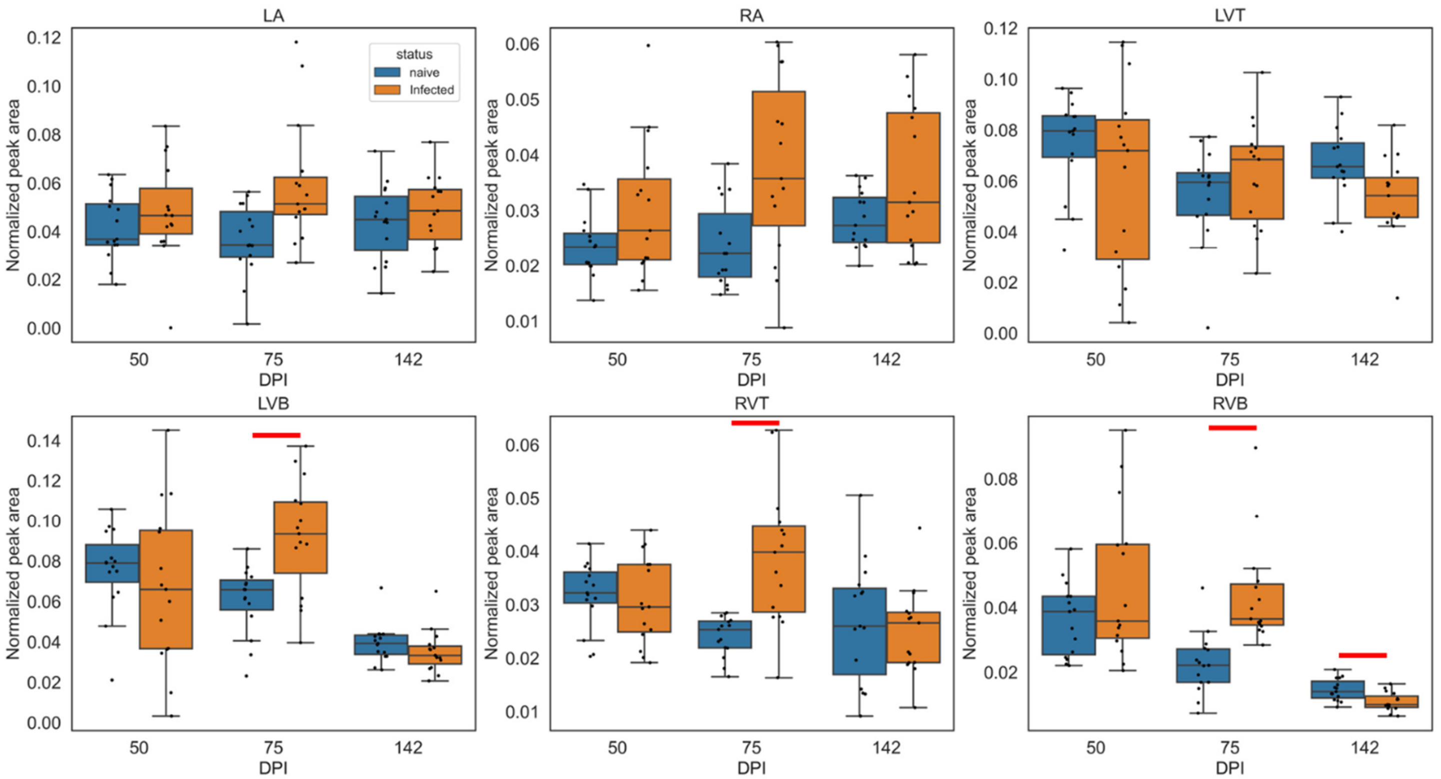

Supplementary Figure 17. Impact of infection duration on glycerophosphocholines in *m/z* range 400 to 500. Data represents summed peak areas for all glycerophosphocholines in *m/z* range 400 to 500, identified as described in Methods. DPI, days post-infection. LA, left atrium. RA, right atrium. LVT, left ventricle top. LVB, left ventricle bottom. RVT, right ventricle top. RVB, right ventricle bottom. Red line, p-value < 0.05 by Mann-Whitney U test, two-sided, FDR-corrected. Boxplots represent median, upper and lower quartiles, with whiskers extending to show the rest of the distribution, except for points that are determined to be outliers by being beyond the interquartile range +/- 1.5 times the interquartile range. N=15 mice per group and per position. Source data are provided as a Source Data file.

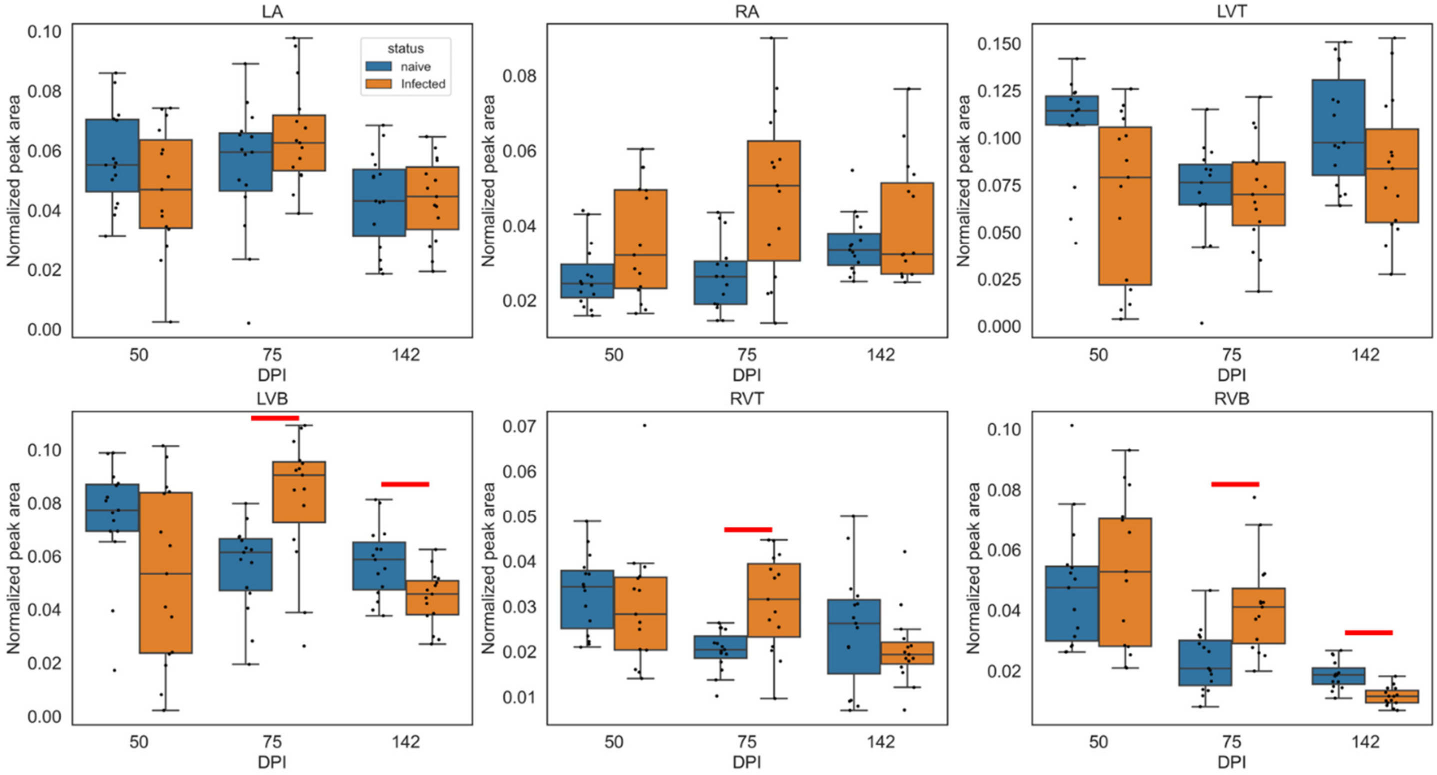

Supplementary Figure 18. Impact of infection duration on glycerophosphocholines in *m/z* range 500 to 600. Data represents summed peak areas for all glycerophosphocholines in *m/z* range 500 to 600. DPI, days post-infection. LA, left atrium. RA, right atrium. LVT, left ventricle top. LVB, left ventricle bottom. RVT, right ventricle top. RVB, right ventricle bottom. Red line, p-value < 0.05 by Mann-Whitney U test, two-sided, FDR-corrected. Boxplots represent median, upper and lower quartiles, with whiskers extending to show the rest of the distribution, except for points that are determined to be outliers by being beyond the interquartile range +/- 1.5 times the interquartile range. N=15 mice per group and per position. Source data are provided as a Source Data file.

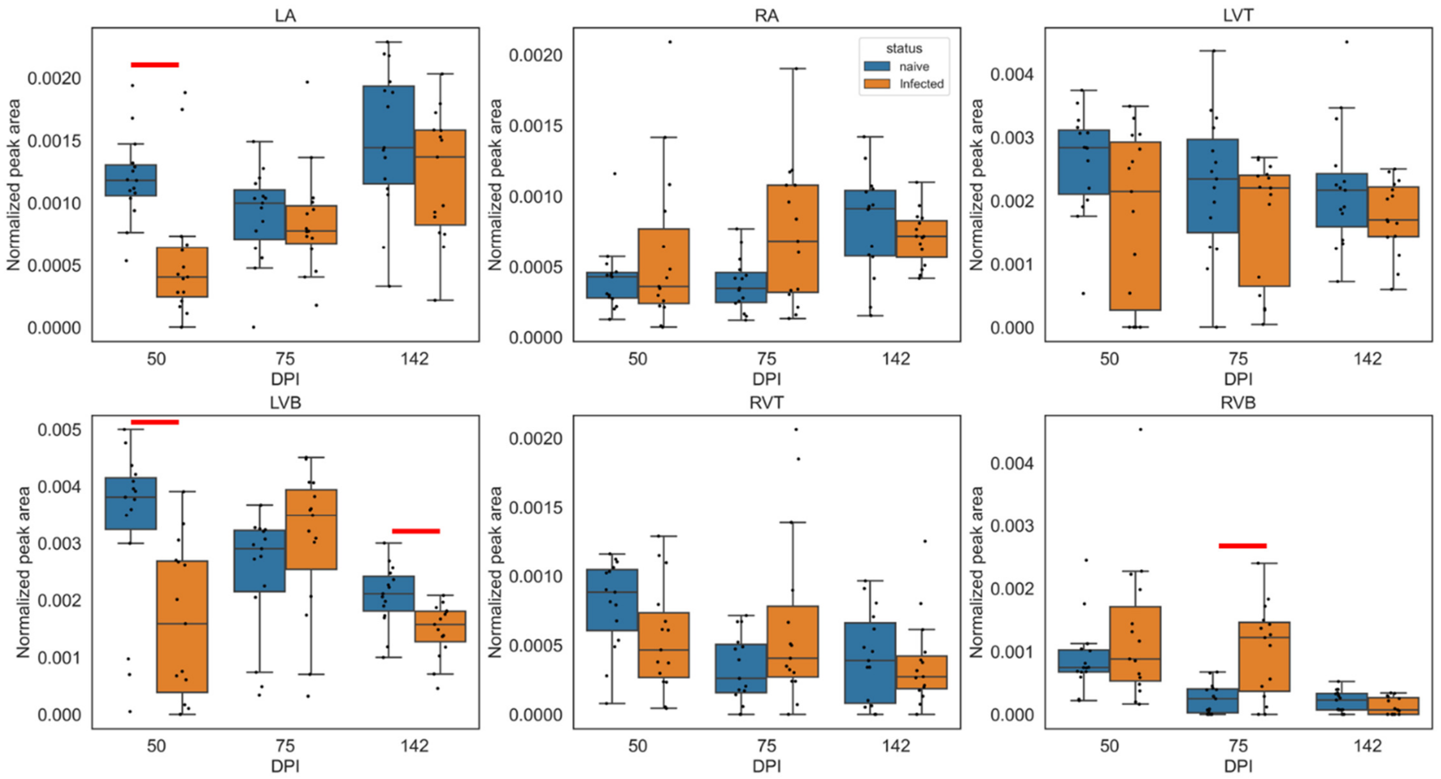

Supplementary Figure 19. Impact of infection duration on glycerophosphocholines in *m/z* range 600 to 700. Data represents summed peak areas for all glycerophosphocholines in *m/z* range 600 to 700, identified as described in Methods. DPI, days post-infection. LA, left atrium. RA, right atrium. LVT, left ventricle top. LVB, left ventricle bottom. RVT, right ventricle top. RVB, right ventricle bottom. Red line, p-value < 0.05 by Mann-Whitney U test, two-sided, FDR-corrected. Boxplots represent median, upper and lower quartiles, with whiskers extending to show the rest of the distribution, except for points that are determined to be outliers by being beyond the interquartile range +/- 1.5 times the interquartile range. N=15 mice per group and per position. Source data are provided as a Source Data file.

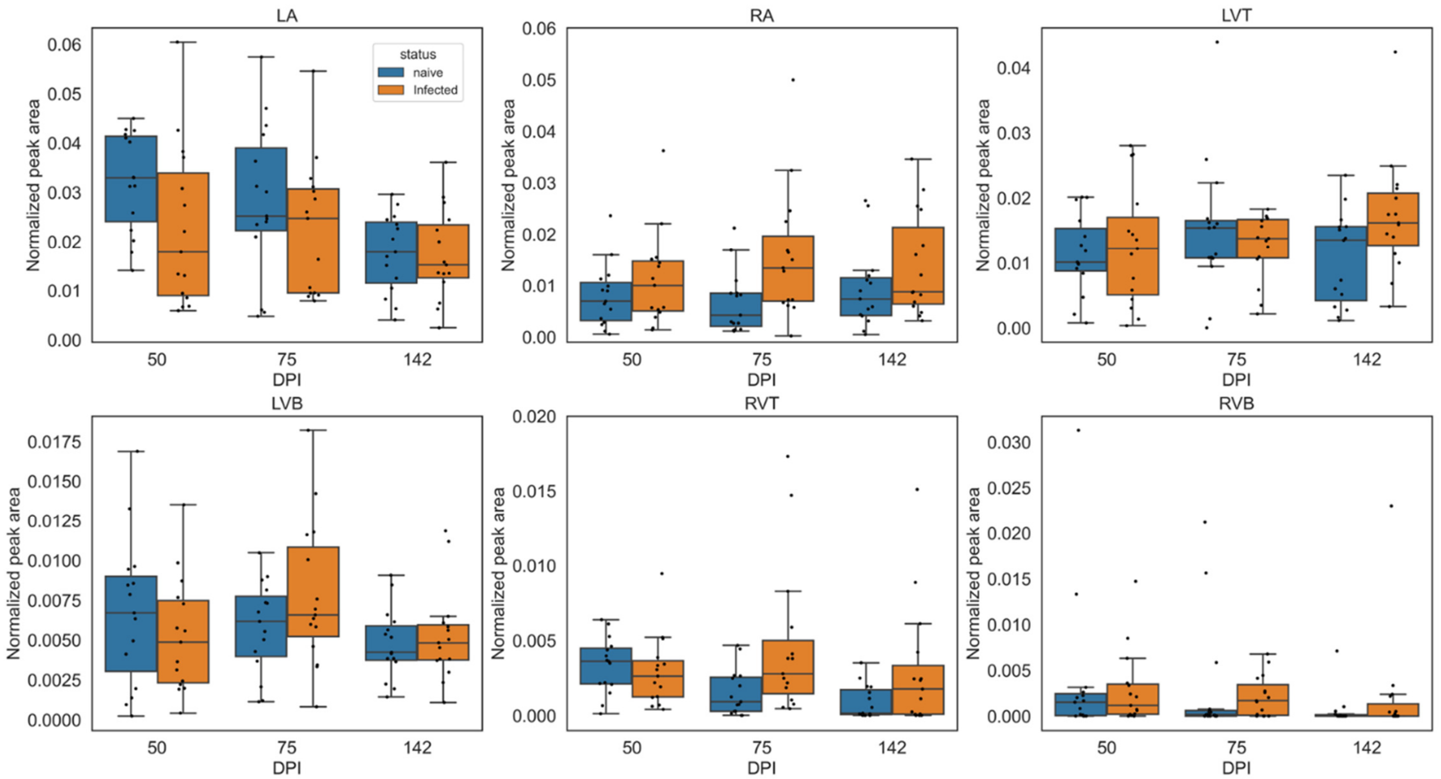

Supplementary Figure 20. Impact of infection duration on glycerophosphocholines in *m/z* range 700 to 800. Data represents summed peak areas for all glycerophosphocholines in *m/z* range 700 to 800, identified as described in Methods. DPI, days post-infection. LA, left atrium. RA, right atrium. LVT, left ventricle top. LVB, left ventricle bottom. RVT, right ventricle top. RVB, right ventricle bottom. Red line, p-value < 0.05 by Mann-Whitney U test, two-sided, FDR-corrected. Boxplots represent median, upper and lower quartiles, with whiskers extending to show the rest of the distribution, except for points that are determined to be outliers by being beyond the interquartile range +/- 1.5 times the interquartile range. N=15 mice per group and per position. Source data are provided as a Source Data file.

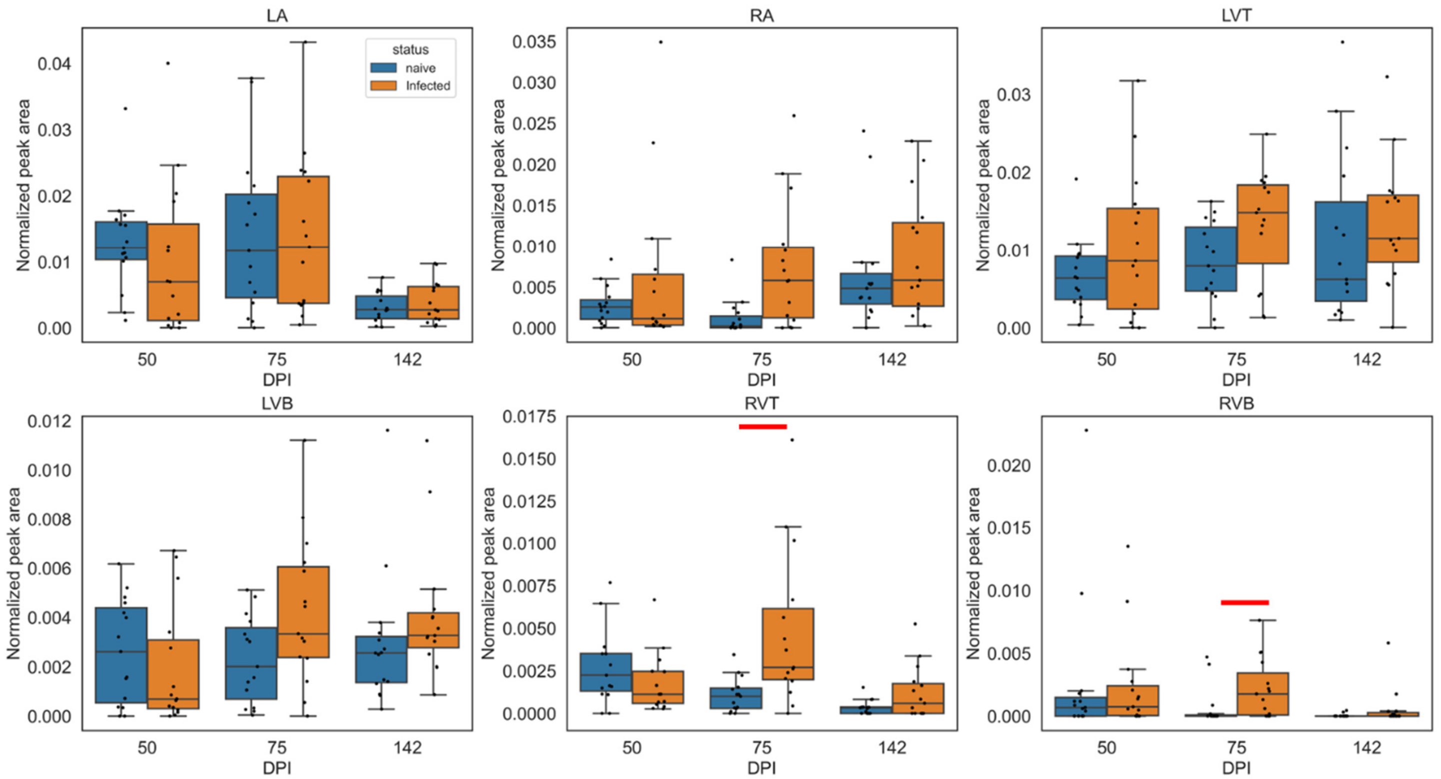

Supplementary Figure 21. Impact of infection duration on glycerophosphocholines in *m/z* range 800 to 900. Data represents summed peak areas for all glycerophosphocholines in *m/z* range 800 to 900, identified as described in Methods. DPI, days post-infection. LA, left atrium. RA, right atrium. LVT, left ventricle top. LVB, left ventricle bottom. RVT, right ventricle top. RVB, right ventricle bottom. Red line, p-value < 0.05 by Mann-Whitney U test, two-sided, FDR-corrected. Boxplots represent median, upper and lower quartiles, with whiskers extending to show the rest of the distribution, except for points that are determined to be outliers by being beyond the interquartile range +/- 1.5 times the interquartile range. N=15 mice per group and per position. Source data are provided as a Source Data file.

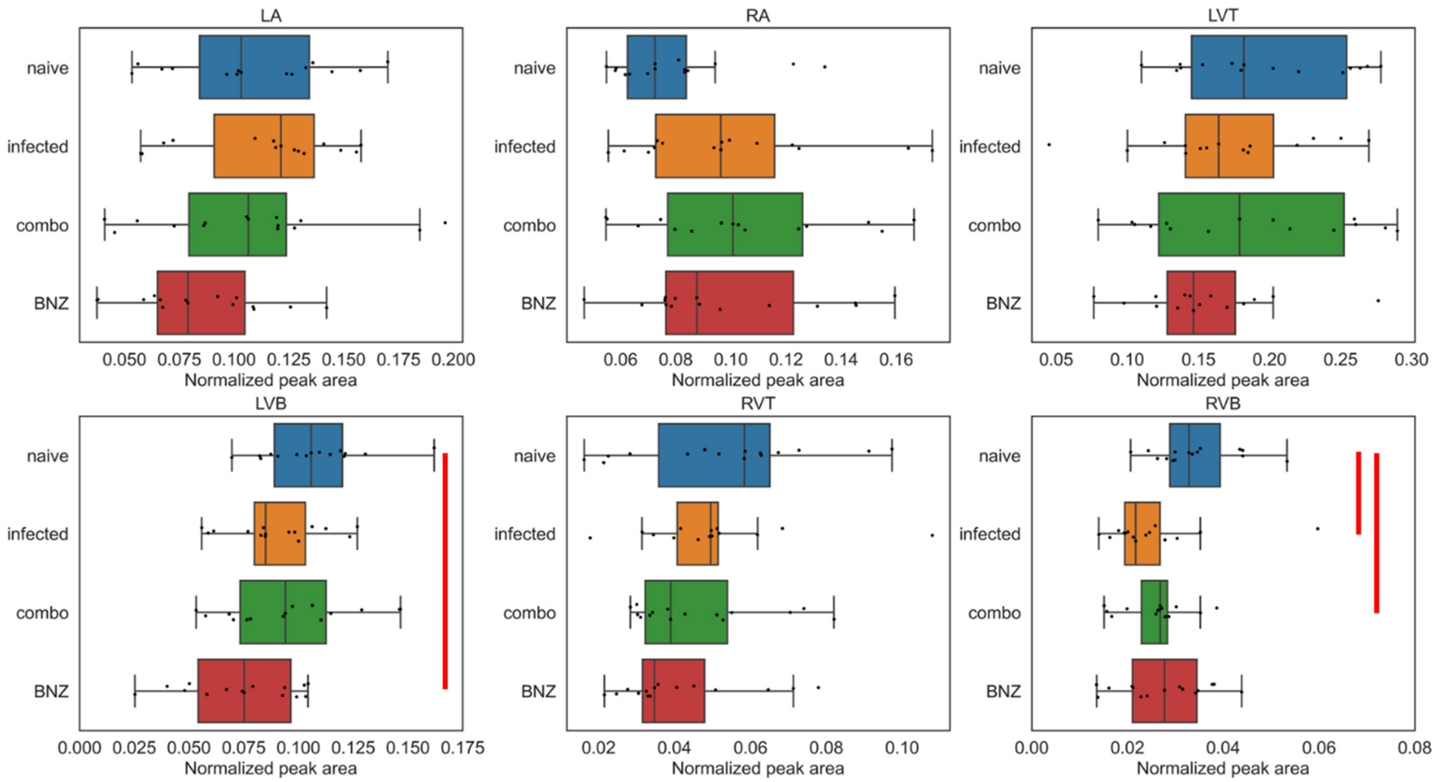

Supplementary Figure 22. Impact of treatment on total glycerophosphocholines. Data represents summed peak areas for all glycerophosphocholines, identified as described in Methods. Red line, p-value < 0.05 by Mann-Whitney U test, two-sided, FDR-corrected. Boxplots represent median, upper and lower quartiles, with whiskers extending to show the rest of the distribution, except for points that are determined to be outliers by being beyond the interquartile range +/- 1.5 times the interquartile range. LA, left atrium. RA, right atrium. LVT, left ventricle top. LVB, left ventricle bottom. RVT, right ventricle top. RVB, right ventricle bottom. BNZ, benznidazole. N=15 mice per group and per position. Source data are provided as a Source Data file.

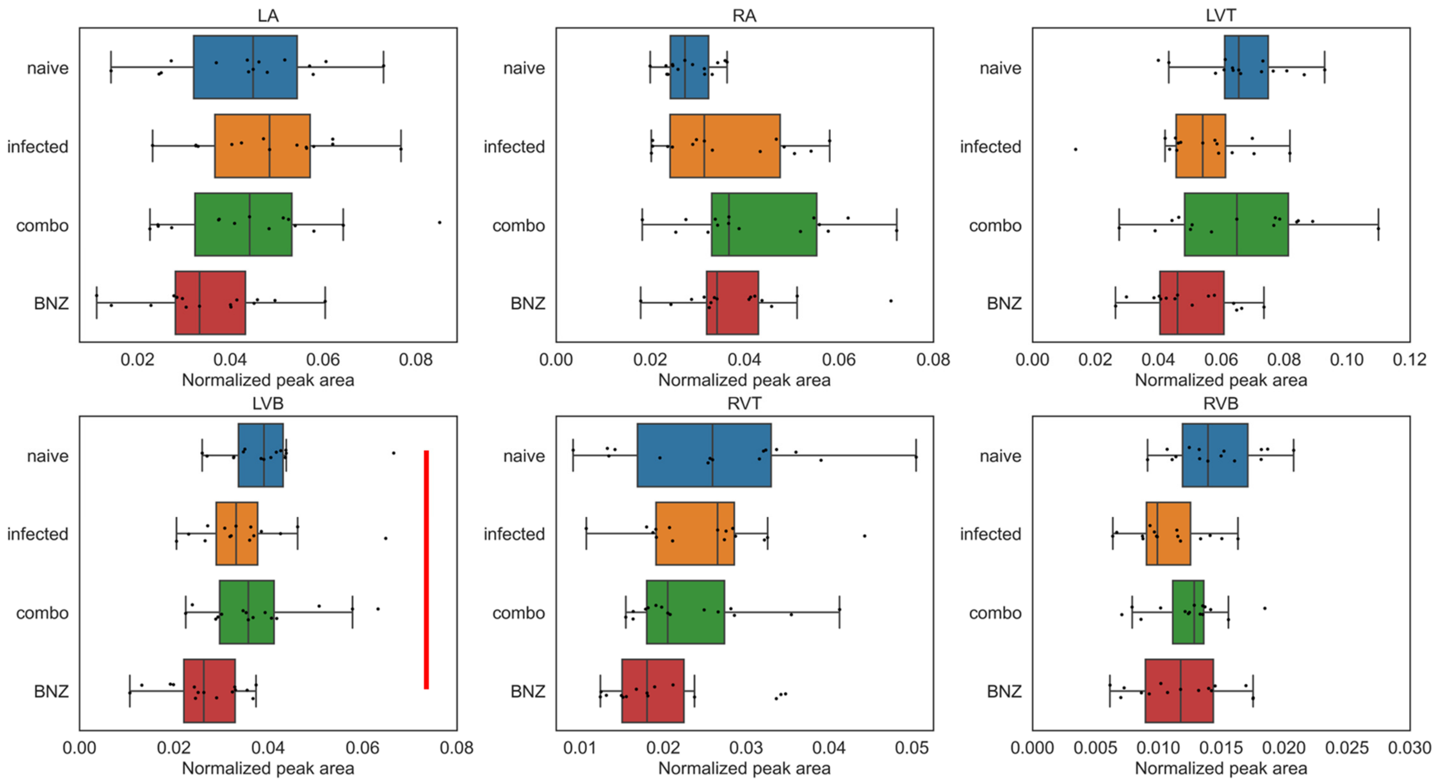

Supplementary Figure 23. Impact of treatment on glycerophosphocholines in *m/z* range 400 to 500. Data represents summed peak areas for glycerophosphocholines in *m/z* range 400 to 500, identified as described in Methods. Red line, p-value < 0.05 by Mann-Whitney U test, two-sided, FDR-corrected. Boxplots represent median, upper and lower quartiles, with whiskers extending to show the rest of the distribution, except for points that are determined to be outliers by being beyond the interquartile range +/- 1.5 times the interquartile range. LA, left atrium. RA, right atrium. LVT, left ventricle top. LVB, left ventricle bottom. RVT, right ventricle top. RVB, right ventricle bottom. BNZ, benznidazole. N=15 mice per group and per position. Source data are provided as a Source Data file.

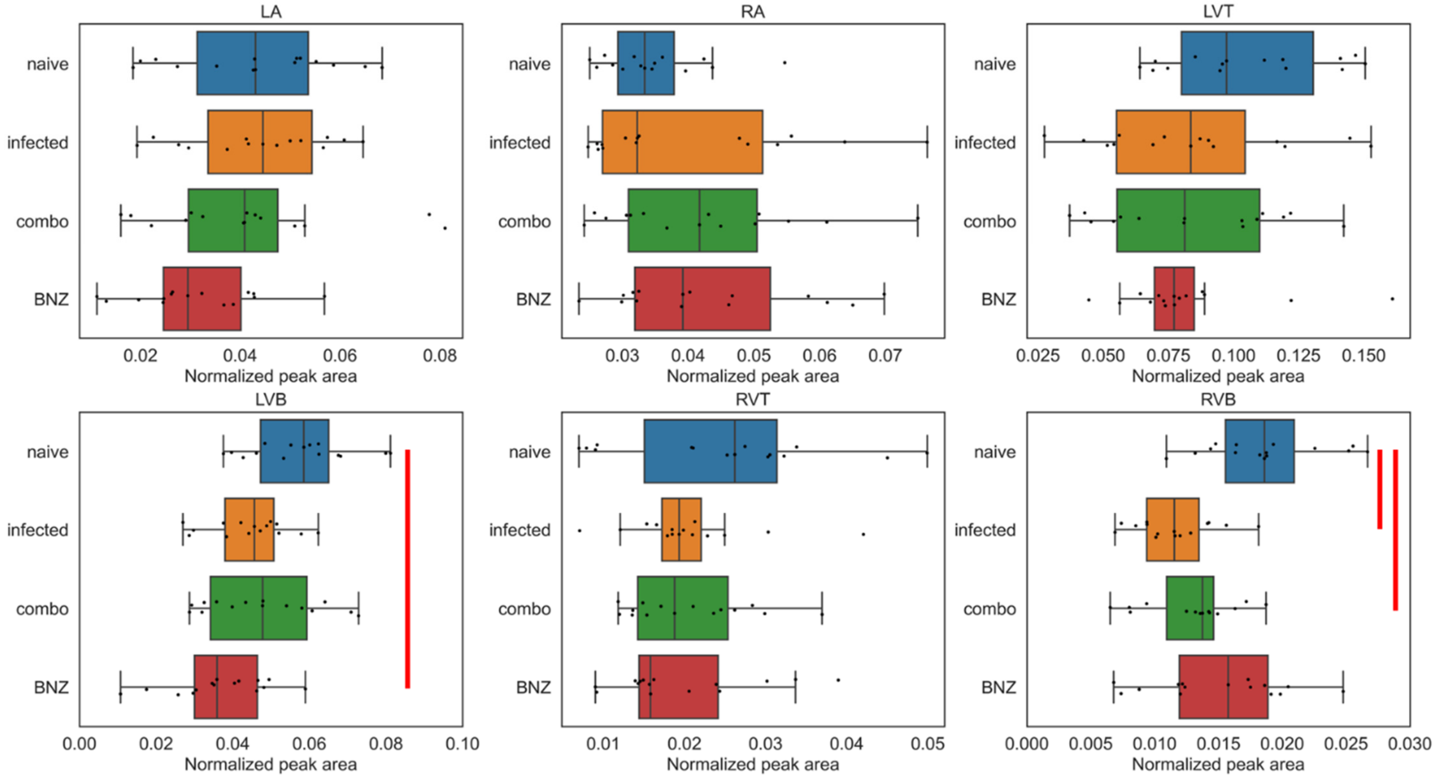

Supplementary Figure 24. Impact of treatment on glycerophosphocholines in *m/z* range 500 to 600. Data represents summed peak areas for glycerophosphocholines in *m/z* range 500 to 600, identified as described in Methods. Red line, p-value < 0.05 by Mann-Whitney U test, two-sided, FDR-corrected. Boxplots represent median, upper and lower quartiles, with whiskers extending to show the rest of the distribution, except for points that are determined to be outliers by being beyond the interquartile range +/- 1.5 times the interquartile range. LA, left atrium. RA, right atrium. LVT, left ventricle top. LVB, left ventricle bottom. RVT, right ventricle top. RVB, right ventricle bottom. BNZ, benznidazole. N=15 mice per group and per position. Source data are provided as a Source Data file.

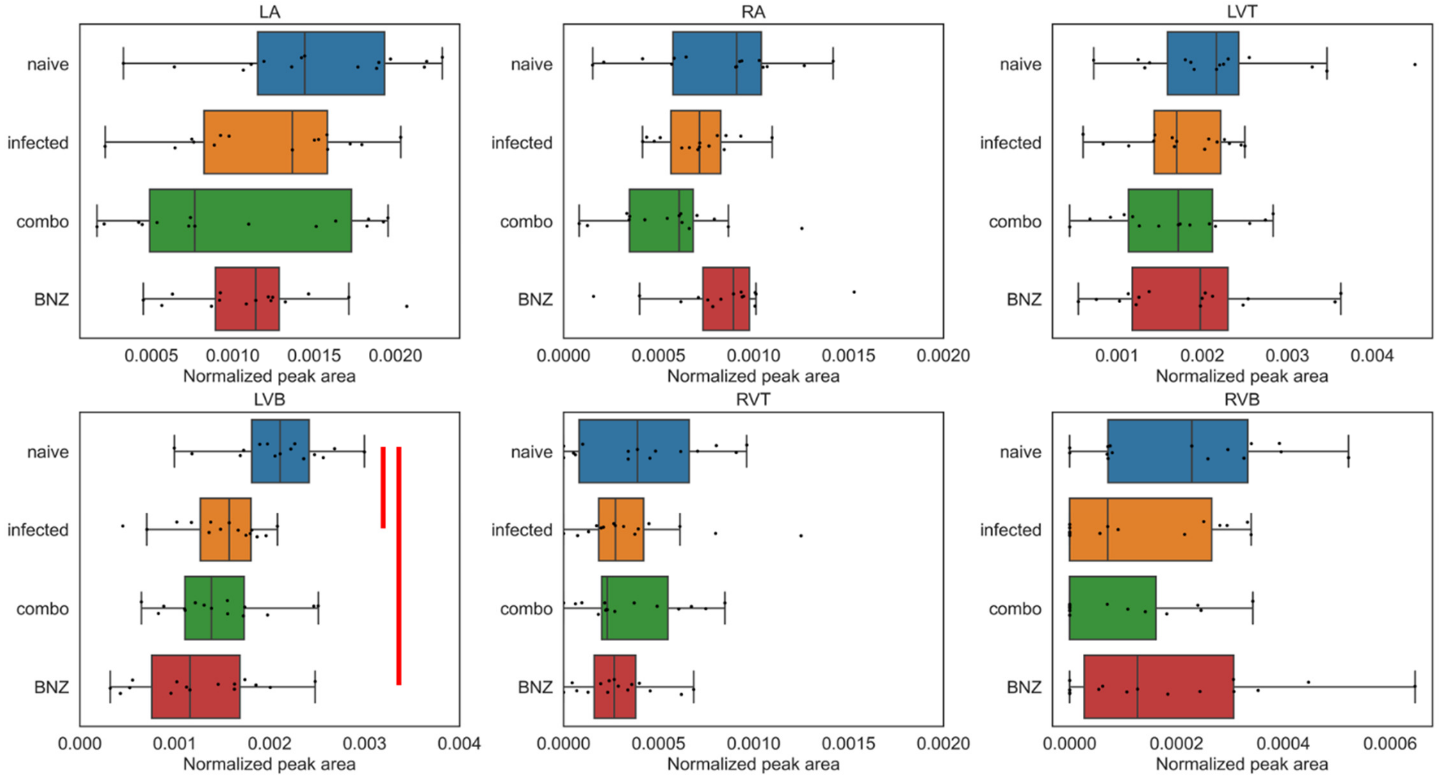

Supplementary Figure 25. Impact of treatment on glycerophosphocholines in *m/z* range 600 to 700. Data represents summed peak areas for glycerophosphocholines in *m/z* range 600 to 700, identified as described in Methods. Red line, p-value < 0.05 by Mann-Whitney U test, two-sided, FDR-corrected. Boxplots represent median, upper and lower quartiles, with whiskers extending to show the rest of the distribution, except for points that are determined to be outliers by being beyond the interquartile range +/- 1.5 times the interquartile range. LA, left atrium. RA, right atrium. LVT, left ventricle top. LVB, left ventricle bottom. RVT, right ventricle top. RVB, right ventricle bottom. BNZ, benznidazole. N=15 mice per group and per position. Source data are provided as a Source Data file.

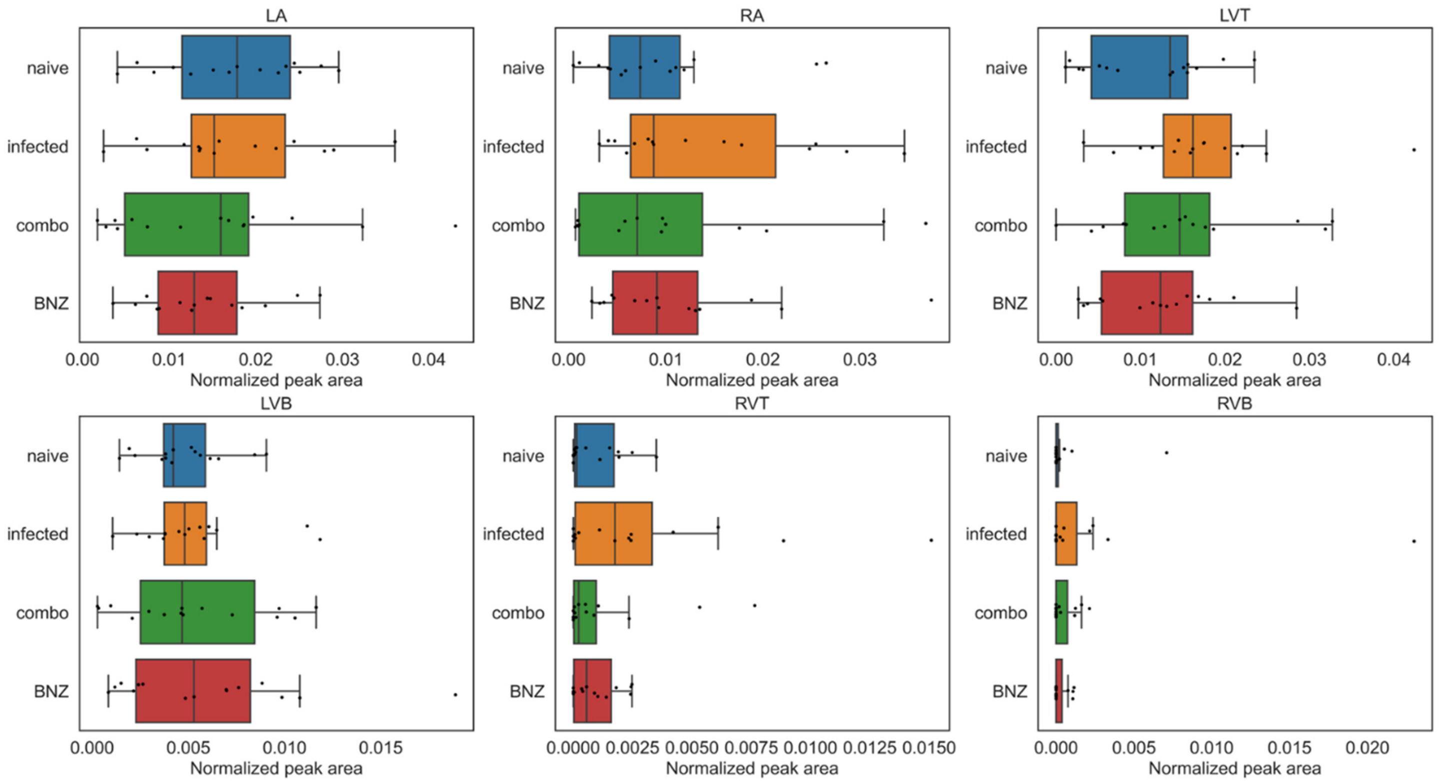

Supplementary Figure 26. Impact of treatment on glycerophosphocholines in *m/z* range 700 to 800. Data represents summed peak areas for glycerophosphocholines in *m/z* range 700 to 800, identified as described in Methods. Red line, p-value < 0.05 by Mann-Whitney U test, two-sided, FDR-corrected. Boxplots represent median, upper and lower quartiles, with whiskers extending to show the rest of the distribution, except for points that are determined to be outliers by being beyond the interquartile range +/- 1.5 times the interquartile range. LA, left atrium. RA, right atrium. LVT, left ventricle top. LVB, left ventricle bottom. RVT, right ventricle top. RVB, right ventricle bottom. BNZ, benznidazole. N=15 mice per group and per position. Source data are provided as a Source Data file.

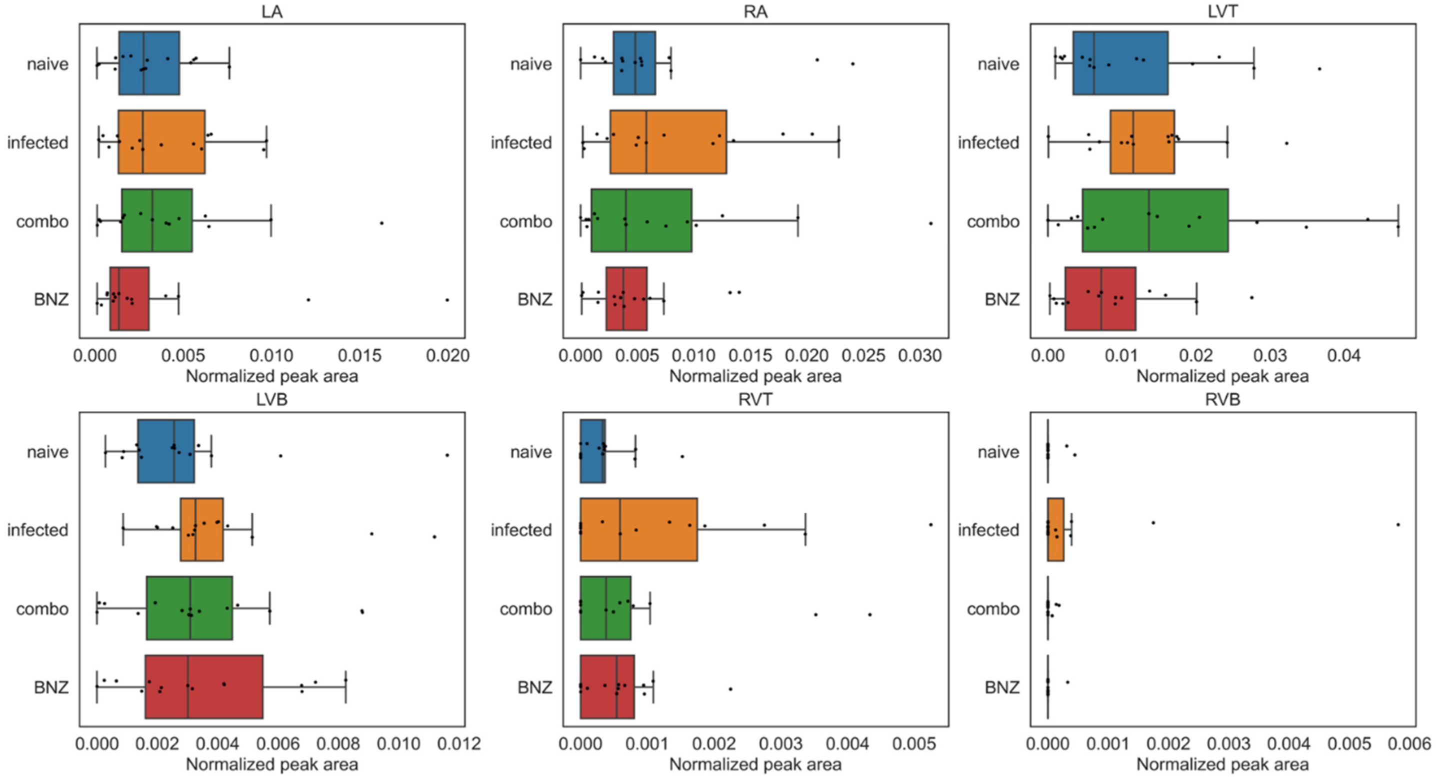

Supplementary Figure 27. Impact of treatment on glycerophosphocholines in *m/z* range 800 to 900. Data represents summed peak areas for glycerophosphocholines in *m/z* range 800 to 900, identified as described in Methods. Red line, p-value < 0.05 by Mann-Whitney U test, two-sided, FDR-corrected. Boxplots represent median, upper and lower quartiles, with whiskers extending to show the rest of the distribution, except for points that are determined to be outliers by being beyond the interquartile range +/- 1.5 times the interquartile range. LA, left atrium. RA, right atrium. LVT, left ventricle top. LVB, left ventricle bottom. RVT, right ventricle top. RVB, right ventricle bottom. BNZ, benznidazole. N=15 mice per group and per position. Source data are provided as a Source Data file.

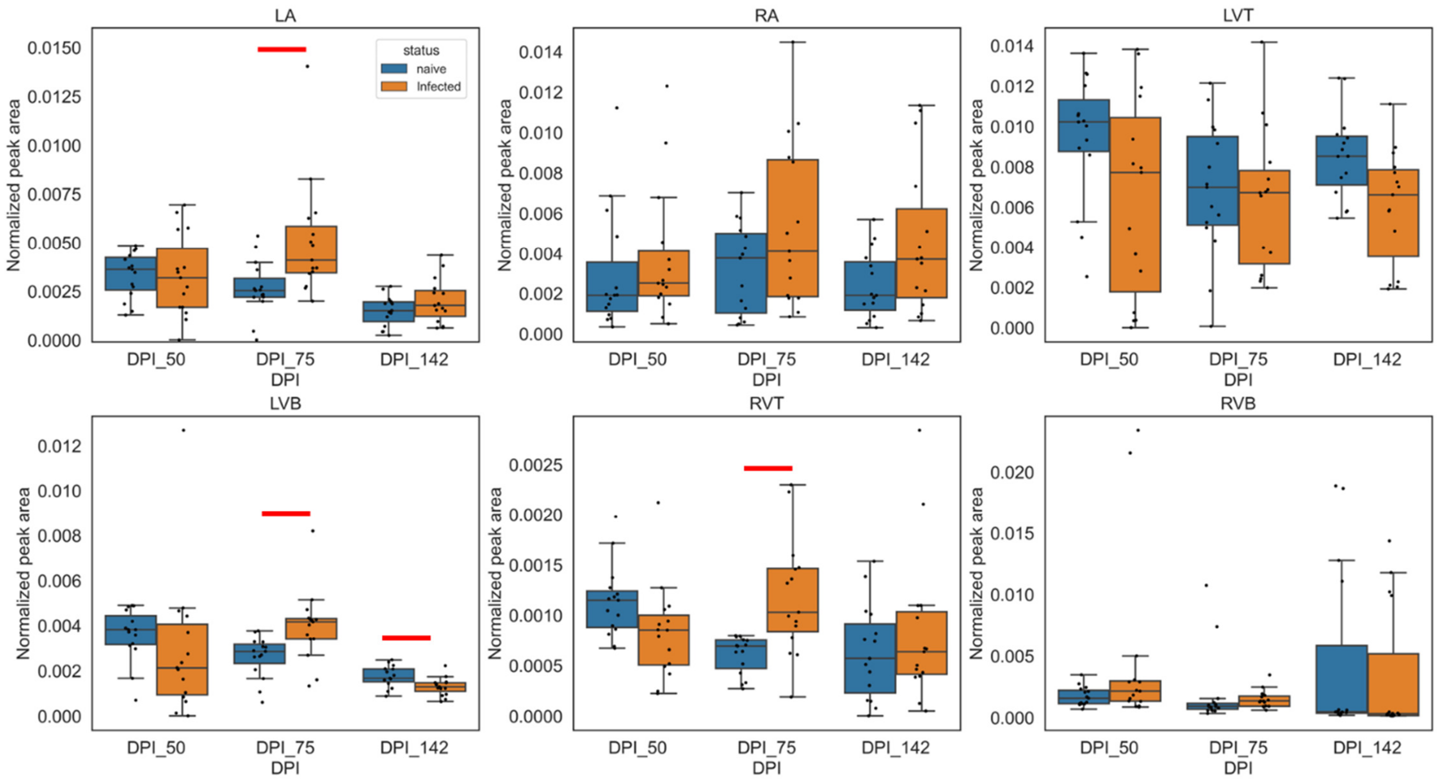

Supplementary Figure 28. Impact of infection duration on glycerophosphoethanolamines in *m/z* range 400 to 500. Data represents summed peak areas for all glycerophosphoethanolamines in *m/z* range 400 to 500, identified as described in Methods. DPI, days post-infection. LA, left atrium. RA, right atrium. LVT, left ventricle top. LVB, left ventricle bottom. RVT, right ventricle top. RVB, right ventricle bottom. Red line, p-value < 0.05 by Mann-Whitney U test, two-sided, FDR-corrected. Boxplots represent median, upper and lower quartiles, with whiskers extending to show the rest of the distribution, except for points that are determined to be outliers by being beyond the interquartile range +/- 1.5 times the interquartile range. N=15 mice per group and per position. Source data are provided as a Source Data file.

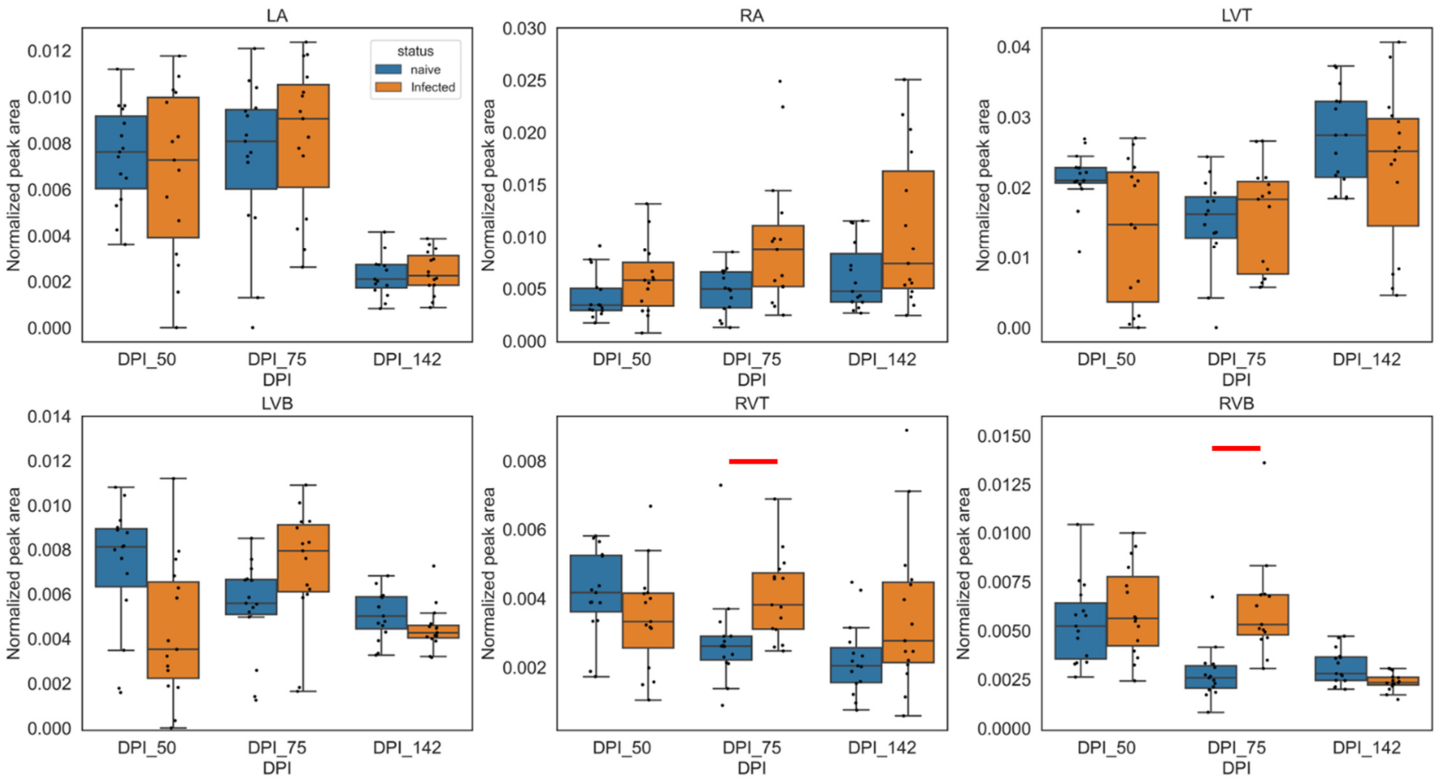

Supplementary Figure 29. Impact of infection duration on glycerophosphoethanolamines in *m/z* range 500 to 600. Data represents summed peak areas for all glycerophosphoethanolamines in *m/z* range 500 to 600, identified as described in Methods. DPI, days post-infection. LA, left atrium. RA, right atrium. LVT, left ventricle top. LVB, left ventricle bottom. RVT, right ventricle top. RVB, right ventricle bottom. Red line, p-value < 0.05 by Mann-Whitney U test, two-sided, FDR-corrected. Boxplots represent median, upper and lower quartiles, with whiskers extending to show the rest of the distribution, except for points that are determined to be outliers by being beyond the interquartile range +/- 1.5 times the interquartile range. N=15 mice per group and per position. Source data are provided as a Source Data file.

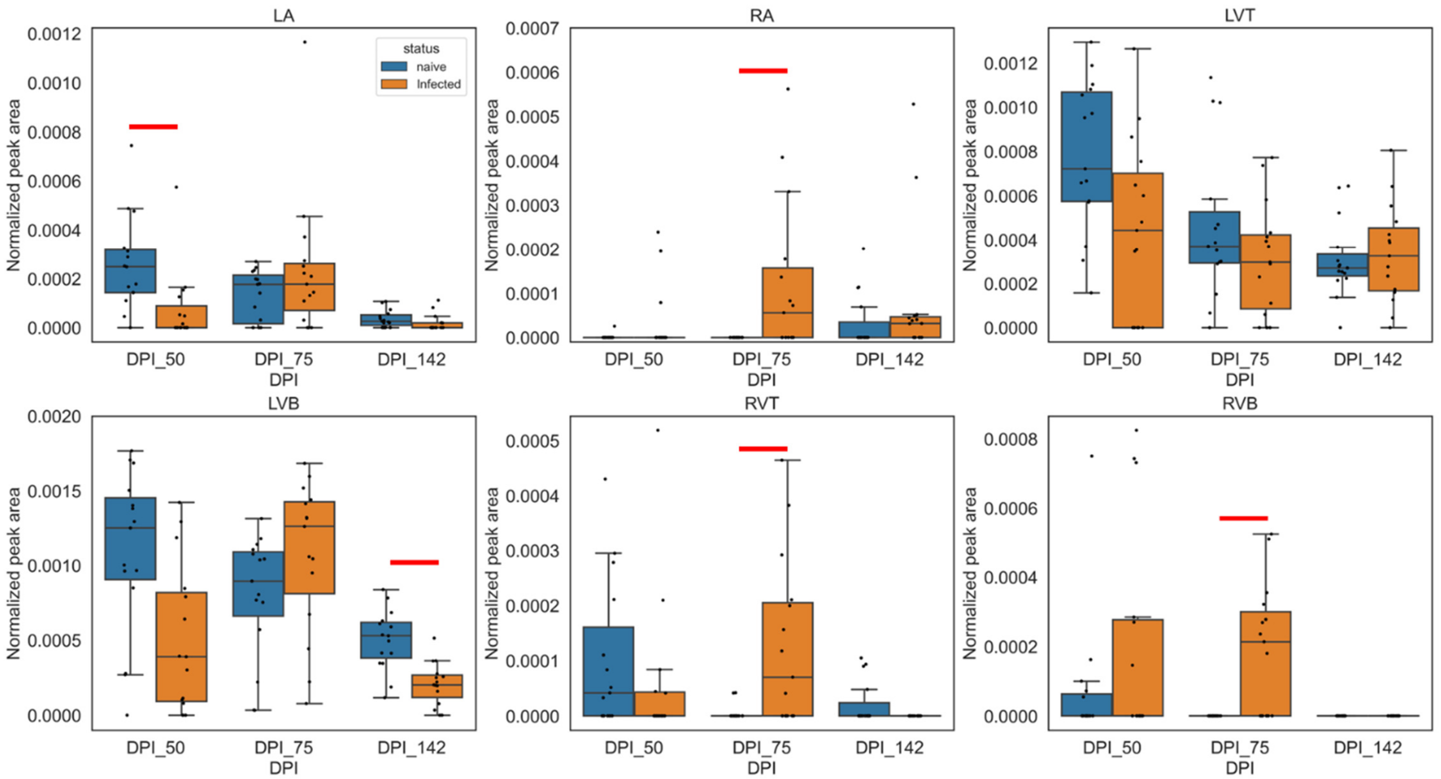

Supplementary Figure 30. Impact of infection duration on glycerophosphoethanolamines in *m/z* range 600 to 700. Data represents summed peak areas for all glycerophosphoethanolamines in *m/z* range 600 to 700, identified as described in Methods. DPI, days post-infection. LA, left atrium. RA, right atrium. LVT, left ventricle top. LVB, left ventricle bottom. RVT, right ventricle top. RVB, right ventricle bottom. Red line, p-value < 0.05 by Mann-Whitney U test, two-sided, FDR-corrected. Boxplots represent median, upper and lower quartiles, with whiskers extending to show the rest of the distribution, except for points that are determined to be outliers by being beyond the interquartile range +/- 1.5 times the interquartile range. N=15 mice per group and per position. Source data are provided as a Source Data file.

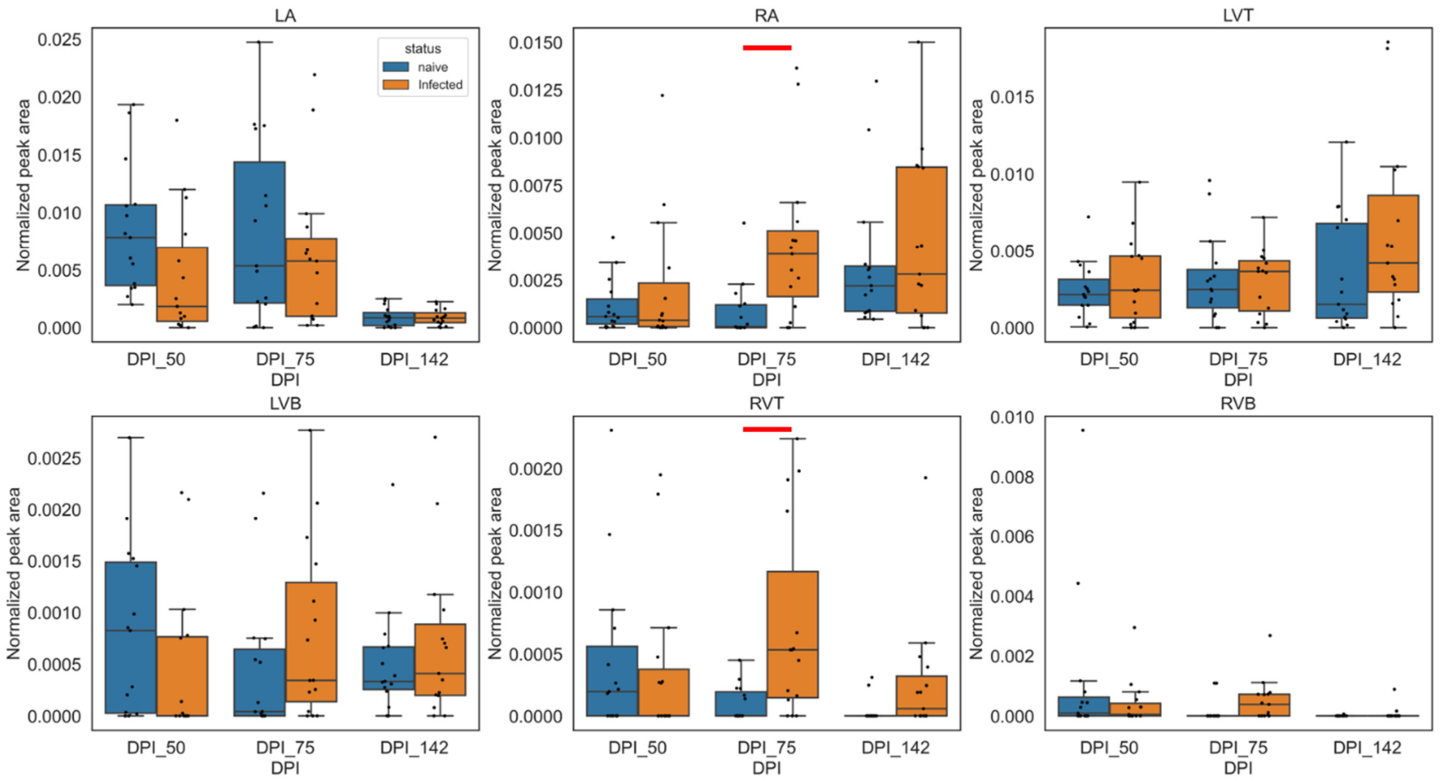

Supplementary Figure 31. Impact of infection duration on glycerophosphoethanolamines in *m/z* range 700 to 800. Data represents summed peak areas for all glycerophosphoethanolamines in *m/z* range 700 to 800, identified as described in Methods. DPI, days post-infection. LA, left atrium. RA, right atrium. LVT, left ventricle top. LVB, left ventricle bottom. RVT, right ventricle top. RVB, right ventricle bottom. Red line, p-value < 0.05 by Mann-Whitney U test, two-sided, FDR-corrected. Boxplots represent median, upper and lower quartiles, with whiskers extending to show the rest of the distribution, except for points that are determined to be outliers by being beyond the interquartile range +/- 1.5 times the interquartile range. N=15 mice per group and per position. Source data are provided as a Source Data file.

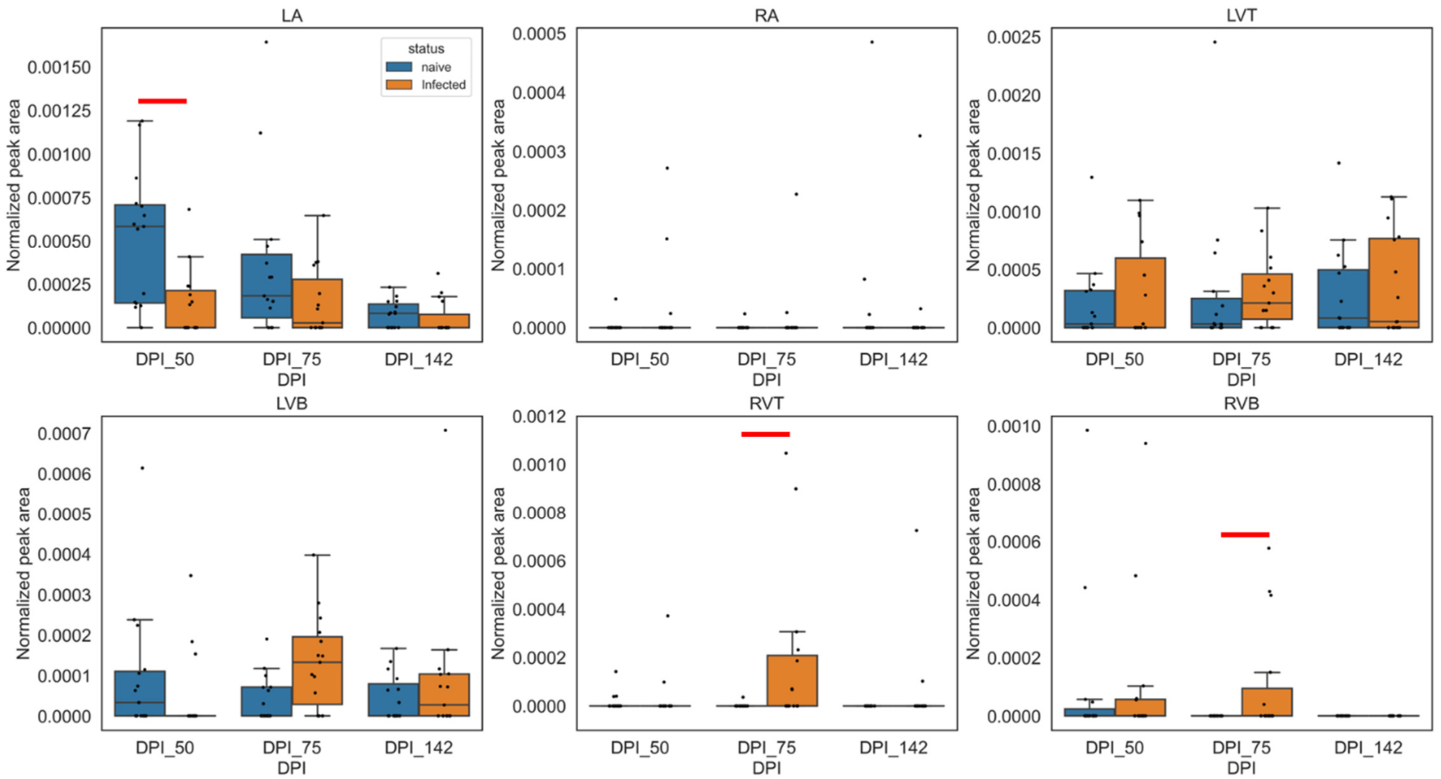

Supplementary Figure 32. Impact of infection duration on glycerophosphoethanolamines in *m/z* range 800 to 900. Data represents summed peak areas for all glycerophosphoethanolamines in *m/z* range 800 to 900, identified as described in Methods. DPI, days post-infection. LA, left atrium. RA, right atrium. LVT, left ventricle top. LVB, left ventricle bottom. RVT, right ventricle top. RVB, right ventricle bottom. Red line, p-value < 0.05 by Mann-Whitney U test, two-sided, FDR-corrected. Boxplots represent median, upper and lower quartiles, with whiskers extending to show the rest of the distribution, except for points that are determined to be outliers by being beyond the interquartile range +/- 1.5 times the interquartile range. N=15 mice per group and per position. Source data are provided as a Source Data file.

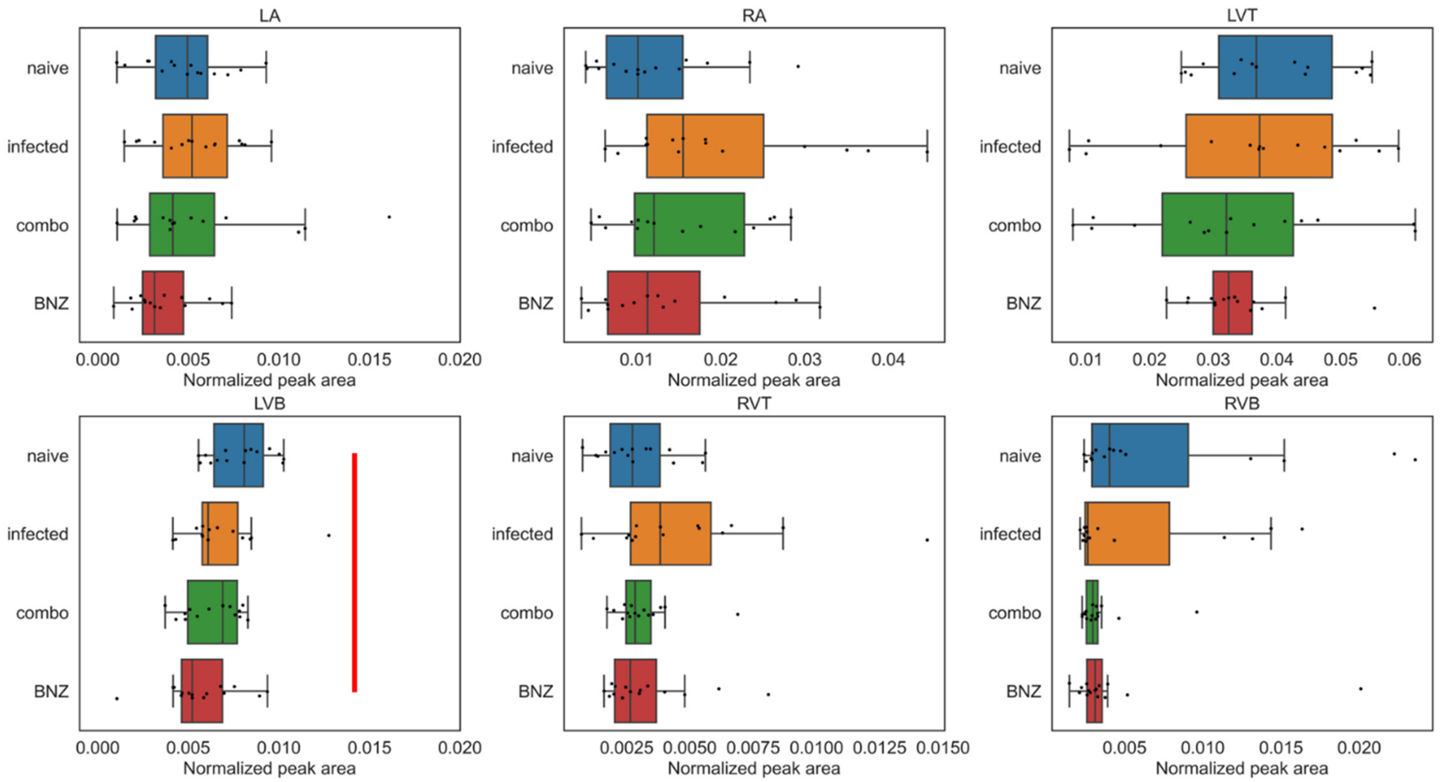

Supplementary Figure 33. Impact of treatment on total glycerophosphoethanolamines. Data represents summed peak areas for all glycerophosphoethanolamines, identified as described in Methods. Red line, p-value < 0.05 by Mann-Whitney U test, two-sided, FDR-corrected. Boxplots represent median, upper and lower quartiles, with whiskers extending to show the rest of the distribution, except for points that are determined to be outliers by being beyond the interquartile range +/- 1.5 times the interquartile range. LA, left atrium. RA, right atrium. LVT, left ventricle top. LVB, left ventricle bottom. RVT, right ventricle top. RVB, right ventricle bottom. BNZ, benznidazole. N=15 mice per group and per position. Source data are provided as a Source Data file.

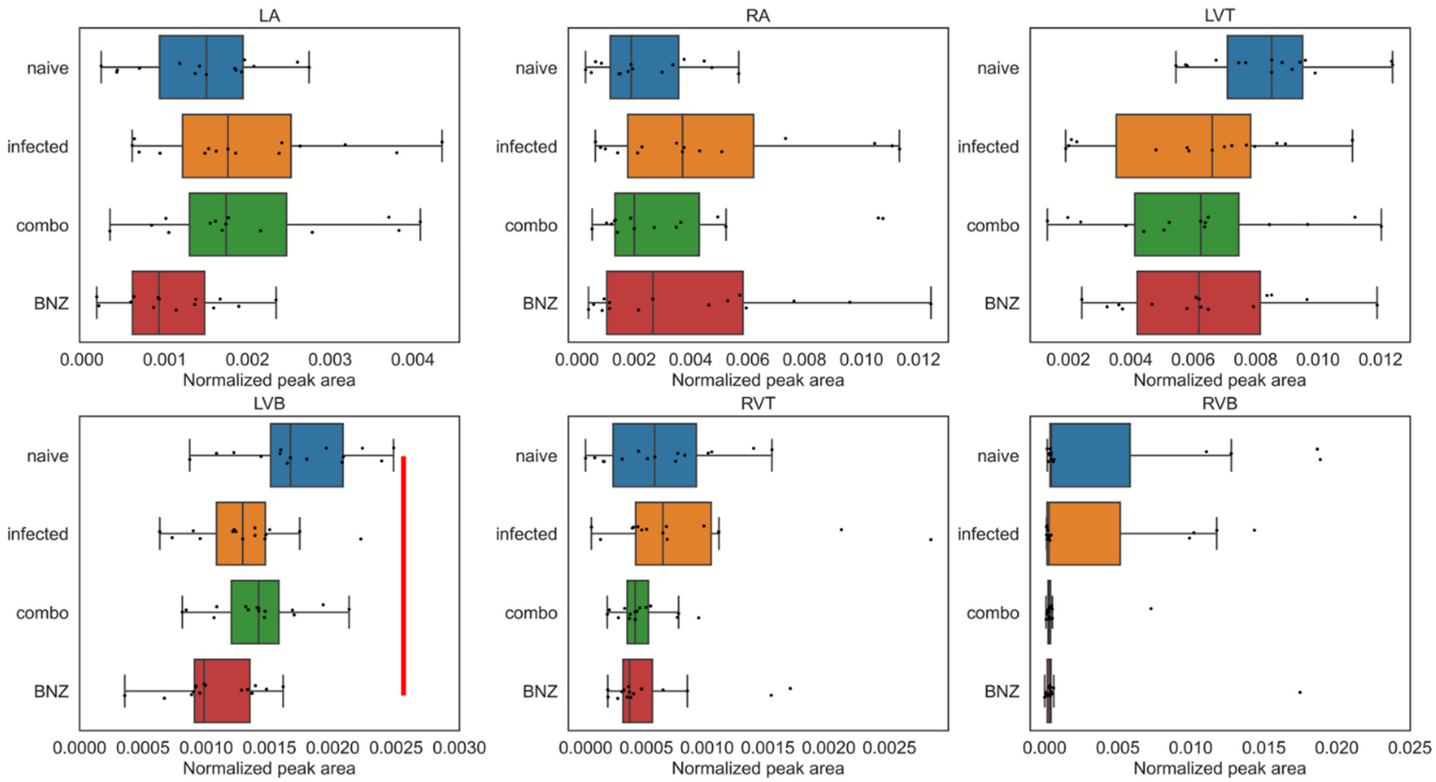

Supplementary Figure 34. Impact of treatment on glycerophosphoethanolamines in *m/z* range 400 to 500. Data represents summed peak areas for glycerophosphoethanolamines in *m/z* range 400 to 500, identified as described in Methods. Red line, p-value < 0.05 by Mann-Whitney U test, two-sided, FDR-corrected. Boxplots represent median, upper and lower quartiles, with whiskers extending to show the rest of the distribution, except for points that are determined to be outliers by being beyond the interquartile range +/- 1.5 times the interquartile range. LA, left atrium. RA, right atrium. LVT, left ventricle top. LVB, left ventricle bottom. RVT, right ventricle top. RVB, right ventricle bottom. BNZ, benznidazole. N=15 mice per group and per position. Source data are provided as a Source Data file.

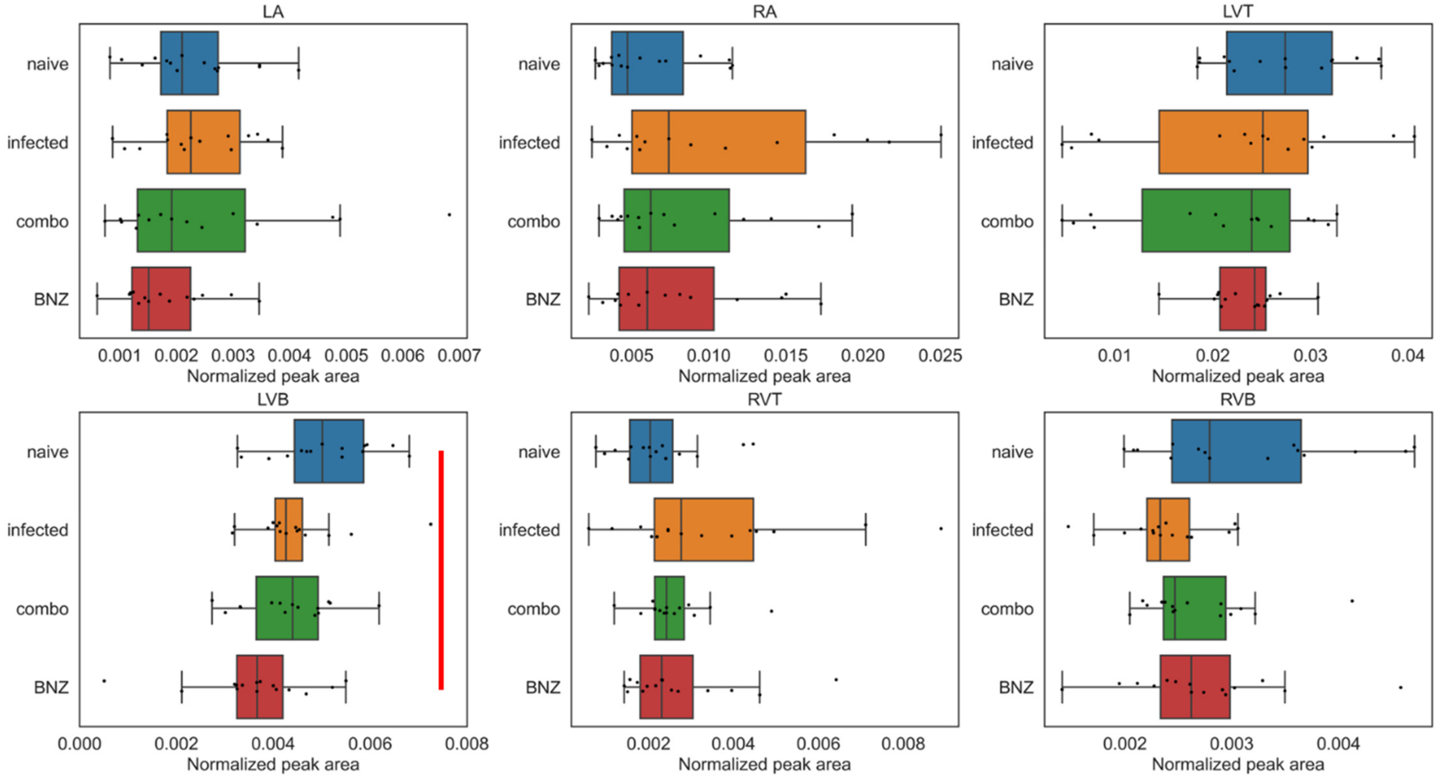

Supplementary Figure 35. Impact of treatment on glycerophosphoethanolamines in *m/z* range 500 to 600. Data represents summed peak areas for glycerophosphoethanolamines in *m/z* range 500 to 600, identified as described in Methods. Red line, p-value < 0.05 by Mann-Whitney U test, two-sided, FDR-corrected. Boxplots represent median, upper and lower quartiles, with whiskers extending to show the rest of the distribution, except for points that are determined to be outliers by being beyond the interquartile range +/- 1.5 times the interquartile range. LA, left atrium. RA, right atrium. LVT, left ventricle top. LVB, left ventricle bottom. RVT, right ventricle top. RVB, right ventricle bottom. BNZ, benznidazole. N=15 mice per group and per position. Source data are provided as a Source Data file.

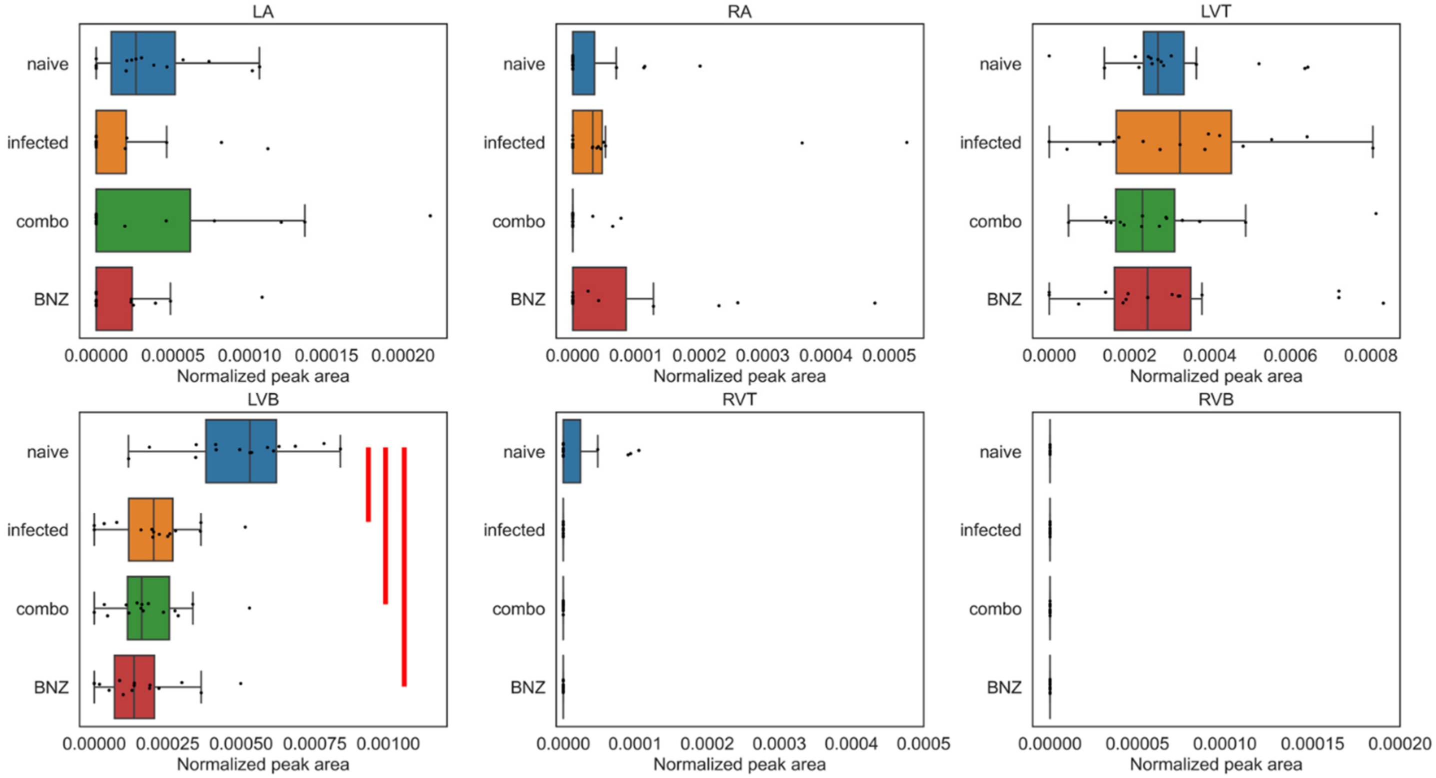

Supplementary Figure 36. Impact of treatment on glycerophosphoethanolamines in *m/z* range 600 to 700. Data represents summed peak areas for glycerophosphoethanolamines in *m/z* range 600 to 700, identified as described in Methods. Red line, p-value < 0.05 by Mann-Whitney U test, two-sided, FDR-corrected. Boxplots represent median, upper and lower quartiles, with whiskers extending to show the rest of the distribution, except for points that are determined to be outliers by being beyond the interquartile range +/- 1.5 times the interquartile range. LA, left atrium. RA, right atrium. LVT, left ventricle top. LVB, left ventricle bottom. RVT, right ventricle top. RVB, right ventricle bottom. BNZ, benznidazole. N=15 mice per group and per position. Source data are provided as a Source Data file.

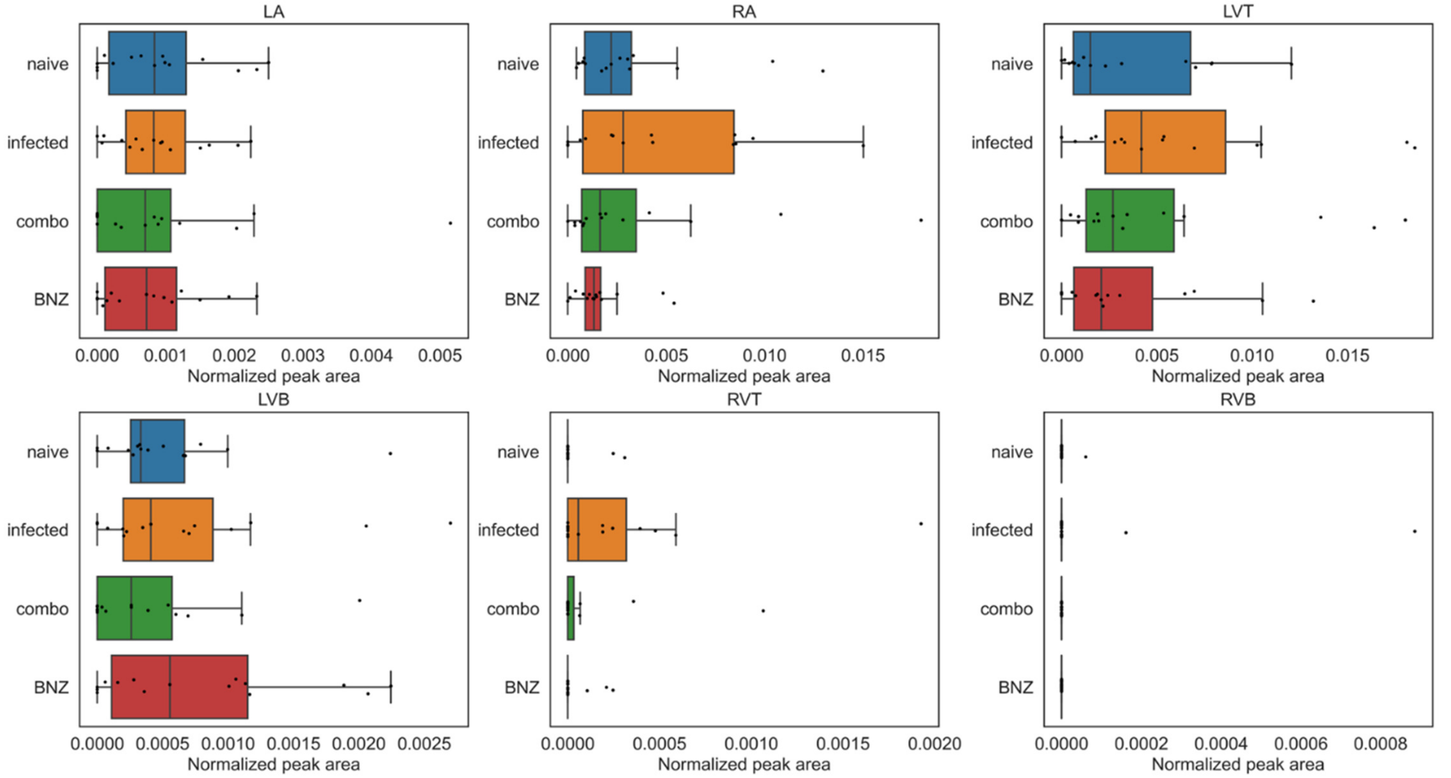

Supplementary Figure 37. Impact of treatment on glycerophosphoethanolamines in *m/z* range 700 to 800. Data represents summed peak areas for glycerophosphoethanolamines in *m/z* range 700 to 800, identified as described in Methods. Red line, p-value < 0.05 by Mann-Whitney U test, two-sided, FDR-corrected. Boxplots represent median, upper and lower quartiles, with whiskers extending to show the rest of the distribution, except for points that are determined to be outliers by being beyond the interquartile range +/- 1.5 times the interquartile range. LA, left atrium. RA, right atrium. LVT, left ventricle top. LVB, left ventricle bottom. RVT, right ventricle top. RVB, right ventricle bottom. BNZ, benznidazole. N=15 mice per group and per position. Source data are provided as a Source Data file.

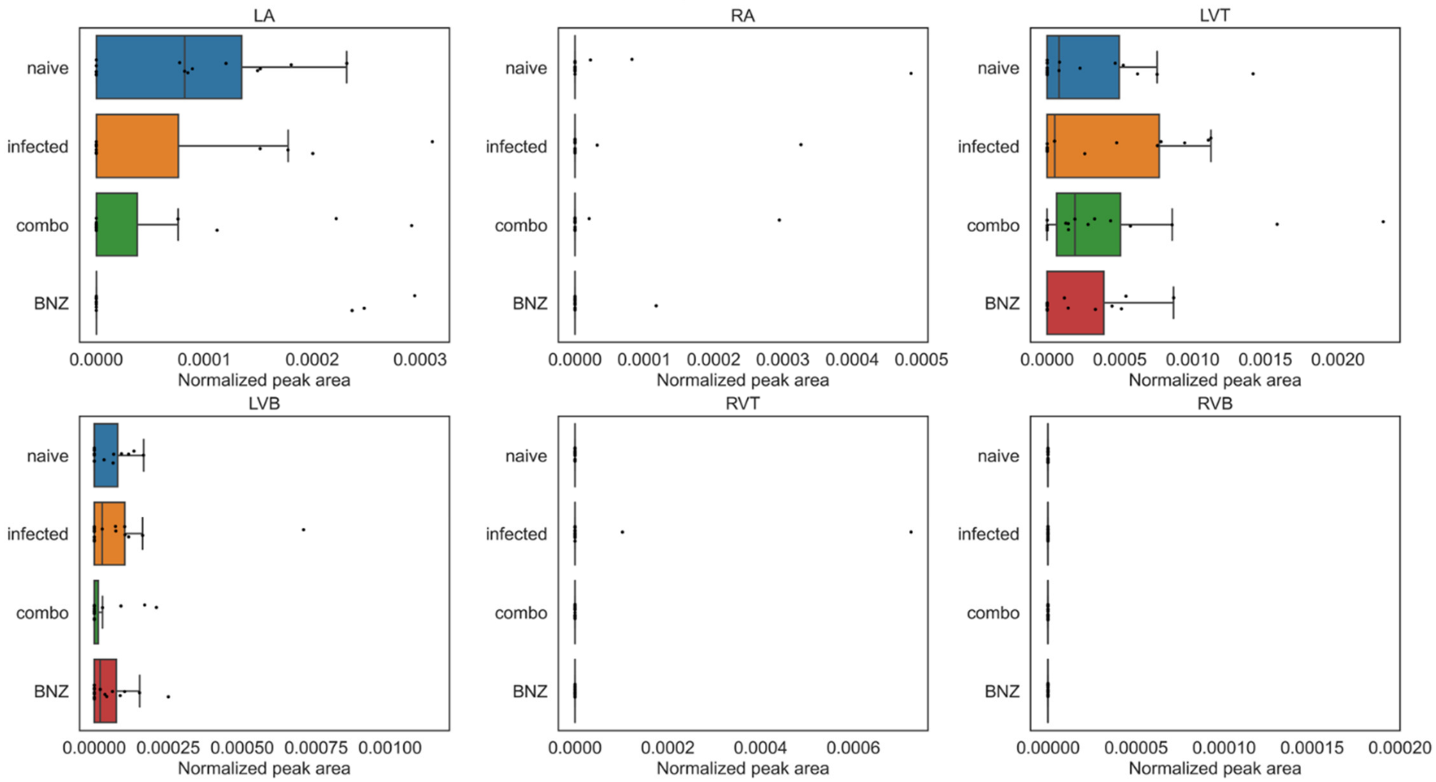

Supplementary Figure 38. Impact of treatment on glycerophosphoethanolamines in *m/z* range 800 to 900. Data represents summed peak areas for glycerophosphoethanolamines in *m/z* range 800 to 900, identified as described in Methods. Red line, p-value < 0.05 by Mann-Whitney U test, two-sided, FDR-corrected. Boxplots represent median, upper and lower quartiles, with whiskers extending to show the rest of the distribution, except for points that are determined to be outliers by being beyond the interquartile range +/- 1.5 times the interquartile range. LA, left atrium. RA, right atrium. LVT, left ventricle top. LVB, left ventricle bottom. RVT, right ventricle top. RVB, right ventricle bottom. BNZ, benznidazole. N=15 mice per group and per position. Source data are provided as a Source Data file.

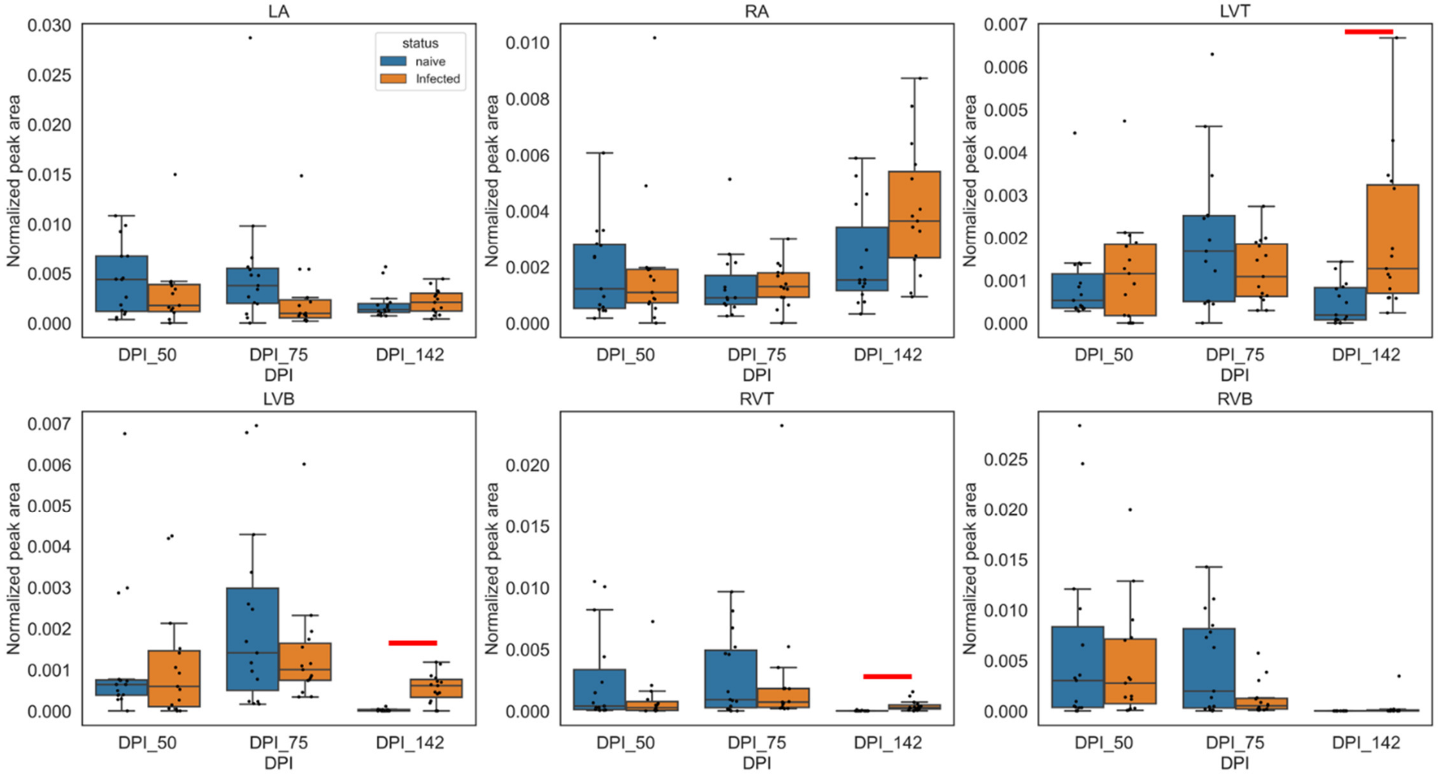

Supplementary Figure 39. Impact of infection duration on long chain acylcarnitines. Data represents summed peak areas for long chain acylcarnitines, identified as described in Methods. DPI, days post-infection. LA, left atrium. RA, right atrium. LVT, left ventricle top. LVB, left ventricle bottom. RVT, right ventricle top. RVB, right ventricle bottom. Red line, p-value < 0.05 by Mann-Whitney U test, two-sided, FDR-corrected. Boxplots represent median, upper and lower quartiles, with whiskers extending to show the rest of the distribution, except for points that are determined to be outliers by being beyond the interquartile range +/- 1.5 times the interquartile range. N=15 mice per group and per position. Source data are provided as a Source Data file.

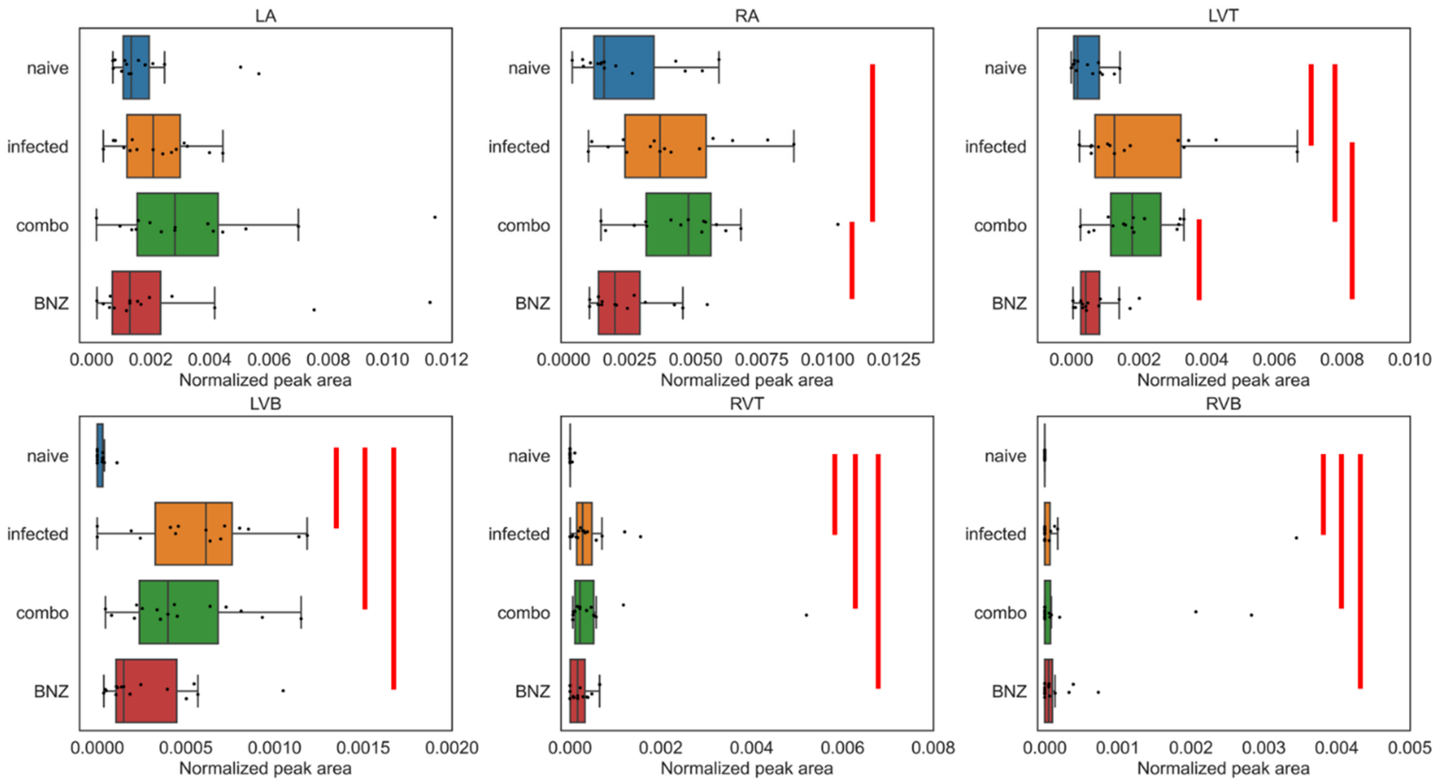

Supplementary Figure 40. Impact of treatment on long chain acylcarnitines. Data represents summed peak areas for long chain acylcarnitines, identified as described in Methods. Red line, p-value < 0.05 by Mann-Whitney U test, two-sided, FDR-corrected. Boxplots represent median, upper and lower quartiles, with whiskers extending to show the rest of the distribution, except for points that are determined to be outliers by being beyond the interquartile range +/- 1.5 times the interquartile range. LA, left atrium. RA, right atrium. LVT, left ventricle top. LVB, left ventricle bottom. RVT, right ventricle top. RVB, right ventricle bottom. BNZ, benznidazole. N=15 mice per group and per position. Source data are provided as a Source Data file.

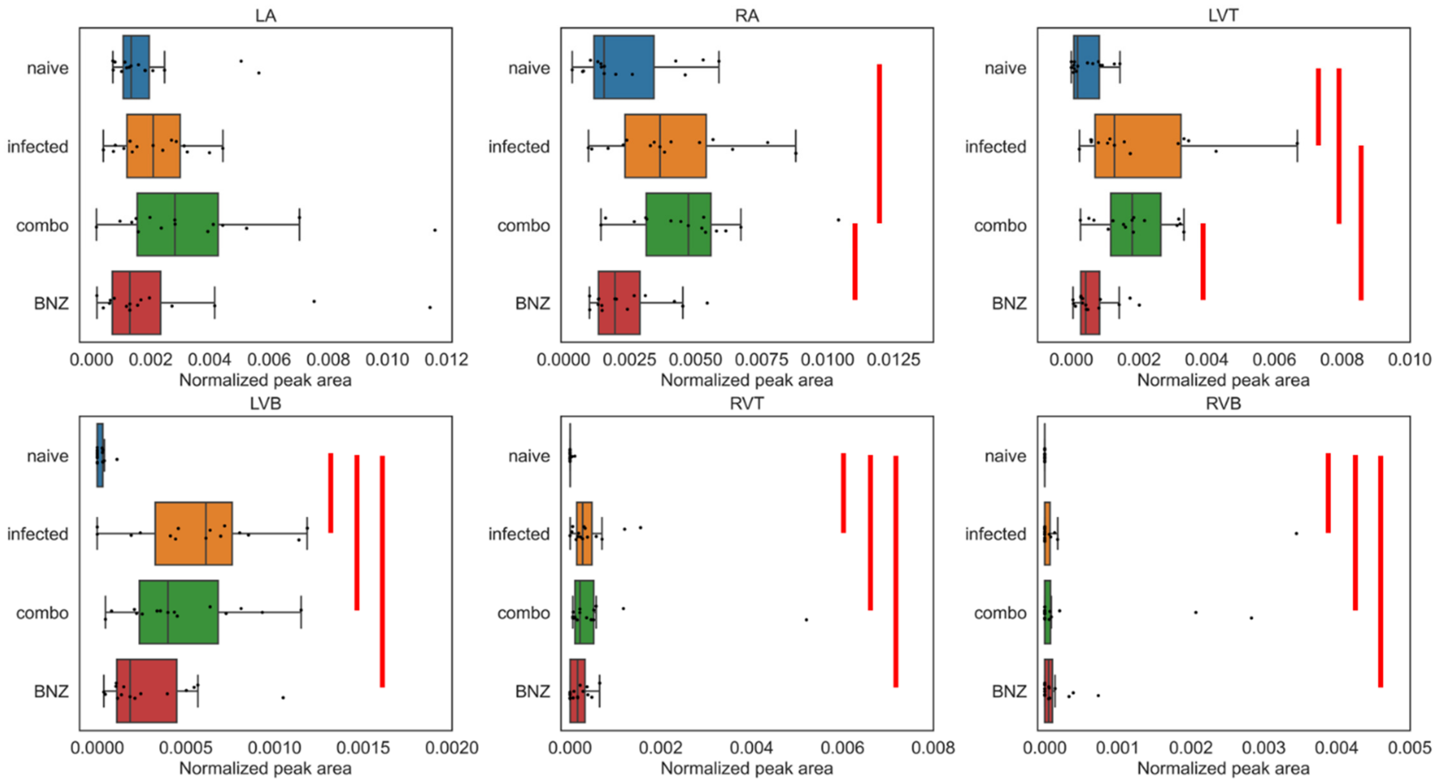

Supplementary Figure 41. Impact of treatment on total acylcarnitines. Data represents summed peak areas for total acylcarnitines, identified as described in Methods. Red line, p-value < 0.05 by Mann-Whitney U test, two-sided, FDR-corrected. Boxplots represent median, upper and lower quartiles, with whiskers extending to show the rest of the distribution, except for points that are determined to be outliers by being beyond the interquartile range +/- 1.5 times the interquartile range. LA, left atrium. RA, right atrium. LVT, left ventricle top. LVB, left ventricle bottom. RVT, right ventricle top. RVB, right ventricle bottom. BNZ, benznidazole. N=15 mice per group and per position. Source data are provided as a Source Data file.

Supplementary Figure 42. Heatmaps for treatment effects analysis, per heart position, clustering by features and by mouse. Source data are provided as a Source Data file. N=15 mice per group and per position. LA, left atrium. RA, right atrium. LVT, left ventricle top. RVT, right ventricle top. LVB, left ventricle bottom. RVB, right ventricle bottom.

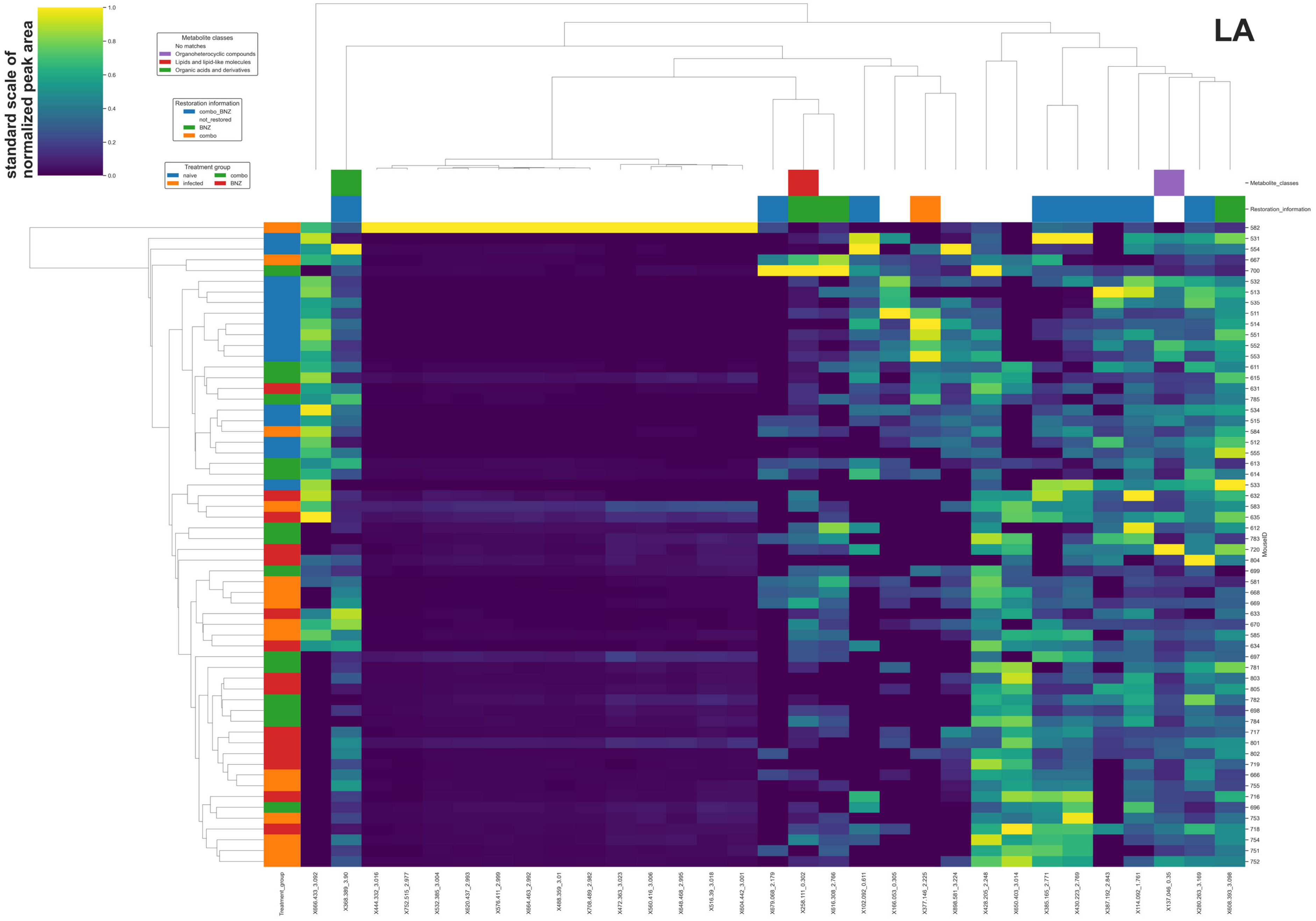

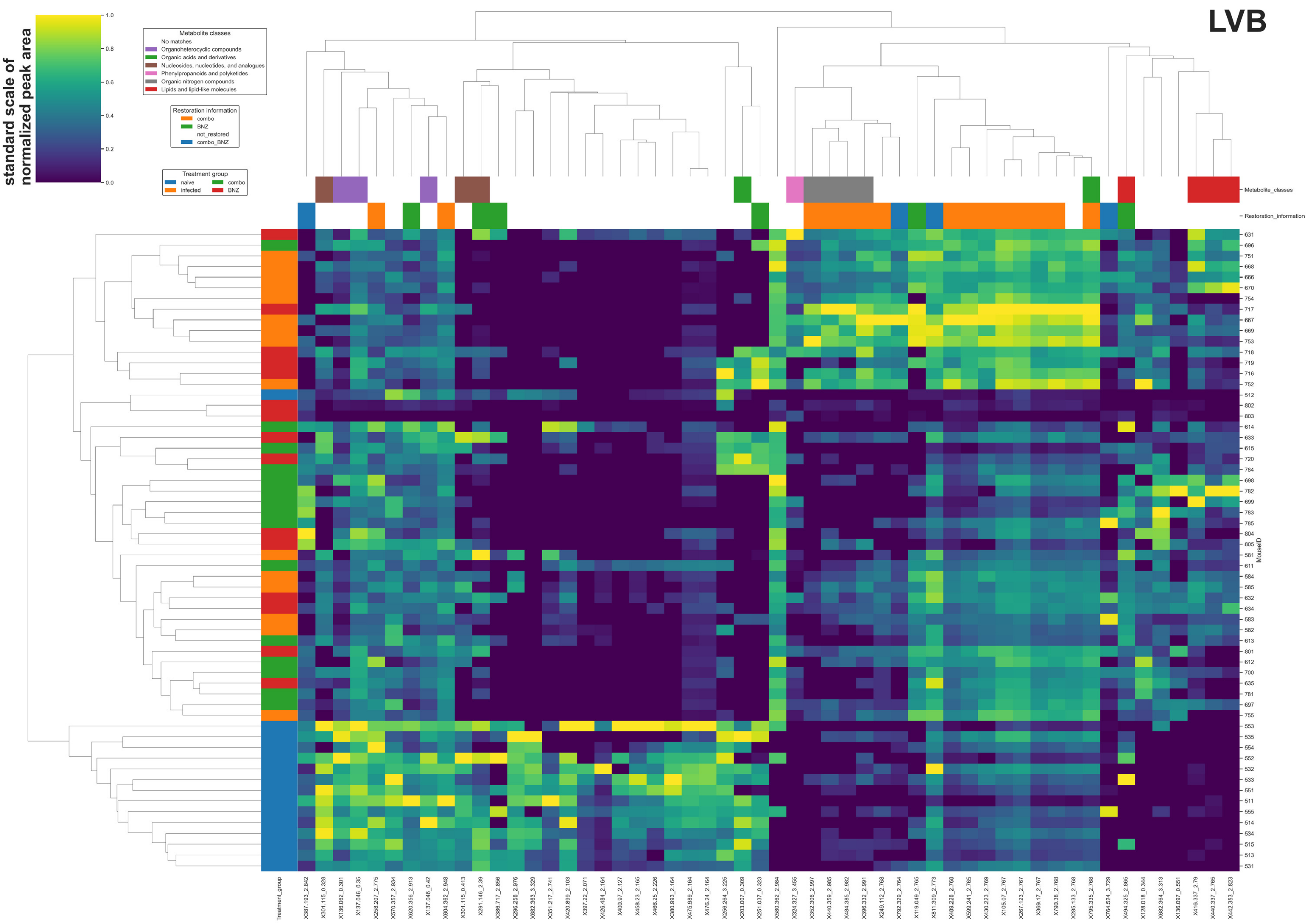
LVB
standard scale of normalized peak area
1.0
0.8
0.6
0.4
0.2
0.0
Metabolite classes
No matches
Organoheterocyclic compounds
Organic acids and derivatives
Nucleosides, nucleotides, and analogues
Phenylpropanoids and polyketides
Organic nitrogen compounds
Lipids and lipid-like molecules
Restoration information
combo
BNZ
not_restored
combo_BNZ
Treatment group
naive
combo
infected
BNZ
Metabolite_classes
Restoration_information
MouseID
Treatment_group
X387.193_2.842
X301.115_0.328
X136.062_0.301
X137.046_0.35
X258.207_2.775
X570.357_2.934
X620.356_2.913
X137.046_0.42
X604.362_2.948
X301.115_0.413
X291.146_2.39
X386.717_2.856
X296.258_2.976
X682.363_3.329
X351.217_2.741
X420.899_2.103
X397.22_2.071
X426.484_2.164
X400.97_2.127
X458.23_2.165
X466.25_2.226
X380.993_2.164
X475.989_2.164
X476.24_2.164
X256.264_3.225
X203.007_0.309
X251.037_0.323
X580.362_2.984
X324.327_3.455
X352.306_2.997
X440.359_2.985
X484.385_2.982
X396.332_2.991
X249.112_2.768
X792.329_2.764
X119.049_2.765
X811.309_2.773
X489.228_2.768
X599.241_2.765
X430.223_2.769
X105.07_2.767
X267.123_2.767
X369.17_2.767
X790.38_2.768
X285.133_2.768
X795.335_2.768
X764.524_3.729
X494.325_2.865
X128.018_0.344
X682.364_3.313
X136.097_0.551
X416.337_2.79
X440.337_2.765
X442.353_2.823
631
696
751
668
666
670
754
717
667
669
753
718
719
716
752
512
802
803
614
633
615
720
784
698
782
699
783
785
804
805
581
611
584
585
632
634
583
582
613
801
612
700
635
781
697
755
553
535
554
552
532
533
551
511
555
514
534
515
513
531

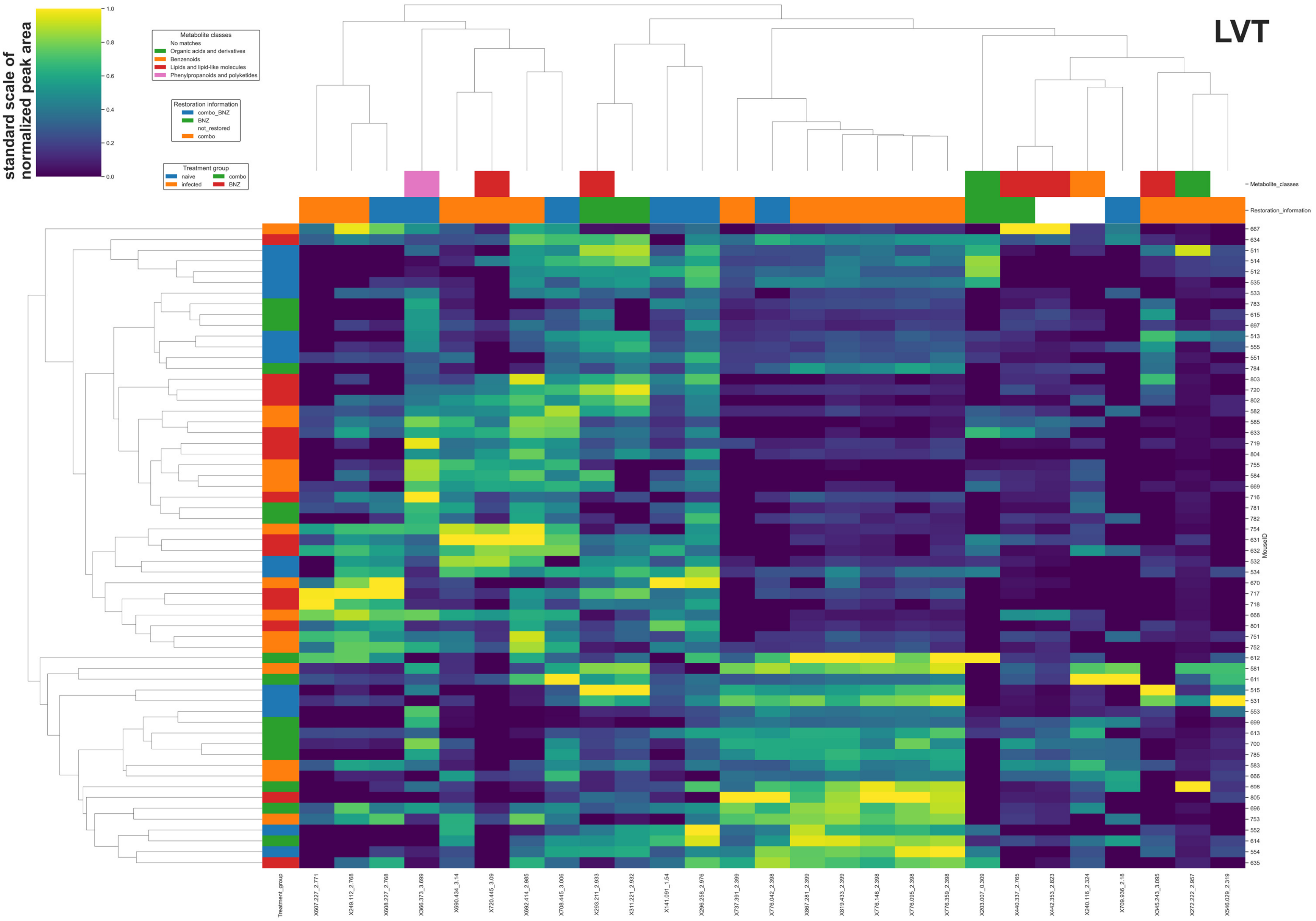
LVT
standard scale of normalized peak area
1.0
0.8
0.6
0.4
0.2
0.0
Metabolite classes
No matches
Organic acids and derivatives
Benzenoids
Lipids and lipid-like molecules
Phenylpropanoids and polyketides
Restoration information
combo_BNZ
BNZ
not_restored
combo
Treatment group
naive
combo
infected
BNZ
Metabolite_classes
Restoration_information
MouseID
667
634
511
514
512
535
533
783
615
697
513
555
551
784
803
720
802
582
585
633
719
804
755
584
669
716
781
782
754
631
632
532
534
670
717
718
668
801
751
752
612
581
611
515
531
553
699
613
700
785
583
666
698
805
696
753
552
614
554
635
Treatment_group
X607.227_2.771
X249.112_2.768
X608.227_2.768
X366.373_3.699
X690.434_3.14
X720.445_3.09
X692.414_2.985
X708.445_3.006
X293.211_2.933
X311.221_2.932
X141.091_1.54
X296.258_2.976
X737.391_2.399
X776.042_2.398
X867.281_2.399
X819.433_2.399
X776.148_2.398
X776.095_2.398
X776.359_2.398
X203.007_0.309
X440.337_2.765
X442.353_2.823
X240.116_2.324
X709.936_2.18
X345.243_3.095
X272.222_2.957
X546.029_2.319

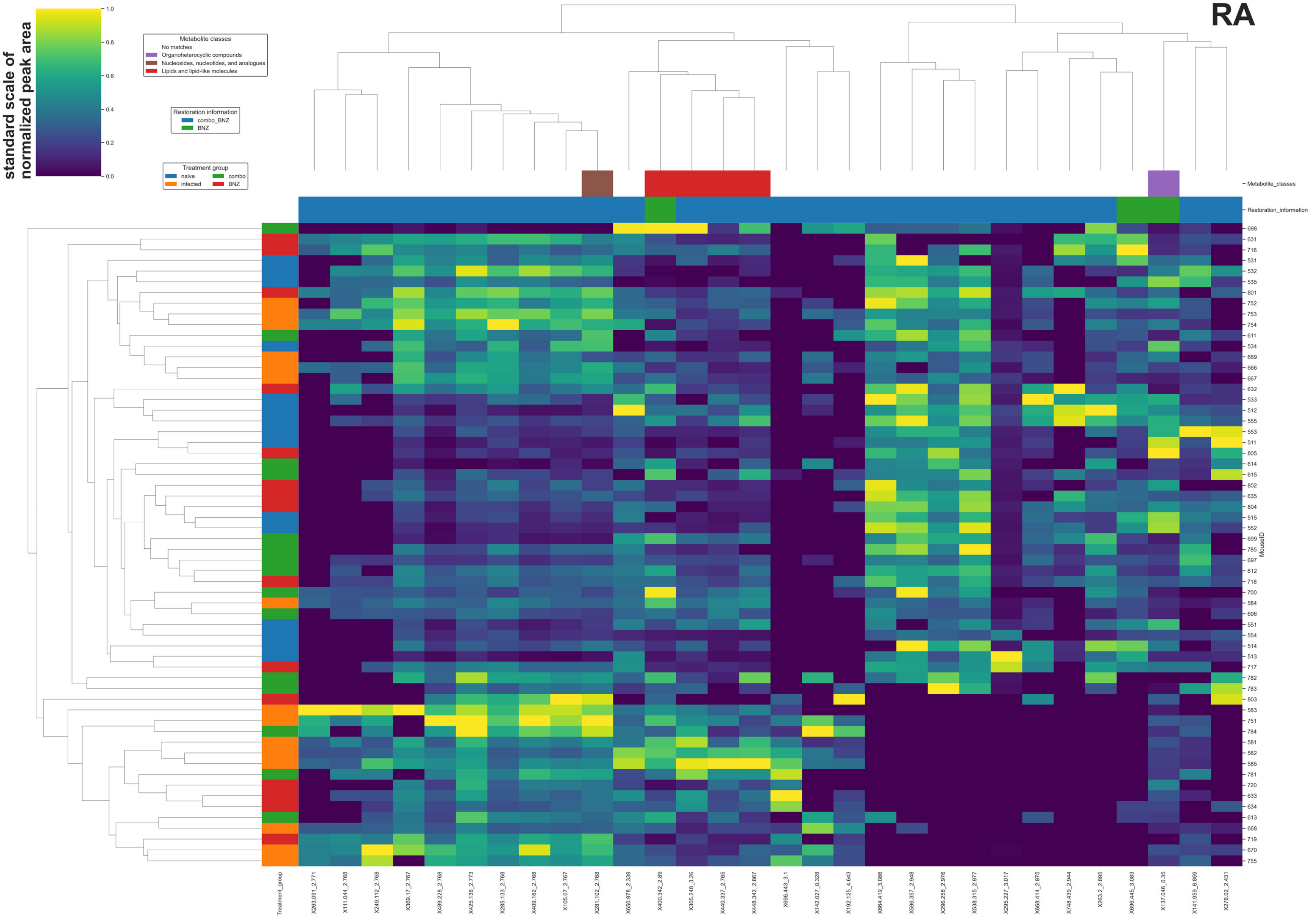
RA
standard scale of normalized peak area
0.0
0.2
0.4
0.6
0.8
1.0
Metabolite classes
No matches
Organoheterocyclic compounds
Nucleosides, nucleotides, and analogues
Lipids and lipid-like molecules
Restoration information
combo_BNZ
BNZ
Treatment group
naive
infected
combo
BNZ
Metabolite_classes
Restoration_information
MouseID
698
631
716
531
532
535
801
752
753
754
611
534
669
666
667
632
533
512
555
553
511
805
614
615
802
635
804
515
552
699
785
697
612
718
700
584
696
551
554
514
513
717
782
783
803
583
751
784
581
582
585
781
720
633
634
613
668
719
670
755
Treatment_group
X263.091_2.771
X111.044_2.768
X249.112_2.768
X369.17_2.767
X489.228_2.768
X425.136_2.773
X285.133_2.768
X409.162_2.768
X105.07_2.767
X281.102_2.768
X650.978_2.339
X400.342_2.89
X305.248_3.26
X440.337_2.765
X448.342_2.867
X696.443_3.1
X142.027_0.328
X192.125_4.643
X664.419_3.086
X596.357_2.948
X296.258_2.976
X538.315_2.977
X295.227_3.017
X668.414_2.975
X748.439_2.944
X263.2_2.895
X696.445_3.083
X137.046_0.35
X141.959_6.859
X276.02_2.431

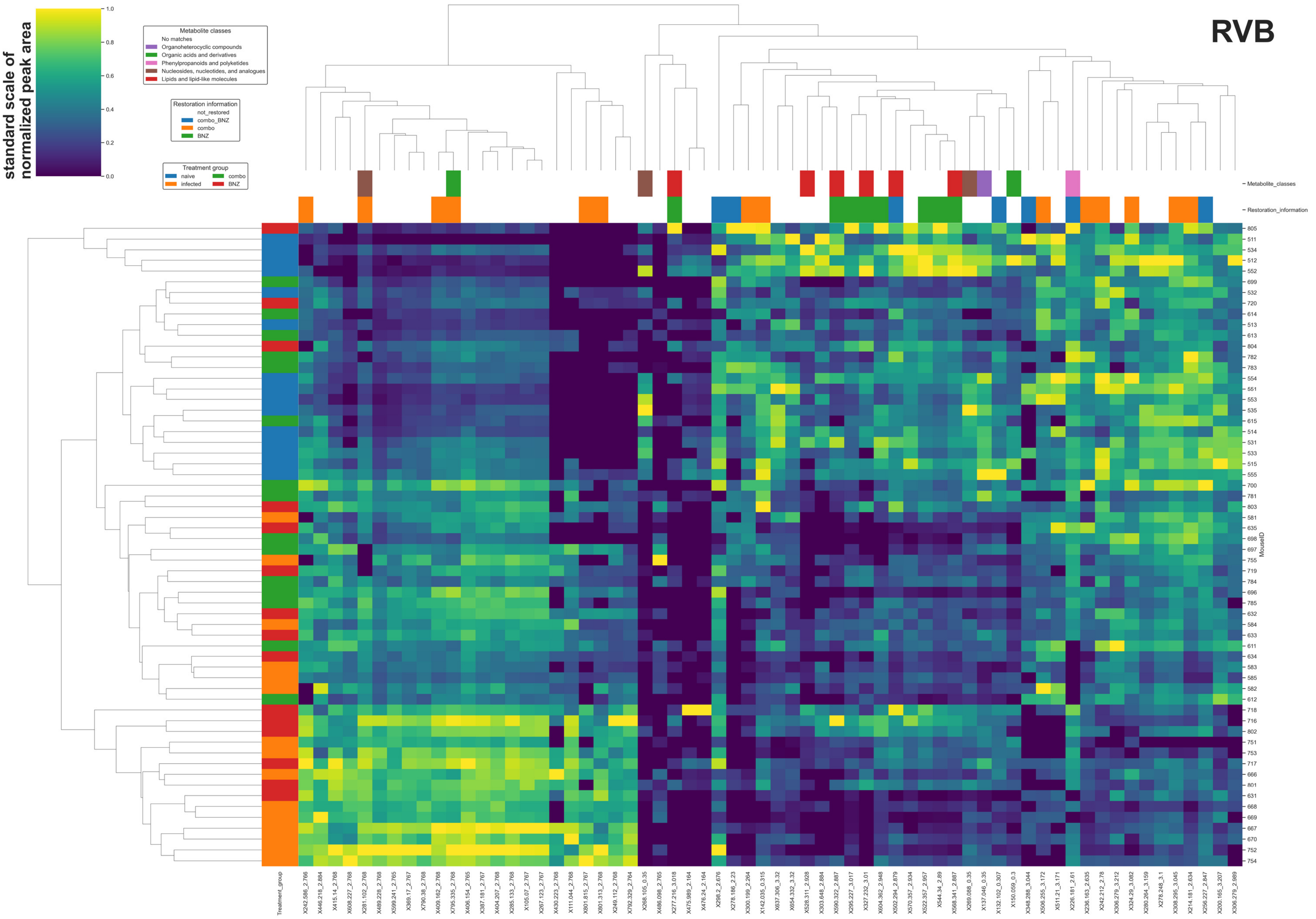
RVB
standard scale of normalized peak area
Metabolite classes
No matches
Organoheterocyclic compounds
Organic acids and derivatives
Phenylpropanoids and polyketides
Nucleosides, nucleotides, and analogues
Lipids and lipid-like molecules
Restoration information
not_restored
combo_BNZ
combo
BNZ
Treatment group
naive
combo
infected
BNZ
Metabolite_classes
Restoration_information
MouseID
Treatment_group

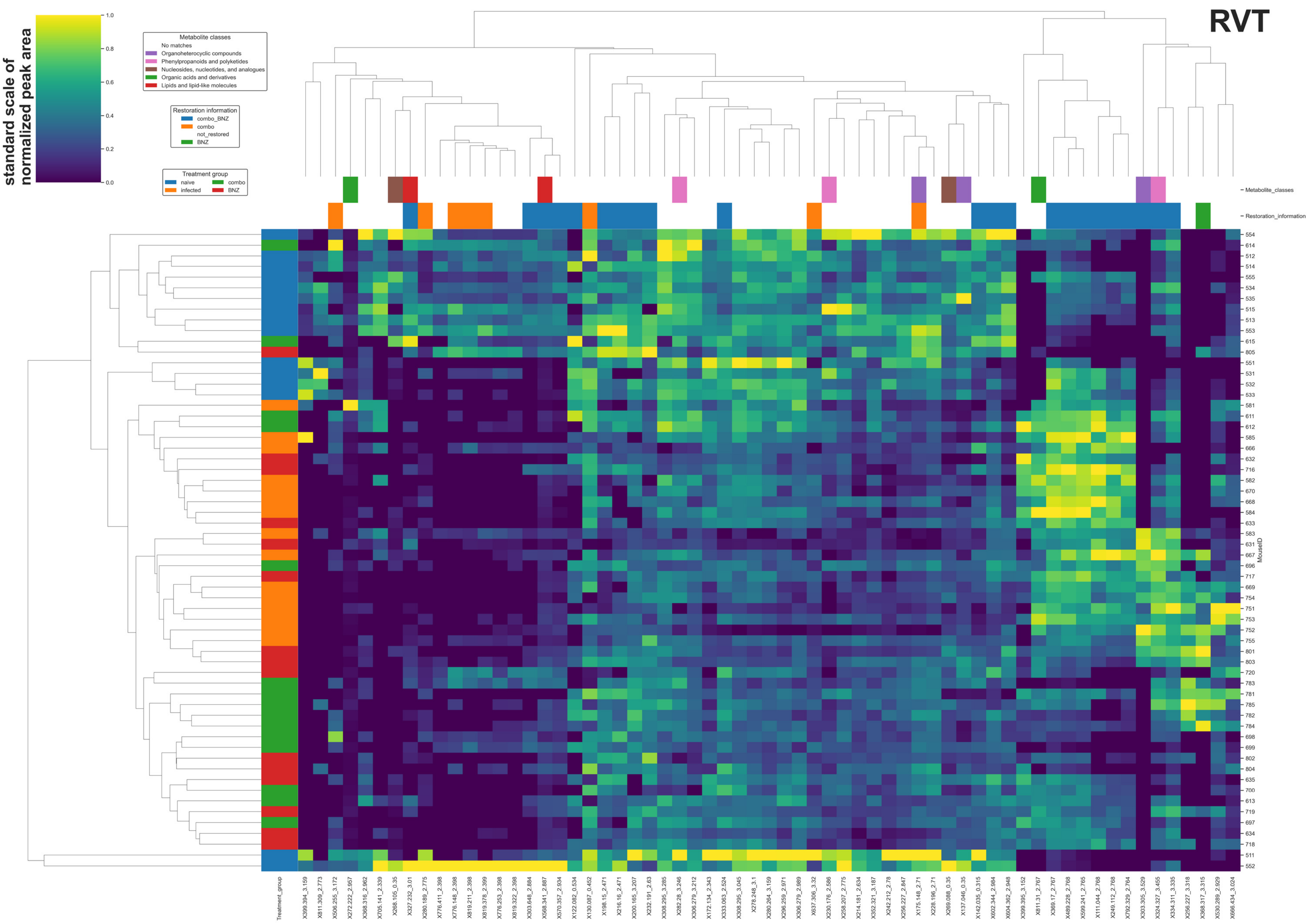
RVT
standard scale of normalized peak area
Metabolite classes
No matches
Organoheterocyclic compounds
Phenylpropanoids and polyketides
Nucleosides, nucleotides, and analogues
Organic acids and derivatives
Lipids and lipid-like molecules
Restoration information
combo_BNZ
combo
not_restored
BNZ
Treatment group
naive
infected
combo
BNZ
Metabolite_classes
Restoration_information
MouseID
Treatment_group

Supplementary Table 13. Proportion of features in each outcome group and position that were correlated to metadata, at 142 DPI. We sought to determine whether features restored by the different treatments at 142 days post-infection (DPI) or that failed to be restored presented differential association with the disease severity metadata. Specifically, we observed that a greater proportion of not-restored small molecule features were correlated with metadata at 142 DPI in the ventricles, compared to restored small molecule features. There was no clear differentiation between treatment groups. ("/" means that there were no small molecule features correlated with disease severity metadata in that section). N=15 mice per group and per position. Source data are provided as a Source Data file. LA, left atrium. RA, right atrium. LVT, left ventricle top. LVB, left ventricle bottom. RVT, right ventricle top. RVB, right ventricle bottom. BNZ, benznidazole.

Section	Restored by any treatment	Restored by BNZ+Tc24 combination	Restored by BNZ-only treatment	Restored by both treatments	Not restored by any treatment
LA	58%	100%	67%	50%	74%
RA	23%	/	67%	22%	/
LVT	16%	7%	75%	29%	100%
LVB	4%	7%	50%	75%	72%
RVT	35%	25%	/	50%	63%
RVB	10%	36%	/	/	91%

Supplementary Table 14. Proportion of features in each outcome group and position that were correlated to metadata, at 75 DPI. We then sought to determine whether these same features (restored or not by treatment at 142 DPI) had pre-existing correlations between their peak area at 75 DPI and the disease severity indicators at 75 DPI. Only a few of these restored or not-restored features were correlated with metadata at 75 DPI. Nevertheless, we observed that a greater proportion of not-restored small molecule features were correlated with metadata at 75 days post-infection in the left ventricle bottom, compared to restored small molecule features. The opposite pattern was observed in the right ventricle bottom. There was no clear differentiation between treatment groups**.** ("/" means that there is no small molecule correlated with disease severity metadata). N=15 mice per group and per position. Source data are provided as a Source Data file. LA, left atrium. RA, right atrium. LVT, left ventricle top. LVB, left ventricle bottom. RVT, right ventricle top. RVB, right ventricle bottom. BNZ, benznidazole.

Section	Restored by any treatment	Restored by BNZ+Tc24 combination	Restored by BNZ-only treatment	Restored by both treatments	Not restored by any treatment
LA	/	/	/	/	/
RA	/	/	/	/	/
LVT	/	/	/	/	/
LVB	16%	7%	83%	/	48%
RVT	/	/	/	/	/
RVB	10%	/	38%	14%	6%

Supplementary Table 15. Proportion of features in each outcome group and position that were correlated to multiple metadata parameters, at 142 DPI. There was no clear association between treatments, restored or not-restored small molecule features, and being correlated with more than one disease severity metadata parameter. N=15 mice per group and per position. LA, left atrium. RA, right atrium. LVT, left ventricle top. LVB, left ventricle bottom. RVT, right ventricle top. RVB, right ventricle bottom. Source data are provided as a Source Data file.

Sections	Restored by any treatment	Not restored	Fisher's exact test (two-tailed)
LA	43%	36%	p=1
RA	No features correlated to multiple metadata parameters	All features were restored by treatment	Not applicable
LVT	25%	50%	p=1
LVB	No features correlated to multiple metadata parameters	38%	p=1
RVT	55%	10%	p=0.012
RVB	No features correlated to multiple metadata parameters	28%	p=0.553

Supplementary Table 16. Correlation between disease metadata and small molecule peak area at 75 DPI in LVB heart section. N=15 mice per group and per position. Note that correlations are performed separately for each treatment effects, so the list of features that met our FDR-corrected p-value cutoff of <0.05 may not fully overlap between "Small molecules restored by any treatment" and small molecules restored by individual treatments. Source data are provided as a Source Data file. BNZ, benznidazole. FDR, false discovery rate. PC, glycerophosphocholine. LPC, lysoglycerophosphocholine.

Effect of treatment	Disease severity metadata	Small molecules	Annotation	Correlation	FDR_pval
Small molecules restored by any treatment	Liver_weight/Body_weight	X291.146_2.39	Didanosine_C10H12N4O3_(M+H+C4H6)	-0.7461	0.000387
	Liver_weight/Body_weight	X620.356_2.913	No annotation	-0.65606	0.003621
	Liver_weight/Body_weight	X604.362_2.948	PC 22:3;O_C30H54NO9P	-0.67297	0.003621
	Ejection_Fraction	X291.146_2.39	Didanosine_C10H12N4O3_(M+H+C4H6)	-0.65835	0.003621
	Ejection_Fraction	X494.325_2.865	LPC 16:1_C24H48NO7P or LPC O-16:2;O_C24H48NO7P	0.568551	0.036584
Restored only by BNZ + Tc24 vaccine treatment	Liver_weight/Body_weight	X604.362_2.948	PC 22:3;O_C30H54NO9P	-0.67297	0.004838
Restored only by BNZ treatment	Liver_weight/Body_weight	X291.146_2.39	Didanosine_C10H12N4O3_(M+H+C4H6)	-0.7461	9.29E-05
	Liver_weight/Body_weight	X620.356_2.913	No annotation	-0.65606	0.001159
	Ejection_Fraction	X291.146_2.39	Didanosine_C10H12N4O3_(M+H+C4H6)	-0.65835	0.001159
	Ejection_Fraction	X494.325_2.865	LPC 16:1_C24H48NO7P or LPC O-16:2;O_C24H48NO7P	0.568551	0.010975
	Ejection_Fraction	X386.717_2.856	No annotation	-0.52709	0.023222
	Liver_weight/Body_weight	X386.717_2.856	No annotation	-0.51117	0.023347
	P Amplitude (V)	X251.037_0.323	No annotation	0.515071	0.023347
	Liver_weight/Body_weight	X494.325_2.865	LPC 16:1_C24H48NO7P or LPC O-16:2;O_C24H48NO7P	0.488085	0.032622
Not restored by any treatments	Liver_weight/Body_weight	X136.062_0.301	No annotation	-0.72444	0.000609
	Ejection_Fraction	X682.363_3.329	No annotation	-0.73773	0.000609
	Liver_weight/Body_weight	X301.115_0.413	No annotation	-0.70525	0.000913
	Liver_weight/Body_weight	X476.24_2.164	No annotation	-0.69392	0.001072
	Ejection_Fraction	X137.046_0.35	Hypoxanthine_C5H4N4O	-0.68276	0.00131
	Liver_weight/Body_weight	X682.364_3.313	No annotation	0.670758	0.001687
	Liver_weight/Body_weight	X475.989_2.164	No annotation	-0.65863	0.001926
	Ejection_Fraction	X682.364_3.313	No annotation	0.658869	0.001926
	Liver_weight/Body_weight	X682.363_3.329	No annotation	-0.65201	0.002137
	Liver_weight/Body_weight	X301.115_0.328	No annotation	-0.63974	0.002861

Liver_weight/Body_weight	X137.046_0.42	No annotation	-0.63026	0.003495
Ejection_Fraction	X580.362_2.984	PC 20:1;O_C28H54NO9P	0.621978	0.004114
Liver_weight/Body_weight	X380.993_2.164	No annotation	-0.59561	0.007476
Ejection_Fraction	X203.007_0.309	No annotation	-0.59756	0.007476
Ejection_Fraction	X137.046_0.42	No annotation	-0.58932	0.007753
P Amplitude (V)	X301.115_0.413	No annotation	0.590605	0.007753
P Amplitude (V)	X136.062_0.301	No annotation	0.583222	0.008557
Ejection_Fraction	X301.115_0.328	No annotation	-0.58111	0.008557
Liver_weight/Body_weight	X136.097_0.551	No annotation	0.56922	0.010012
Liver_weight/Body_weight	X203.007_0.309	No annotation	-0.57157	0.010012
Ejection_Fraction	X136.062_0.301	No annotation	-0.56892	0.010012
Ejection_Fraction	X301.115_0.413	No annotation	-0.56515	0.010496
Ejection_Fraction	X475.989_2.164	No annotation	-0.55009	0.014019
P Amplitude (V)	X137.046_0.35	Hypoxanthine_C5H4N4O	0.549561	0.014019
P Amplitude (V)	X203.007_0.309	No annotation	0.543322	0.015567
Liver_weight/Body_weight	X324.327_3.455	Erucylamide	0.53661	0.017452
Ejection_Fraction	X476.24_2.164	No annotation	-0.53401	0.017822
Liver_weight/Body_weight	X137.046_0.35	Hypoxanthine_C5H4N4O	-0.52436	0.021277
P Amplitude (V)	X137.046_0.42	No annotation	0.488597	0.043062

Supplementary Table 17. Correlation between disease metadata and small molecule peak area at 75 DPI in RVB heart section. N=15 mice per group and per position. Note that correlations are performed separately for each treatment effects, so the list of features that met our FDR-corrected p-value cutoff of <0.05 may not fully overlap between "Small molecules restored by any treatment" and small molecules restored by individual treatments. Source data are provided as a Source Data file. BNZ, benznidazole. FDR, false discovery rate. PC, glycerophosphocholine. LPC, lysoglycerophosphocholine.

Effect of treatment	Disease severity metadata	Small molecules	Annotation	Correlation	FDR_pval
Small molecules restored by any treatment	Liver_weight/Body_weight	X568.341_2.887	LPC 22:6_C30H50NO7P or PC O-22:6_C30H50NO7P	0.599555	0.034157
	Liver_weight/Body_weight	X590.322_2.887	No annotation	0.593764	0.034157
	Liver_weight/Body_weight	X132.102_0.307	No annotation	0.615128	0.034157
	P Amplitude (V)	X568.341_2.887	LPC 22:6_C30H50NO7P or PC O-22:6_C30H50NO7P	-0.57604	0.040867
Restored only by BNZ treatment	Liver_weight/Body_weight	X568.341_2.887	LPC 22:6_C30H50NO7P or PC O-22:6_C30H50NO7P	0.599555	0.015181
	Liver_weight/Body_weight	X590.322_2.887	No annotation	0.593764	0.015181
	P Amplitude (V)	X568.341_2.887	LPC 22:6_C30H50NO7P or PC O-22:6_C30H50NO7P	-0.57604	0.016145
	P Amplitude (V)	X522.357_2.957	LPC 18:1_C26H52NO7P or PC O-18:1_C26H52NO7P or LPC O-18:2;O_C26H52NO7P	-0.52909	0.037031
Restored by both treatment	Liver_weight/Body_weight	X132.102_0.307	No annotation	0.615128	0.012496
Not restored by any treatments	Liver_weight/Body_weight	X150.059_0.3	Methionine_C5H11NO2S	0.650568	0.024341
	Liver_weight/Body_weight	X303.648_2.884	No annotation	0.628398	0.024553

Supplementary Table 18. Correlation between disease metadata and small molecule peak area at 142 DPI in LA heart section. N=15 mice per group and per position. Note that correlations are performed separately for each treatment effects, so the list of features that met our FDR-corrected p-value cutoff of <0.05 may not fully overlap between “Small molecules restored by any treatment” and small molecules restored by individual treatments. Source data are provided as a Source Data file. BNZ, benznidazole. FDR, false discovery rate. PC, glycerophosphocholine. LPC, lysoglycerophosphocholine.

Effect of treatment	Disease severity metadata	Small molecules	Annotation	Correlation	FDR_pval
Small molecules restored by any treatment	QTc (s)	X679.068_2.179	No annotation	0.622574	0.020068
	Liver_weight/Body_weight	X385.165_2.771	No annotation	0.598082	0.020239
	Liver_weight/Body_weight	X679.068_2.179	No annotation	0.57173	0.020615
	PR Interval (s)	X679.068_2.179	No annotation	0.571054	0.020615
	Liver_weight/Body_weight	X387.192_2.843	No annotation	-0.54941	0.027941
	Liver_weight/Body_weight	X616.308_2.766	No annotation	0.539232	0.028487
	CD3.CD8.IFNg.	X114.092_1.761	3-Aminocaproic acid_C6H13NO2	0.533942	0.028487
	PR Interval (s)	X114.092_1.761	3-Aminocaproic acid_C6H13NO2	-0.5247	0.030588
	CD3.CD8.IFNg.	X608.393_3.098	PC 22:1;O_C30H58NO9P	0.516079	0.032743
	Liver_weight/Body_weight	X377.146_2.225	Riboflavin_C17H20N4O6	-0.49759	0.043217
	Liver_weight/Body_weight	X608.393_3.098	PC 22:1;O_C30H58NO9P	-0.48604	0.049388
Restored only by BNZ + Tc24 vaccine treatment	Liver_weight/Body_weight	X377.146_2.225	Riboflavin_C17H20N4O6	-0.49759	0.036014
Restored only by BNZ treatment	Liver_weight/Body_weight	X616.308_2.766	No annotation	0.539232	0.036836
	CD3.CD8.IFNg.	X608.393_3.098	PC 22:1;O_C30H58NO9P	0.516079	0.036836
	Liver_weight/Body_weight	X608.393_3.098	PC 22:1;O_C30H58NO9P	-0.48604	0.045273
Restored by both treatments	QTc (s)	X679.068_2.179	No annotation	0.622574	0.013379
	Liver_weight/Body_weight	X385.165_2.771	No annotation	0.598082	0.013493
	Liver_weight/Body_weight	X679.068_2.179	No annotation	0.57173	0.013743
	PR Interval (s)	X679.068_2.179	No annotation	0.571054	0.013743
	Liver_weight/Body_weight	X387.192_2.843	No annotation	-0.54941	0.018628
	CD3.CD8.IFNg.	X114.092_1.761	3-Aminocaproic acid_C6H13NO2	0.533942	0.022157
	PR Interval (s)	X114.092_1.761	3-Aminocaproic acid_C6H13NO2	-0.5247	0.023305

Not restored by any treatment	Liver_weight/Body_weight	X137.046_0.35	Hypoxanthine_C5H4N4O	-0.6022	0.021252
	Liver_weight/Body_weight	X648.468_2.995	No annotation	0.606568	0.021252
	PR Interval (s)	X137.046_0.35	Hypoxanthine_C5H4N4O	-0.58783	0.021252
	P Amplitude (V)	X532.385_3.004	No annotation	-0.58763	0.021252
	Liver_weight/Body_weight	X650.403_3.014	PC 24:2;O2_C32H60NO10P	0.571511	0.025812
	Liver_weight/Body_weight	X532.385_3.004	No annotation	0.563337	0.026369
	Liver_weight/Body_weight	X516.39_3.018	No annotation	0.543746	0.029755
	Liver_weight/Body_weight	X604.442_3.001	No annotation	0.550523	0.029755
	P Amplitude (V)	X620.437_2.993	No annotation	-0.54119	0.029755
	Liver_weight/Body_weight	X560.416_3.006	No annotation	0.521915	0.031665
	Liver_weight/Body_weight	X620.437_2.993	No annotation	0.52907	0.031665
	P Amplitude (V)	X444.332_3.016	No annotation	-0.52447	0.031665
	P Amplitude (V)	X664.463_2.992	No annotation	-0.5233	0.031665
	Liver_weight/Body_weight	X576.411_2.999	No annotation	0.514435	0.033494
	Liver_weight/Body_weight	X664.463_2.992	No annotation	0.509506	0.033494
	P Amplitude (V)	X576.411_2.999	No annotation	-0.5111	0.033494
	QTc (s)	X137.046_0.35	Hypoxanthine_C5H4N4O	-0.50211	0.036721
	Liver_weight/Body_weight	X472.363_3.023	No annotation	0.496124	0.037089
	P Amplitude (V)	X708.489_2.982	No annotation	-0.49861	0.037089
	P Amplitude (V)	X666.433_3.092	PC 25:1;O2_C33H64NO10P	0.481719	0.046755

Supplementary Table 19. Correlation between disease metadata and small molecule peak area at 142 DPI in RA heart section. N=15 mice per group and per position. Note that correlations are performed separately for each treatment effects, so the list of features that met our FDR-corrected p-value cutoff of <0.05 may not fully overlap between “Small molecules restored by any treatment” and small molecules restored by individual treatments. Source data are provided as a Source Data file. BNZ, benznidazole. FDR, false discovery rate.

Effect of treatment	Disease severity metadata	Small molecules	Annotation	Correlation	FDR_pval
Small molecules restored by any treatment	Liver_weight/Body_weight	X263.091_2.771	No annotation	0.635334	0.016996
	Liver_weight/Body_weight	X296.258_2.976	No annotation	-0.647	0.016996
	Liver_weight/Body_weight	X295.227_3.017	No annotation	-0.60308	0.022098
	Liver_weight/Body_weight	X538.315_2.977	No annotation	-0.60296	0.022098
	Liver_weight/Body_weight	X137.046_0.35	Hypoxanthine_C5H4N4O	-0.56771	0.037362
	Liver_weight/Body_weight	X696.443_3.1	No annotation	0.573466	0.037362
	Liver_weight/Body_weight	X305.248_3.26	cis-5,8,11,14-Eicosatetraenoic acid_C20H32O2	0.554974	0.04372
Restored only by BNZ treatment	Liver_weight/Body_weight	X137.046_0.35	Hypoxanthine_C5H4N4O	-0.56771	0.022417
	Ejection_Fraction	X137.046_0.35	Hypoxanthine_C5H4N4O	-0.51591	0.024648
	P Amplitude (V)	X696.445_3.083	No annotation	0.520571	0.024648
Restored by both treatments	Liver_weight/Body_weight	X263.091_2.771	No annotation	0.635334	0.015296
	Liver_weight/Body_weight	X296.258_2.976	No annotation	-0.647	0.015296
	Liver_weight/Body_weight	X295.227_3.017	No annotation	-0.60308	0.019888
	Liver_weight/Body_weight	X538.315_2.977	No annotation	-0.60296	0.019888
	Liver_weight/Body_weight	X696.443_3.1	No annotation	0.573466	0.034909
	Liver_weight/Body_weight	X305.248_3.26	cis-5,8,11,14-Eicosatetraenoic acid_C20H32O2	0.554974	0.045906

Supplementary Table 20. Correlation between disease metadata and small molecule peak area at 142 DPI in LVT heart section. N=15 mice per group and per position. Note that correlations are performed separately for each treatment effects, so the list of features that met our FDR-corrected p-value cutoff of <0.05 may not fully overlap between "Small molecules restored by any treatment" and small molecules restored by individual treatments. Source data are provided as a Source Data file. BNZ, benznidazole. FDR, false discovery rate. CAR, acylcarnitine.

Effect of treatment	Disease severity metadata	Small molecules	Annotation	Correlation	FDR_pval
Small molecules restored by any treatment	Ejection_Fraction	X440.337_2.765	CAR 18:2;O_C25H45NO5	0.672242	0.004138
	P Amplitude (V)	X272.222_2.957	N-Lauroylsarcosine_C15H29NO3	0.679644	0.004138
	Liver_weight/Body_weight	X440.337_2.765	CAR 18:2;O_C25H45NO5	0.64704	0.006504
	Liver_weight/Body_weight	X366.373_3.699	Oleamide_C18H35NO_(M+H+C6H12)	0.572637	0.041262
	Liver_weight/Body_weight	X296.258_2.976	No annotation	-0.55854	0.046812
Restored only by BNZ + Tc24 vaccine treatment	P Amplitude (V)	X272.222_2.957	N-Lauroylsarcosine_C15H29NO3	0.679644	0.003546
Restored only by BNZ treatment	Ejection_Fraction	X440.337_2.765	CAR 18:2;O_C25H45NO5	0.672242	0.001324
	Liver_weight/Body_weight	X440.337_2.765	CAR 18:2;O_C25H45NO5	0.64704	0.001561
	Liver_weight/Body_weight	X203.007_0.309	No annotation	-0.52487	0.027088
	P Amplitude (V)	X293.211_2.933	trans-EKODE-(E)-Ib_C18H30O4	0.485651	0.045613
Restored by both treatments	Liver_weight/Body_weight	X296.258_2.976	No annotation	-0.55854	0.032768
	Liver_weight/Body_weight	X366.373_3.699	Oleamide_C18H35NO_(M+H+C6H12)	0.572637	0.032768
Not restored by any treatment	Liver_weight/Body_weight	X442.353_2.823	CAR 18:1;O_C25H47NO5	0.672949	0.000646
	Ejection_Fraction	X442.353_2.823	CAR 18:1;O_C25H47NO5	0.588102	0.004419
	Liver_weight/Body_weight	X240.116_2.324	No annotation	0.562088	0.005724

Supplementary Table 21. Correlation between disease metadata and small molecule peak area at 142 DPI in LVB heart section. N=15 mice per group and per position. Note that correlations are performed separately for each treatment effects, so the list of features that met our FDR-corrected p-value cutoff of <0.05 may not fully overlap between "Small molecules restored by any treatment" and small molecules restored by individual treatments. Source data are provided as a Source Data file. BNZ, benznidazole. FDR, false discovery rate. PC, glycerophosphocholine. LPC, lysoglycerophosphocholine. CAR, acylcarnitine.

Effect of treatment	Disease severity metadata	Small molecules	Annotation	Correlation	FDR_pval
Small molecules restored by any treatment	Liver_weight/Body_weight	X249.112_2.768	No annotation	0.625096	0.038764
Restored only by BNZ + Tc24 vaccine treatment	Liver_weight/Body_weight	X249.112_2.768	No annotation	0.625096	0.023259
Restored only by BNZ treatment	P Amplitude (V)	X291.146_2.39	Didanosine_C10H12N4O3_(M+H+C4H6)	0.574694	0.037595
	Liver_weight/Body_weight	X119.049_2.765	No annotation	0.523439	0.041921
	Liver_weight/Body_weight	X494.325_2.865	LPC 16:1_C24H48NO7P or LPC O-16:2;O_C24H48NO7P	0.527634	0.041921
Restored by both treatments	Liver_weight/Body_weight	X792.329_2.764	No annotation	0.584014	0.019692
	Liver_weight/Body_weight	X387.193_2.842	No annotation	-0.55604	0.019886
	Liver_weight/Body_weight	X811.309_2.773	No annotation	0.519417	0.030487
	Ejection_Fraction	X811.309_2.773	No annotation	0.469188	0.049883
	P Amplitude (V)	X792.329_2.764	No annotation	-0.47378	0.049883
Not restored by any treatment	Liver_weight/Body_weight	X682.363_3.329	No annotation	-0.71238	0.002042
	Liver_weight/Body_weight	X256.264_3.225	Palmitamide_C16H33NO	-0.67113	0.003469
	Ejection_Fraction	X416.337_2.79	CAR 16:0;O_C23H45NO5	0.669968	0.003469
	Liver_weight/Body_weight	X128.018_0.344	No annotation	0.645294	0.004533
	Liver_weight/Body_weight	X476.24_2.164	No annotation	-0.64133	0.004533
	Liver_weight/Body_weight	X682.364_3.313	No annotation	0.642567	0.004533
	Liver_weight/Body_weight	X475.989_2.164	No annotation	-0.62451	0.006537
	Liver_weight/Body_weight	X296.258_2.976	No annotation	-0.60151	0.007792
	Liver_weight/Body_weight	X380.993_2.164	No annotation	-0.60843	0.007792
	Liver_weight/Body_weight	X440.337_2.765	CAR 18:2;O_C25H45NO5	0.600116	0.007792
	Liver_weight/Body_weight	X442.353_2.823	CAR 18:1;O_C25H47NO5	0.602918	0.007792
	Liver_weight/Body_weight	X466.25_2.226	No annotation	-0.59972	0.007792
	P Amplitude (V)	X351.217_2.741	No annotation	0.57269	0.014708

Liver_weight/Body_weight	X137.046_0.35	Hypoxanthine_C5H4N4O	-0.55591	0.018702
Liver_weight/Body_weight	X416.337_2.79	CAR 16:0;O_C23H45NO5	0.547621	0.018702
Ejection_Fraction	X440.337_2.765	CAR 18:2;O_C25H45NO5	0.547234	0.018702
Ejection_Fraction	X442.353_2.823	CAR 18:1;O_C25H47NO5	0.556979	0.018702
PR Interval (s)	X440.337_2.765	CAR 18:2;O_C25H45NO5	0.548586	0.018702
CD3.CD8.IFNg.	X256.264_3.225	Palmitamide_C16H33NO	0.552468	0.018702
Liver_weight/Body_weight	X458.23_2.165	No annotation	-0.54307	0.01957
Liver_weight/Body_weight	X136.062_0.301	No annotation	-0.53316	0.023352
Liver_weight/Body_weight	X580.362_2.984	PC 20:1;O_C28H54NO9P	0.52401	0.027288
CD3.CD8.IFNg.	X136.097_0.551	No annotation	-0.52034	0.028264
Ejection_Fraction	X256.264_3.225	Palmitamide_C16H33NO	-0.50894	0.034487
Liver_weight/Body_weight	X136.097_0.551	No annotation	0.506943	0.03451
P Amplitude (V)	X580.362_2.984	PC 20:1;O_C28H54NO9P	-0.50096	0.037518
Liver_weight/Body_weight	X203.007_0.309	No annotation	-0.4885	0.043147
P Amplitude (V)	X420.899_2.103	No annotation	0.489322	0.043147
CD3.CD8.IFNg.	X476.24_2.164	No annotation	0.489205	0.043147
Liver_weight/Body_weight	X351.217_2.741	No annotation	-0.48664	0.043252
Liver_weight/Body_weight	X137.046_0.42	No annotation	-0.48403	0.044031

Supplementary Table 22. Correlation between disease metadata and small molecule peak area at 142DPI in RVT heart section. N=15 mice per group and per position. Note that correlations are performed separately for each treatment effects, so the list of features that met our FDR-corrected p-value cutoff of <0.05 may not fully overlap between “Small molecules restored by any treatment” and small molecules restored by individual treatments. Source data are provided as a Source Data file. BNZ, benznidazole. FDR, false discovery rate. PC, glycerophosphocholine.

Effect of treatment	Disease severity metadata	Small molecules	Annotation	Correlation	FDR_pval
Small molecules restored by any treatment	Liver_weight/Body_weight	X249.112_2.768	No annotation	0.709902	0.002419
	Liver_weight/Body_weight	X489.228_2.768	No annotation	0.646489	0.004105
	Liver_weight/Body_weight	X599.241_2.765	No annotation	0.652943	0.004105
	Ejection_Fraction	X249.112_2.768	No annotation	0.668012	0.004105
	Ejection_Fraction	X489.228_2.768	No annotation	0.647608	0.004105
	Ejection_Fraction	X792.329_2.764	No annotation	0.650838	0.004105
	Liver_weight/Body_weight	X200.165_3.207	No annotation	-0.63854	0.004538
	Ejection_Fraction	X369.17_2.767	No annotation	0.628921	0.005351
	Liver_weight/Body_weight	X111.044_2.768	No annotation	0.623457	0.00561
	Liver_weight/Body_weight	X792.329_2.764	No annotation	0.612692	0.006423
	Ejection_Fraction	X599.241_2.765	No annotation	0.612013	0.006423
	Ejection_Fraction	X111.044_2.768	No annotation	0.591684	0.010371
	Liver_weight/Body_weight	X130.087_0.452	No annotation	-0.5844	0.011621
	Liver_weight/Body_weight	X175.148_2.71	No annotation	-0.5726	0.014631
	QTc (s)	X198.15_2.471	No annotation	-0.56396	0.016947
	Liver_weight/Body_weight	X198.15_2.471	No annotation	-0.5479	0.023371
	Liver_weight/Body_weight	X333.063_2.524	No annotation	-0.53477	0.029741
Restored only by BNZ + Tc24 vaccine treatment	Liver_weight/Body_weight	X130.087_0.452	No annotation	-0.5844	0.02643
	Liver_weight/Body_weight	X175.148_2.71	No annotation	-0.5726	0.02643
Restored by both treatments	Liver_weight/Body_weight	X249.112_2.768	No annotation	0.709902	0.001717
	Liver_weight/Body_weight	X489.228_2.768	No annotation	0.646489	0.002913
	Liver_weight/Body_weight	X599.241_2.765	No annotation	0.652943	0.002913
	Ejection_Fraction	X249.112_2.768	No annotation	0.668012	0.002913
	Ejection_Fraction	X489.228_2.768	No annotation	0.647608	0.002913
	Ejection_Fraction	X792.329_2.764	No annotation	0.650838	0.002913
	Liver_weight/Body_weight	X200.165_3.207	No annotation	-0.63854	0.00322
	Ejection_Fraction	X369.17_2.767	No annotation	0.628921	0.003797
	Liver_weight/Body_weight	X111.044_2.768	No annotation	0.623457	0.003981

	Liver_weight/Body_weight	X792.329_2.764	No annotation	0.612692	0.004558
	Ejection_Fraction	X599.241_2.765	No annotation	0.612013	0.004558
	Ejection_Fraction	X111.044_2.768	No annotation	0.591684	0.00736
	QTc (s)	X198.15_2.471	No annotation	-0.56396	0.013877
	Liver_weight/Body_weight	X198.15_2.471	No annotation	-0.5479	0.018955
	Liver_weight/Body_weight	X333.063_2.524	No annotation	-0.53477	0.023921
	Liver_weight/Body_weight	X216.16_2.471	No annotation	-0.50313	0.044246
	Liver_weight/Body_weight	X604.362_2.948	PC 22:3;O_C30H54NO9P	-0.49922	0.045094
Not restored by any treatment	Liver_weight/Body_weight	X137.046_0.35	Hypoxanthine_C5H4N4O	-0.73039	0.000617
	Liver_weight/Body_weight	X278.248_3.1	No annotation	-0.72638	0.000617
	Liver_weight/Body_weight	X280.264_3.159	No annotation	-0.69456	0.001539
	Liver_weight/Body_weight	X268.105_0.35	Adenosine_C10H13N5O4	-0.6551	0.004786
	Liver_weight/Body_weight	X228.196_2.71	No annotation	-0.6298	0.008604
	Liver_weight/Body_weight	X256.227_2.847	No annotation	-0.6229	0.008833
	Liver_weight/Body_weight	X242.212_2.78	No annotation	-0.59375	0.013134
	Liver_weight/Body_weight	X296.259_2.971	No annotation	-0.6002	0.013134
	Liver_weight/Body_weight	X306.279_2.989	No annotation	-0.58729	0.013134
	Ejection_Fraction	X269.088_0.35	Inosine_C10H12N4O5	-0.58843	0.013134
	P Amplitude (V)	X272.222_2.957	N-Lauroylsarcosine_C15H29NO3	0.593771	0.013134
	Liver_weight/Body_weight	X306.279_3.212	No annotation	-0.57016	0.018742
	Liver_weight/Body_weight	X308.295_3.045	No annotation	-0.56059	0.021925
	Liver_weight/Body_weight	X258.207_2.775	No annotation	-0.54813	0.027175
	Liver_weight/Body_weight	X282.28_3.246	Oleamide_C18H35NO	-0.54278	0.027175
	Liver_weight/Body_weight	X308.295_3.285	No annotation	-0.54323	0.027175
	Liver_weight/Body_weight	X399.395_3.152	No annotation	0.539657	0.027463
	Liver_weight/Body_weight	X811.31_2.767	No annotation	0.537175	0.027463
	Ejection_Fraction	X399.395_3.152	No annotation	0.533812	0.028069
	Liver_weight/Body_weight	X399.394_3.159	No annotation	-0.52046	0.035771
	P Amplitude (V)	X811.309_2.773	No annotation	0.513207	0.039769
	Ejection_Fraction	X137.046_0.35	Hypoxanthine_C5H4N4O	-0.50078	0.049107

Supplementary Table 23. Correlation between disease metadata and small molecule peak area at 142 DPI in RVB heart section. N=15 mice per group and per position. Note that correlations are performed separately for each treatment effects, so the list of features that met our FDR-corrected p-value cutoff of <0.05 may not fully overlap between “Small molecules restored by any treatment” and small molecules restored by individual treatments. Source data are provided as a Source Data file. BNZ, benznidazole. FDR, false discovery rate. PC, glycerophosphocholine. LPC, lysoglycerophosphocholine.

Effect of treatment	Disease severity metadata	Small molecules	Annotation	Correlation	FDR_pval
Small molecules restored by any treatment	Ejection_Fraction	X142.035_0.315	No annotation	-0.59822	0.044391
	Ejection_Fraction	X214.181_2.634	No annotation	-0.58665	0.044391
	Ejection_Fraction	X300.199_2.264	No annotation	-0.58855	0.044391
Restored only by BNZ + Tc24 vaccine treatment	Ejection_Fraction	X142.035_0.315	No annotation	-0.59822	0.02143
	Ejection_Fraction	X214.181_2.634	No annotation	-0.58665	0.02143
	Ejection_Fraction	X300.199_2.264	No annotation	-0.58855	0.02143
	Liver_weight/Body_weight	X795.335_2.768	No annotation	0.553911	0.036624
	Liver_weight/Body_weight	X300.199_2.264	No annotation	-0.54172	0.038991
	Liver_weight/Body_weight	X308.295_3.045	No annotation	-0.52454	0.047753
Not restored by any treatment	Liver_weight/Body_weight	X528.311_2.928	No annotation	-0.72462	0.001459
	Liver_weight/Body_weight	X790.38_2.768	No annotation	0.678091	0.004692
	Liver_weight/Body_weight	X111.044_2.768	No annotation	0.655559	0.005823
	Liver_weight/Body_weight	X137.046_0.35	Hypoxanthine_C5H4N4O	-0.64404	0.005823
	Liver_weight/Body_weight	X249.112_2.768	No annotation	0.640246	0.005823
	Liver_weight/Body_weight	X489.228_2.768	No annotation	0.639368	0.005823
	Liver_weight/Body_weight	X105.07_2.767	No annotation	0.609102	0.006842
	Liver_weight/Body_weight	X267.123_2.767	No annotation	0.614221	0.006842
	Liver_weight/Body_weight	X268.105_0.35	Adenosine_C10H13N5O4	-0.62056	0.006842
	Liver_weight/Body_weight	X285.133_2.768	No annotation	0.613108	0.006842
	Liver_weight/Body_weight	X369.17_2.767	No annotation	0.613775	0.006842
	Liver_weight/Body_weight	X404.207_2.768	No annotation	0.628686	0.006842
	Liver_weight/Body_weight	X406.154_2.765	No annotation	0.608212	0.006842
	Liver_weight/Body_weight	X387.181_2.767	No annotation	0.604429	0.00707
	Liver_weight/Body_weight	X486.098_2.765	No annotation	0.595812	0.008376
	Liver_weight/Body_weight	X511.21_3.171	No annotation	-0.59039	0.008558
	Liver_weight/Body_weight	X599.241_2.765	No annotation	0.591364	0.008558
	Liver_weight/Body_weight	X269.088_0.35	Inosine_C10H12N4O5	-0.58818	0.008573

Liver_weight/Body_weight	X306.279_3.212	No annotation	-0.58418	0.00903
Liver_weight/Body_weight	X303.648_2.884	No annotation	-0.57563	0.010706
Liver_weight/Body_weight	X608.227_2.768	No annotation	0.568927	0.012081
Liver_weight/Body_weight	X430.223_2.768	No annotation	0.566704	0.012189
Liver_weight/Body_weight	X475.989_2.164	No annotation	-0.56371	0.012555
Liver_weight/Body_weight	X415.14_2.768	No annotation	0.561478	0.01271
Liver_weight/Body_weight	X446.218_2.884	No annotation	0.553466	0.013709
Liver_weight/Body_weight	X637.306_3.32	No annotation	-0.55525	0.013709
Liver_weight/Body_weight	X792.329_2.764	No annotation	0.553678	0.013709
Liver_weight/Body_weight	X278.248_3.1	No annotation	-0.54946	0.014536
Ejection_Fraction	X278.248_3.1	No annotation	-0.54216	0.016637
Ejection_Fraction	X269.088_0.35	Inosine_C10H12N4O5	-0.5208	0.025788
Ejection_Fraction	X280.264_3.159	No annotation	-0.51947	0.025788
Liver_weight/Body_weight	X280.264_3.159	No annotation	-0.51541	0.027244
Liver_weight/Body_weight	X476.24_2.164	No annotation	-0.50751	0.030266
Liver_weight/Body_weight	X570.357_2.934	LPC 22:5_C30H52NO7P or LPC 22:5_C30H52NO7P or LPC O-22:6;O_C30H52NO7P or PC O-22:5_C30H52NO7P	-0.50868	0.030266
Ejection_Fraction	X654.332_3.32	No annotation	-0.50389	0.03168
Ejection_Fraction	X637.306_3.32	No annotation	-0.49588	0.035253
QTc (s)	X303.648_2.884	No annotation	-0.49604	0.035253
P Amplitude (V)	X476.24_2.164	No annotation	0.491619	0.037369
Liver_weight/Body_weight	X654.332_3.32	No annotation	-0.48804	0.039071
Ejection_Fraction	X511.21_3.171	No annotation	-0.48183	0.042973
QTc (s)	X111.044_2.768	No annotation	0.473831	0.04881

Supplementary References

1 Schubert, M., Lindgreen, S. & Orlando, L. AdapterRemoval v2: rapid adapter trimming, identification, and read merging. *BMC Res Notes* **9**, 88, doi:10.1186/s13104-016-1900-2 (2016).

2 Patro, R., Duggal, G., Love, M. I., Irizarry, R. A. & Kingsford, C. Salmon provides fast and bias-aware quantification of transcript expression. *Nat Methods* **14**, 417-419, doi:10.1038/nmeth.4197 (2017).

3 Soneson, C., Love, M. I. & Robinson, M. D. Differential analyses for RNA-seq: transcript-level estimates improve gene-level inferences. *F1000Research* **4**, 1521, doi:10.12688/f1000research.7563.2 (2015).

4 Love, M. I., Huber, W. & Anders, S. Moderated estimation of fold change and dispersion for RNA-seq data with DESeq2. *Genome Biol* **15**, 550, doi:10.1186/s13059-014-0550-8 (2014).

5 Deng, K. *et al.* WaveICA 2.0: a novel batch effect removal method for untargeted metabolomics data without using batch information. *Metabolomics* **17**, 87, doi:10.1007/s11306-021-01839-7 (2021).

6 Bolyen, E. *et al.* Reproducible, interactive, scalable and extensible microbiome data science using QIIME 2. *Nat Biotechnol* **37**, 852-857, doi:10.1038/s41587-019-0209-9 (2019).
