# Supplementary material for: Localized cardiac small molecule trajectories and persistent chemical sequelae in experimental Chagas disease
Source: Nat Commun. 2023 Oct 25;14:6769. doi: 10.1038/s41467-023-42247-w (PMC10600178; doi:10.1038/s41467-023-42247-w)
Supplement: Supplementary file 3 — Description of Additional Supplementary Files [file 41467_2023_42247_MOESM3_ESM.pdf]

Description of additional supplementary files

Supplementary Data 1. Small molecule features impacted by infection over time. N=15 mice per group and per position. Source data are provided as a Source Data file.

Supplementary Data 2. Differentially-expressed genes based on RNA-seq. N=5/group. Source data are provided as a Source Data file. Statistical testing using likelihood-ratio test to determine if "condition" has an effect on gene counts. Pairwise Wald tests to determine which condition "levels" contribute to the difference. All tests are two-sided. P-value correction: BenjaminiHochberg False Discovery Rate (BH FDR). lfcSE, standard error of log2 fold change

Supplementary Data 3. Small molecule features impacted by treatment. N=15 mice per group and per position. Source data are provided as a Source Data file.
