# Supplementary material for: Localized cardiac small molecule trajectories and persistent chemical sequelae in experimental Chagas disease
Source: Nat Commun. 2023 Oct 25;14:6769. doi: 10.1038/s41467-023-42247-w (PMC10600178; doi:10.1038/s41467-023-42247-w)
Supplement: Supplementary file 7 — Reporting Summary [file 41467_2023_42247_MOESM7_ESM.pdf]

nature portfolio

Corresponding author(s): Jones, McCall

Last updated by author(s): Sep 26, 2023

Reporting Summary

Nature Portfolio wishes to improve the reproducibility of the work that we publish. This form provides structure for consistency and transparency in reporting. For further information on Nature Portfolio policies, see our Editorial Policies and the Editorial Policy Checklist.

Statistics

For all statistical analyses, confirm that the following items are present in the figure legend, table legend, main text, or Methods section.

n/a	Confirmed	
☐	☒	The exact sample size (*n*) for each experimental group/condition, given as a discrete number and unit of measurement
☐	☒	A statement on whether measurements were taken from distinct samples or whether the same sample was measured repeatedly
☐	☒	The statistical test(s) used AND whether they are one- or two-sided<br>*Only common tests should be described solely by name; describe more complex techniques in the Methods section.*
☐	☒	A description of all covariates tested
☐	☒	A description of any assumptions or corrections, such as tests of normality and adjustment for multiple comparisons
☐	☒	A full description of the statistical parameters including central tendency (e.g. means) or other basic estimates (e.g. regression coefficient) AND variation (e.g. standard deviation) or associated estimates of uncertainty (e.g. confidence intervals)
☐	☒	For null hypothesis testing, the test statistic (e.g. *F*, *t*, *r*) with confidence intervals, effect sizes, degrees of freedom and *P* value noted<br>*Give P values as exact values whenever suitable.*
☒	☐	For Bayesian analysis, information on the choice of priors and Markov chain Monte Carlo settings
☒	☐	For hierarchical and complex designs, identification of the appropriate level for tests and full reporting of outcomes
☐	☒	Estimates of effect sizes (e.g. Cohen's *d*, Pearson's *r*), indicating how they were calculated

Our web collection on statistics for biologists contains articles on many of the points above.

Software and code

Policy information about availability of computer code

Data collection

Echocardiography acquisition: Vevo 2100 software version 1.6.0 (build 6078)
Electrocardiogram acquisition: Tracings acquired on a Mouse Monitor connected to a Power Lab System and LabChart Pro software, version 8
Flow cytometry: BD FACSDiva software v9.0
qPCR: LightCycler 96 SW 1.1 (Roche)
LC-MS: XCalibur and Tune software (ThermoScientific)

Data analysis

Electrocardiogram/echocardiograms: VevoLab software (Fujifilm Visualsonics) and LabChart Pro
Flow cytometry: FlowJo 10.8.1
qPCR: LightCycler 96 SW 1.1 (Roche) and Microsoft Excel
LC-MS: MS Raw data were converted to mzXML format by MSConvert software. Converted mzXML files were processed in MZmine version 2.53. Total ion current (TIC) normalization was performed in Jupyter Notebook using R version 3.6.1. Principal coordinates analysis (PCoA) and PERMANOVA analyses were performed in QIIME and visualized in EMPeror.
UpSet plots version 0.6 were implemented in Python 3.8.
Boxplots and heatmaps were generated using Python 3.8.
Batch correction (supplementary figure 13 only) was performed using WaveICA 2.0.
MolNetEnhancer and Feature-based molecular networks were created using the Global Natural Products Social Molecular Networking platform (GNPS). All reported annotations were collected by an automated script from the GNPS output (https://github.com/camilgosmanov/GNPS).
Pairwise correlation between disease parameters and restored or not-restored small molecules was calculated using pandas.DataFrame.corr in Pandas python package. FDR-corrected p values were obtained using statsmodels.stats.multitest.fdrcorrection from the statsmodels 0.14.0

python package. Correlation data was visualized using Cytoscape version 3.9.1.
Fisher's exact test was calculated using https://www.socscistatistics.com/tests/fisher/default2.aspx.
Glycerophosphocholines (PC), glycerophosphoethanolamines (PE) and acylcarnitines (CAR) were extracted from LC-MS/MS data using Mass Spec Query Language (MassQL), implemented in the GNPS framework. Detailed query text is provided in the manuscript.
3D model figures were generated using 'ili.
Fig. 4a and Fig. 7 were created with BioRender.com.
RNA-seq data was quality filtered using AdapterRemoval (v2). Transcript quantification was performed using Salmon (v1.1.0). The resulting quantification data was imported into R ('tximport'), followed by gene expression analysis using DESeq2 4.
Representative code has been deposited in GitHub: https://github.com/zyliu-OU/McCall-Lab/tree/main/03172021.
Code and source data needed to reproduce manuscript figures have been deposited in GitHub at: https://github.com/zyliu-OU/03172021-source-data.

For manuscripts utilizing custom algorithms or software that are central to the research but not yet described in published literature, software must be made available to editors and reviewers. We strongly encourage code deposition in a community repository (e.g. GitHub). See the Nature Portfolio guidelines for submitting code & software for further information.

Data

Policy information about availability of data

All manuscripts must include a data availability statement. This statement should provide the following information, where applicable:

- Accession codes, unique identifiers, or web links for publicly available datasets
- A description of any restrictions on data availability
- For clinical datasets or third party data, please ensure that the statement adheres to our policy

The metabolomics data generated in this study have been deposited in the MassIVE database under accession codes MSV000092090 [doi:10.25345/C5C824Q8C] and MSV000087427 [doi:10.25345/C5T23S]. The RNA-seq data generated in this study have been deposited in SRA, bioproject accession PRJNA670449. [https://www.ncbi.nlm.nih.gov/bioproject/PRJNA670449/]. Molecular networks can be accessed at https://gnps.ucsd.edu/ProteoSAFe/status.jsp?task=dd1ef14c8a964bfd8843da96aa957d89 (feature-based molecular network) and https://gnps.ucsd.edu/ProteoSAFe/status.jsp?task=3fefbc8549604d34954aba2a95ec79df (MolNetEnhancer 132). MassQL for lipids analysis can be accessed at https://gnps.ucsd.edu/ProteoSAFe/status.jsp?task=40c907b8ef5940ce9f81b096828543ab for PC, https://gnps.ucsd.edu/ProteoSAFe/status.jsp?task=ba03560c4a1f4e4d87aca8f45cab0cf1 for PE, https://gnps.ucsd.edu/ProteoSAFe/status.jsp?task=9e2a979e50cc4f38845b57998ece5513 for CAR. The LIPID MAPS database used to confirm lipid annotations can be accessed at: https://www.lipidmaps.org/ 147,148. The data generated in this study are provided in the Source Data file and at https://github.com/zyliu-OU/03172021-source-data (DOI: 10.5281/zenodo.8364719) 150.

Human research participants

Policy information about studies involving human research participants and Sex and Gender in Research.

Reporting on sex and gender	No human subjects
Population characteristics	No human subjects
Recruitment	No human subjects
Ethics oversight	No human subjects

Note that full information on the approval of the study protocol must also be provided in the manuscript.

Field-specific reporting

Please select the one below that is the best fit for your research. If you are not sure, read the appropriate sections before making your selection.

☒ Life sciences ☐ Behavioural & social sciences ☐ Ecological, evolutionary & environmental sciences

For a reference copy of the document with all sections, see nature.com/documents/nr-reporting-summary-flat.pdf

Life sciences study design

All studies must disclose on these points even when the disclosure is negative.

Sample size

For Supplementary Figure 13 only: No statistical method was used to predetermine sample size. Groups were constituted from the animals that survived to treatment initiation, and then to the endpoint, leading to 6 infected vehicle-treated mice, 5 infected BNZ-treated mice and 6 uninfected mice being available for this analysis.
For Supplementary Figure 14-16, Supplementary Table 1, and Supplementary Data 2: No statistical method was used to predetermine sample size. Groups were constituted from the animals that survived to treatment initiation, and then to the endpoint, leading to 5 mice per group being available for this analysis.
For the remainder of the manuscript: based on prior data evaluating antigen specific immune responses after therapeutic vaccination in mice,

we performed a power analysis and calculated that a group size of n=13 will provide 80% power and significance level of 0.05 (Wilcoxon-Mann-Whitney test). Two mice were added to each group in the event that any animals had to be removed from the study due to reaching humane endpoints prior to scheduled study endpoints. Thus, the final group size was n=15 mice per group.

Data exclusions

DNA could not be extracted from the atrial homogenate due to the limited sample weight/volume. LC-MS data were filtered to obtain MS1 scans that were present in at least three samples (indicating reproducibility between animals) and that were associated with MS2 spectra (to enable annotation), with good extracted ion chromatogram peak shape. Blank removal was performed, with a minimum threefold difference between blank and samples required for a
feature to be retained. For Supplementary Figure 13 only: a single outlier was observed by PCoA in the vehicle group and was excluded. Apart from this, no samples were excluded in the manuscript. One mouse in the BNZ group was observed to have higher parasite burden in Figure 4f. We confirmed that this outlier mouse did not impact conclusions by providing data both with and without this outlier in Supplementary Figures 8-12 and Supplementary Tables 5-12.

Replication

The major finding of this manuscript is the fact that BNZ treatment did not fully restore the cardiac small molecule profile. Results concurred between two independent experimentation cohorts, with in vivo experimentation performed independently at the University of Oklahoma and at Baylor College of Medicine and data acquisition performed separately for both cohorts (compare Figure 4 data to Supplementary Figure 13). This lack of restoration of the cardiac small molecule profile also concurs with findings from the analysis of urine samples from three independent mouse cohorts, in 65. Analysis of the impact of the combination treatment was performed in a single in vivo experimentation cohort.
Timecourse small molecule analysis was performed in a single in vivo experimentation cohort. However, findings with regards to the heart apex being most affected and lipids being impacted by infection concur with our prior single-timepoint analyses published in two independent studies, performed at two separate institutions (30 and 31).
Furthermore, the small molecule categories and pathways identified as impacted by infection and restored or not-restored by treatment concur with the RNA-seq data presented in Supplementary Figures 14-16, Supplementary Data 2 and Supplementary Table 1, which came from another independent infection cohort.

Randomization

Mice were randomly allocated to experimental groups. LC-MS/MS data acquisition was performed in random order.

Blinding

The investigators were not blinded to allocation during treatments. This was due to the fact that only the BNZ treatment group and the combo treatment group received BNZ, and only the combo treatment group received subcutaneous injections of the Tc24 vaccine. However, small molecule analysis and final phenotypic data analysis between experimental groups (see below) were performed by different investigators, and small molecule extraction was blinded to the treatment group.

Reporting for specific materials, systems and methods

We require information from authors about some types of materials, experimental systems and methods used in many studies. Here, indicate whether each material, system or method listed is relevant to your study. If you are not sure if a list item applies to your research, read the appropriate section before selecting a response.

Materials & experimental systems

n/a	Involved in the study	
☐	☒	Antibodies
☐	☒	Eukaryotic cell lines
☒	☐	Palaeontology and archaeology
☐	☒	Animals and other organisms
☒	☐	Clinical data
☒	☐	Dual use research of concern

Methods

n/a	Involved in the study	
☒	☐	ChIP-seq
☐	☒	Flow cytometry
☒	☐	MRI-based neuroimaging

Antibodies

Antibodies used

0.5 µg anti-CD3e Pacific Blue clone 145-2C11 (Biolegend, Cat#100334, Lot # B308532), 0.1 µg anti-CD4 Alexa Fluor 700 clone RM4-5 (eBioscience, Cat#56-0042-82 , Lot#2218011), 0.25 µg anti-CD8a PerCP-Cy5.5 clone 53-6.7l (BD Bioscience, Cat#551162, Lot#5345668), 0.15 µg anti-IFNγ APC clone XMG1.2 (eBioscience, Cat#17-7311-81 , Lot#4289682), 0.15 µg anti-IL-17A clone TC11-18H10.1(Biolegend, Cat#504133 , Lot# B380067), 0.3 µg anti-IL-2 Brilliant Violet 510 clone JES6-5H4(Biolegend, Cat#503833 , Lot# B320882), 0.15 µg anti-IL-4 PE-Cyanine7 clone BVD6-24G2 (eBioscience, Cat#25-7042-42 , Lot#2181838), and 0.1 µg anti-TNFα PE clone MP6-XT22 (eBioscience, Cat#12-7321-82 , Lot#2124591).

Validation

Brilliant Violet 711™ anti-mouse IL-17A Antibody, clone TC11-18H10.1 (Biolegend). Reactivity to mouse IL-17A was verified by the manufacturer, and each lot is quality control tested by intracellular immunofluorescent staining with flow cytometric analysis.
Citation: Wang C, Kang SG, Lee J, Sun Z, Kim CH. The roles of CCR6 in migration of Th17 cells and regulation of effector T-cell balance in the gut. Mucosal Immunol. 2009 Mar;2(2):173-83. doi: 10.1038/mi.2008.84. Epub 2009 Jan 7. PMID: 19129757; PMCID: PMC2709747.
https://d1spbj2x7qk4bg.cloudfront.net/en-us/products/brilliant-violet-711-anti-mouse-il-17a-antibody-12030?pdf=true&displayInline=true&leftRightMargin=15&topBottomMargin=15&filename=Brilliant%20Violet%20711%E2%84%A2%20antimouse%20IL-17A%20Antibody.pdf&v=20220615063137

Pacific Blue™ anti-mouse CD3ε Antibody, clone 145-2C11 (Biolegend). Reactivity to mouse CD3 was verified by the manufacturer, and each lot is quality control tested by intracellular immunofluorescent staining with flow cytometric analysis.
Citation: Chappaz S, Flueck L, Farr AG, Rolink AG, Finke D. Increased TSLP availability restores T- and B-cell compartments in adult IL-7 deficient mice. Blood. 2007 Dec 1;110(12):3862-70. doi: 10.1182/blood-2007-02-074245. Epub 2007 Aug 16. PMID: 17702899.
https://d1spbj2x7qk4bg.cloudfront.net/en-us/products/pacific-blue-anti-mouse-cd3epsilon-antibody-6370?pdf=true&displayInline=true&leftRightMargin=15&topBottomMargin=15&filename=Pacific%20Blue%E2%84%A2%20anti-mouse%20CD3%CE%B5%20Antibody.pdf&v=20220913063022

Anti-Mouse CD4 Alexa Fluor® 700, clone RM4-5 (eBioscience). Antibodies are validated by the manufacturer by Relative expression to ensure that the antibody binds to the antigen stated. and to validate functional applications for flow cytometry.
Citation: Chen L, Chen H, Ye J, Ge Y, Wang H, Dai E, Ren J, Liu W, Ma C, Ju S, Guo ZS, Liu Z, Bartlett DL. Intratumoral expression of interleukin 23 variants using oncolytic vaccinia virus elicit potent antitumor effects on multiple tumor models via tumor microenvironment modulation. Theranostics. 2021 May 3;11(14):6668-6681. doi: 10.7150/thno.56494. PMID: 34093846; PMCID: PMC8171085.
https://assets.thermofisher.com/TFS-Assets/LSG/Flyers/commitment-antibody-performance-flyer.pdf

Anti-Mouse IFN gamma, APC clone XMG1.2 (eBioscience). Antibodies were verified by the manufacturer by cell treatment to ensure the antibodies bind to the IFN gamma antigen, and to validate functional application in flow cytometry.
Citation: Fett C, Zhao J, Perlman S. Measurement of CD8 and CD4 T Cell Responses in Mouse Lungs. Bio Protoc. 2014;4(6):e1083. doi: 10.21769/bioprotoc.1083. Epub 2014 Mar 20. PMID: 27390762; PMCID: PMC4932852.
https://assets.thermofisher.com/TFS-Assets/LSG/Flyers/commitment-antibody-performance-flyer.pdf

PerCP-Cy™5.5 Rat Anti-Mouse CD8a, clone 53-6.7 (BD Bioscience). Antibodies were verified by the manufacturer for reactivity to mouse by QC testing and to validate functional application in flow cytometry.
Citation: Bierer BE, Sleckman BP, Ratnofsky SE, Burakoff SJ. The biologic roles of CD2, CD4, and CD8 in T-cell activation. Annu Rev Immunol. 1989;7:579-99. doi: 10.1146/annurev.iy.07.040189.003051. PMID: 2653377.
https://www.bdbiosciences.com/en-us/products/reagents/flow-cytometry-reagents/research-reagents/single-color-antibodies-ruo/percp-cy-5-5-rat-anti-mouse-cd8a.551162

Anti mouse TNFα PE, clone MP6-XT22 (eBioscience). Antibodies were verified for reactivity to mouse by the manufacturer, and for functional activity in flow cytometry.
Citation: Luo Y, Guo J, Jia W, Wu M, Yin F, Niu G, Shih DQ, Targan SR, Zhang X. TNF-Like Ligand 1 Aberrance Aggravates Nonalcoholic Steatohepatitis via M1 Macrophage Polarization. Oxid Med Cell Longev. 2021 Dec 31;2021:3877617. doi: 10.1155/2021/3877617. PMID: 35003513; PMCID: PMC8741351.
https://www.thermofisher.com/antibody/product/TNF-alpha-Antibody-clone-MP6-XT22-Monoclonal/12-7321-82

Anti-mouse IL-4 monoclonal antibody PE- Cyanine7, clone BVD6-24G2 (eBioscience). Antibodies were verified for reactivity to mouse by the manufacturer, and for functional activity in flow cytometry.
Citation: Song Y, Wang Z, Jiang J, Piao Y, Li L, Xu C, Piao H, Li L, Yan G. DEK-targeting aptamer DTA-64 attenuates bronchial EMTmediated
airway remodelling by suppressing TGF-β1/Smad, MAPK and PI3K signalling pathway in asthma. J Cell Mol Med. 2020 Dec;24(23):13739-13750. doi: 10.1111/jcmm.15942. Epub 2020 Oct 30. Erratum in: J Cell Mol Med. 2022 Aug;26(15):4388. PMID: 33124760; PMCID: PMC7754001.
https://www.thermofisher.com/antibody/product/IL-4-Antibody-clone-BVD6-24G2-Monoclonal/25-7042-42

Brilliant Violet 510™ anti-mouse IL-2 Antibody, clone JES6-5H4 (Biolegend). Reactivity to mouse IL-2 was verified by the manufacturer, and each lot is quality control tested by intracellular immunofluorescent staining with flow cytometric analysis.
Citation: Dikiy S, Li J, Bai L, Jiang M, Janke L, Zong X, Hao X, Hoyos B, Wang ZM, Xu B, Fan Y, Rudensky AY, Feng Y. A distal Foxp3 enhancer enables interleukin-2 dependent thymic Treg cell lineage commitment for robust immune tolerance. Immunity. 2021 May 11;54(5):931-946.e11. doi: 10.1016/j.immuni.2021.03.020. Epub 2021 Apr 9. PMID: 33838102; PMCID: PMC8317508.
https://www.biolegend.com/en-us/products/brilliant-violet-510-anti-mouse-il-2-antibody-9274

Eukaryotic cell lines

Policy information about cell lines and Sex and Gender in Research

Cell line source(s)	C2C12 mouse myoblasts (ATCC CRL-1772) were used to propogate Trypanosoma cruzi trypomastigotes. Trypanosoma cruzi strain Sylvio X10/4 was obtained from ATCC. Trypanosoma cruzi luminescent strain H1 was generated as described in the manuscript, by transfection with the thermostable red-shifted firefly luciferase gene PpyRE9h.
Authentication	C2C12 cell lines were not authenticated after receipt from the manufacturer. Parasite cell lines were only authenticated by verifying that strain behavior in the Chagas disease model was as-expected and that luminescence was observed (for luminescent strain H1).
Mycoplasma contamination	Cell lines were not tested for Mycoplasma contamination after receipt from the manufacturer
Commonly misidentified lines (See ICLAC register)	No commonly misidentified lines were used

Animals and other research organisms

Policy information about studies involving animals; ARRIVE guidelines recommended for reporting animal research, and Sex and Gender in Research

Laboratory animals	For Figures 13-16, Supplementary Data 2 and Supplementary Table 1: 5-week-old male Swiss Webster mice; rest of the manuscript: Female BALB/c mice aged 6-7 weeks old
Wild animals	The study did not involve wild animals.
Reporting on sex	We elected to perform the study reported in the main manuscript in female mice, to correlate with prior published results in female mice. Experiments reported in Supplementary Table 1, Supplementary Figures 13-16, and Supplementary Data 2 only used male mice, to correlate with prior experimentation in male mice.
Field-collected samples	Study did not involve samples collected from the field
Ethics oversight	This research complies with all relevant ethical regulations and was approved by the University of Oklahoma Institutional Animal Care and Use Committee under assurance number R17-035 (experiments leading to Supplementary Table 1, Supplementary Figures 13-16, and Supplementary Data 2) or by the Baylor College of Medicine Institutional Animal Care and Use Committee under assurance number D16-00475 (experiments leading to all other data).

Note that full information on the approval of the study protocol must also be provided in the manuscript.

Flow Cytometry

Plots

Confirm that:

☒ The axis labels state the marker and fluorochrome used (e.g. CD4-FITC).

☒ The axis scales are clearly visible. Include numbers along axes only for bottom left plot of group (a 'group' is an analysis of identical markers).

☒ All plots are contour plots with outliers or pseudocolor plots.

☒ A numerical value for number of cells or percentage (with statistics) is provided.

Methodology

Sample preparation	To prepare single cell splenocyte suspensions, spleens were mechanically dissociated through 70 µm cell strainers, red blood cells were lysed with ACK lysis solution (Gibco), then washed with RPMI supplemented with 10% fetal bovine serum (FBS), 1X Pen/Strep and L-Glutamine (cRPMI). Live cells were quantified using a Cellometer Auto 2000 and AOPI live/dead dye (Nexcelom), then adjusted to a final concentration of 1x107 cells/mL in cRPMI. For each sample, 1x106 live splenocytes were restimulated for 96 hours with 100 µg/mL recombinant Tc24-C4 protein or cRPMI (unstimulated) at 37°C, 5% CO2. As a positive control, splenocytes incubated for 6 hours with 20 ng/mL phorbol myristate acetate (PMA) (Sigma-Aldrich) and 1 mg/mL ionomycin (Sigma-Aldrich) were included.<br><br>To quantify antigen-specific cytokine-producing CD4+ and CD8+ cells, cells were restimulated as described, with the addition of 4.1 µg/mL Brefeldin A (BD Biosciences GolgiPlug, Cat# 555029, Lot# 2321493) for the last 6 hours of incubation. Restimulated splenocytes were collected, washed with 1X PBS, and stained with Live/Dead fixable blue viability dye (Thermo Fisher Scientific, Cat# L23105, Lot# 1914462), 0.5 µg anti-CD3e Pacific Blue clone 145-2C11 (Biolegend, Cat#100334, Lot # B308532), 0.1 µg anti-CD4 Alexa Fluor 700 clone RM4-5 (eBioscience, Cat#56-0042-82 , Lot#2218011), and 0.25 µg anti-CD8a PerCP-Cy5.5 clone 53-6.7l (BD Bioscience, Cat#551162, Lot#5345668). Splenocytes were then fixed with Cytofix/ Cytoperm (BD Biosciences) and permeabilized following manufacturer instructions. Permeabilized splenocytes were stained with 0.15 µg anti-IFNγ APC clone XMG1.2 (eBioscience, Cat#17-7311-81 , Lot#4289682), 0.15 µg anti-IL-17A clone TC11-18H10.1(Biolegend, Cat#504133 , Lot# B380067), 0.3 µg anti-IL-2 Brilliant Violet 510 clone JES6-5H4(Biolegend, Cat#503833 , Lot# B320882), 0.15 µg anti-IL-4 PE-Cyanine7 clone BVD6-24G2 (eBioscience, Cat#25-7042-42 , Lot#2181838), and 0.1 µg anti-TNFα PE clone MP6-XT22 (eBioscience, Cat#12-7321-82 , Lot#2124591).
Instrument	Samples were acquired on a LSR Fortessa Cell Analyzer (BD Biosciences).
Software	Data was acquired with BD FACSDiva software v9.0, and analyzed using FlowJo 10.8.1 software
Cell population abundance	25,000 events in a live gate were collected and analyzed for the percent of each target population.
Gating strategy	Cells were gated for total leukocytes based on forward and side scatter, then single cells based on FSC height by area, then SSC height by area. Within the single cell population, live CD3+ cells were gated based on viability dye exclusion and positive staining for CD3 surface antigen. Within the Live CD3+, cells were gated for positive staining for either CD4 or CD8 surface staining. Within either CD4+ or CD8+ populations, cells were gated for intracellular staining of IL-2, IL-4, IL-17, IFNg, or TNFa. The gating strategy is illustrated in the supplemental figure 4.

☒ Tick this box to confirm that a figure exemplifying the gating strategy is provided in the Supplementary Information.
